# Synthesis and evaluation of novel 1-(((6-substitutedbenzo[*d*]thiazol-2-yl)amino)(heteroaryl)methyl)naphthalen-2-ol as pesticidal agents

**DOI:** 10.1080/14756366.2022.2032687

**Published:** 2022-01-27

**Authors:** Junfeng Shang, Yuxin Li, Na Yang, Lixia Xiong, Baolei Wang

**Affiliations:** a State Key Laboratory of Elemento-Organic Chemistry, College of Chemistry, Nankai University, Tianjin, China; b Key Laboratory of Study and Discovery of Small Targeted Molecules of Hunan Province, School of Medicine, Hunan Normal University, Changsha, China

**Keywords:** Novel *β*-naphthol derivatives, synthesis, pesticidal activity, ryanodine receptor, structure-activity relationship

## Abstract

To discover new agrochemicals with prominent pesticidal properties, a series of novel *β*-naphthol derivatives containing benzothiazolylamino and various heteroaryl groups (**8a-q**) were efficiently synthesised *via* Betti reaction. The bioassay results showed that most of the synthesised compounds exhibited favourable insecticidal potentials, particularly towards oriental armyworm (50–100% at 200 mg·L^−1^) and diamondback moth (50–95% at 10 mg·L^−1^). Compounds **8 b**, **8f**, **8 g**, **8j**, **8k**, **8n**, and **8o** possessed LC_50_ values of 0.0988–5.8864 mg·L^−1^ against diamondback moth. Compounds **8i**, **8 l**, and **8 m** also displayed lethality rates of 30–90% against spider mite at the concentration of 100 mg·L^−1^. Overall, some compounds could be considered as new insecticidal/acaricidal leading structures for further investigation. The calcium imaging experiments revealed that **8 h**, **8i**, and **viii** could activate the release of calcium ions in insect (*M. separata*) central neurons at a higher concentration (50 mg·L^−1^). The SAR analysis provided valuable information for further structural modifications.

## Graphical abstract

**Figure:**
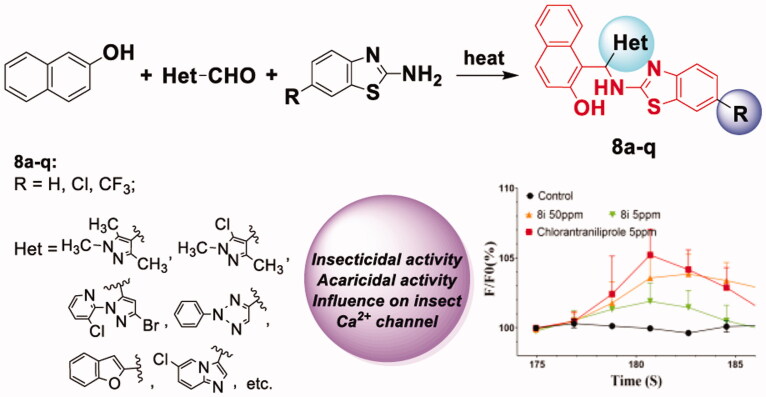

## 1. Introduction

Agrochemicals exert a vital role in guaranteeing the crop production of the world. According to the FAO of the United Nations, even with the application of agrochemicals, about 30% of the world potential crop production is still lost every year due to the influences of all kinds of crop pests, diseases and weeds^1^. But what is more striking is that these losses of crop production will be possibly doubled if there are no existing agrochemicals used. However, during the use of agrochemicals, some negative effects, such as eco-environmental pollution and resistance, have always been accompanied. Due to the dominance of such issues, the discovery and development of more novel agrochemicals with prominent pesticidal properties are urgently needed^2–4^.

**Figure 1.**
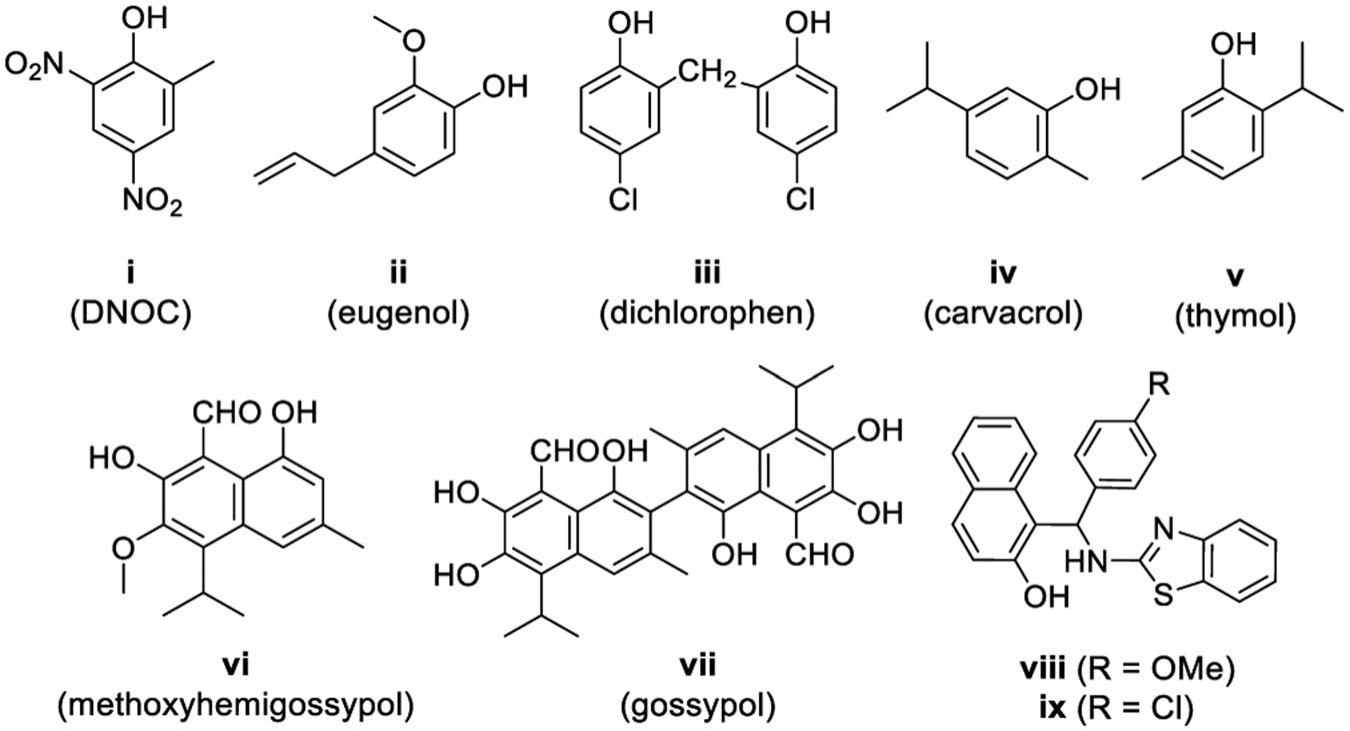
Chemical structures of some natural and synthesised phenolic derivatives possessing pesticidal activities.

The great potential of phenolic compounds in medicinal and pesticidal chemistry has been highly recognised. Many compounds containing phenol motifs have been found to possess a variety of pharmacological properties, such as antioxidant^5^^,^^6^, anti-inflammatory^6^, anticancer^7^, antiproliferative^8^, antitumor^8^, cytotoxic^9^ and antibacterial^9^ activities. Moreover, many phenols are important intermediates for producing commercial agrochemicals. Some phenolic derivatives also exhibit promising pesticidal activities. For examples, DNOC (Figure 1, **i**) can be used as a herbicidal and a fungicidal agent^10^^,^^11^; eugenol (Figure 1, **ii**) is a natural acaricide and fungicide^12^^,^^13^; dichlorophen and carvacrol (Figure 1, **iii** and **iv**) have effective fungicidal activities^14^^,^^15^; thymol (Figure 1, **v**) was recently reported to have promising insecticidal activity against *Caenorhabditis elegans* at different stages of growth and development^16^. Comparatively, there are seldom reports about a naphthol moiety being a part of the potent insecticidal agents, except those several representative natural products, such as methoxyhemigossypol and gossypol (Figure 1, **vi** and **vii**), both of which have insecticidal and antifungal activities^17–19^.

Recently, some *β*-naphthol derivatives containing benzothiazolylamino and substituted phenyl groups (e. g. compound **viii** in Figure 1) drew our attention, because of their favourable antifeedant and insecticidal activities against *Spodoptera litura* and *Achaea janata* (L.)^20^. Compounds **viii** and **ix** (Figure 1) have also been found to have inhibitory activity against angiotensin-converting-enzyme as well as calcium channel blocking activity on mammal rats, as reported by Kalavagunta *et al*.^21^. Whereas, both - the exploration of pesticidal potentials of more new *β*-naphthol derivatives and the influence of the compounds on the activity of insect calcium channels - remain uncovered. It has been known that the ryanodine receptor (RyR), which can modulate the release of calcium ions in the insect neuronal cells, is one of the most important targets for the insecticide design during the recent 15 years. Several diamide compounds with potent RyR activating effect, e.g., flubendiamide and chlorantroniliprole, have been discovered and successfully developed as commercial insecticides owing to their excellent pesticidal properties^22^^,^^23^. In addition, it has also been known that heterocyclic compounds comprise a large proportion of pharmaceuticals and agrochemicals due to their versatile biological activities^24^^,^^25^. A common strategy in molecular design is that heterocyclic moieties are introduced as substitutions of phenyl rings to improve the biological properties of the parent compounds. Based on this information, a series of novel benzothiazolylamino-containing *β*-naphthol derivatives were designed and successfully synthesised by introduction of various heteroaryl groups. Their insecticidal and acaricidal activities towards a variety of agricultural pests were evaluated. In addition, the influence of this type of compounds on the insect calcium ion channels (RyR) was also investigated.

## 2. Experimental

### 2.1. Chemistry

The melting points were determined on an X-4 binocular microscope apparatus (uncorrected). ^1^H NMR spectra were determined at 400 MHz and ^13 ^C NMR spectra at 101 MHz using a Bruker AV 400 instrument with CDCl_3_ or DMSO-*d*_6_ as solvents, and tetramethylsilane as the internal standard. HRMS were determined on a Varian 7.0 T FTICR-MS instrument. The purity analysis of the synthesised compounds were conducted on an Agilent 1260-II high performance liquid chromatography (HPLC) apparatus. Column chromatography was conducted using 200–300 mesh silica gel. Reagents were of analytically pure grade. Solvents were dried and distilled using the standard procedure before use. Benzo[*d*]thiazol-2-amine, 6-chlorobenzo[*d*]thiazol-2-amine and 6-(trifluoromethyl)benzo[*d*]thiazol-2-amine were purchased from J&K Scientific Ltd. Naphthalen-2-ol and partial heteroaryl aldehydes were bought from Tianjin Heowns Business Licence and Bide Pharmatech Ltd. As the positive control for biological tests, chlorantraniliprole was prepared following the known literature procedure^26^^,^^27^. Another two bioassay controls that have the similar structural characteristic as *β*-naphthol − 1-((benzo[d]thiazol-2-ylamino)(4-methoxyphenyl)methyl)naphthalen-2-ol (**viii**) and 1-((benzo[d]thiazol-2-ylamino)(4-chlorophenyl)methyl)naphthalen-2-ol (**ix**), were prepared *via* the reported literature protocol^20^.

#### 2.1.1. Synthesis of the intermediate heteroaryl aldehydes 2a-d

##### 2.1.1.1. Synthesis of 5-chloro-1,3-disubstituted-1H-pyrazole-4-carbaldehydes 2a-b

According to the reported procedure, 2,5-disubstitued-2,4-dihydro-3*H*-pyrazol-3-ones **1a-b** were prepared *via* a cyclisation reaction of ethyl acetoacetate or ethyl trifluoroacetoacetate in the presence of methylhydrazine or phenylhydrazine in boiling ethanol^28^^,^^29^. **1a**: white solid, yield 69%, mp 121–122 °C (Lit.^29^ mp 122 °C). ^1^H NMR (400 MHz, CDCl_3_) *δ*: 3.27 (s, 3H), 3.18 (s, 2H), 2.10 (s, 3H); **1 b**: white solid, yield 75%, mp 191–193 °C (Lit.^30^ mp 193.0–193.5 °C). ^1^H NMR (400 MHz, CDCl_3_) *δ*: 7.80 (d, *J* = 8.0 Hz, 2H), 7.68 (d, *J* = 8.0 Hz, 2H), 7.42–7.49 (m, 5H), 7.37–7.39 (m, 1H), 5.90 (s, 1H), 3.71 (s, 2H). The product was obtained as a mixture of dihydropyrazolone and pyrazolol.

Using an improved method from the previous report^31^^,^^32^, various pyrazole aldehyde **2a-b** were synthesised smoothly. Phosphorus oxychloride (64 ml) was added dropwise to 23 ml of DMF at 0–10 °C under stirring. Thirty minutes later at this temperature, pyrazolone **1** (0.1 mol) was added in portions. The reaction system was then heated to 80–100 °C and stirred for 3–4 h. After cooling down to ambient temperature, the system was poured into ice-water (380 ml) and further stirred for 15 min, then extracted with ethyl acetate (3 × 20 ml). The organic phase was washed with saturated aq. solution of NaHCO_3_ (2 × 20 ml) and brine (2 × 20 ml) successively, and dried over anhydrous Na_2_SO_4_. After removal of solvent, the residue was recrystallized from petroleum ether and ethyl acetate or subjected for the silica gel column chromatography using ethyl acetate and petroleum ether as solvents (v/v 1:4) to afford pyrazole aldehydes **2a-b**. **2a**: white solid, R*_f_* 0.76 (petroleum ether/ethyl acetate 3:1), mp 77–78 °C, yield 82% (Lit.^31^ mp 78–79 °C), ^1^H NMR (400 MHz, CDCl_3_) *δ*: 9.86 (s, 1H), 3.83 (s, 3H), 2.46 (s, 3H); **2 b**: yellow sticky oil (it can slowly solidify upon prolonged storage), mp 32–34 °C, R*_f_* 0.73 (petroleum ether/ethyl acetate 3:1), yield 91% (Lit.^33^ mp 34–36 °C), ^1^H NMR (400 MHz, CDCl_3_) *δ*: 10.06 (s, 1H), 5.56–5.57 (m, 5H).

##### 2.1.1.2. Synthesis of 3-bromo-1-(3-chloropyridin-2-yl)-1H-pyrazole-5-carbaldehyde 2c

A 100 ml flask charged with a solution of pyrazole carboxylic ester **3** (0.66 g, 2.0 mmol) in THF (30 ml) was cooled to −5 °C by an ice-salt bath. Lithium aluminium hydride (0.076 g, 2.0 mmol) was added to the above solution in small portions maintaining the temperature of −5–5 °C. After that, the reaction system was stirred at room temperature overnight followed by addition of excess of Na_2_SO_4_·10H_2_O (10.0 g) to quench the reaction. The mixture was filtered and the filtrate was concentrated under reduced pressure. The residue was further purified *via* silica gel column chromatography using ethyl acetate and petroleum ether as solvents (v/v 1:4) to give intermediate alcohol **4** as a white solid with 72% yield and mp of 108–109 °C (lit.^34^ mp 109–111 °C). R*_f_* 0.30 (petroleum ether/ethyl acetate 3:1), ^1^H NMR (400 MHz, DMSO-*d*_6_) *δ*: 8.56 (d, *J* = 5.9 Hz, 1H), 8.26 (d, *J* = 8.1 Hz, 1H), 7.65 (dd, *J* = 8.1, 5.9 Hz, 1H), 6.58 (s, 1H), 5.36 (t, *J* = 5.8 Hz, 1H), 4.45 (d, *J* = 5.8 Hz, 2H).

The pyrazole aldehyde **2c** was prepared using an improved method based on the previous report^34^. To a solution of intermediate alcohol **4** (2.7 g, 9.4 mmol) in DCM (50 ml), a well-mixed powder of pyridinium chlorochromate (6.0 g, 28.1 mmol) and silica gel (200–300 mesh, 9.0 g) was added at room temperature. The reaction mixture was stirred overnight and the solvent was removed under reduced pressure. The residue was purified by column chromatography using ethyl acetate and petroleum ether as solvents (v/v 1:6) to afford pyrazole aldehyde **2c** as light yellow solid with 95% yield and mp of 103–104 °C (lit.^34^ mp 104–105 °C). R*_f_* 0.76 (petroleum ether/ethyl acetate 3:1), ^1^H NMR (400 MHz, DMSO-*d*_6_) *δ*: 9.80 (s, 1H), 8.58 (dd, *J* = 4.7, 1.4 Hz, 1H), 8.31 (dd, *J* = 8.1, 1.4 Hz, 1H), 7.73 (dd, *J* = 8.1, 4.7 Hz, 1H), 7.54 (s, 1H).

##### 2.1.1.3. Synthesis of 2-phenyl-2H-1,2,3-triazole-4-carbaldehyde 2d

A mixture of the intermediate 1-(2-phenyl-2*H*-1,2,3-triazol-4-yl)butane-1,2,3,4-tetraol (**5**) (8.0 g, 0.03 mol), sodium periodate (19.26 g, 0.09 mol) and water (250 ml) was stirred at room temperature for 24 h, then filtered. The solid was washed with water thoroughly, dried in air and recrystallized from petroleum ether to afford phenyltriazole carbaldehyde intermediate **2d** as light yellow crystals with 69% yield and mp of 66–67 °C (Lit.^35^ mp 68–69 °C). ^1^H NMR (400 MHz, CDCl_3_) *δ*: 10.22 (s, 1H), 8.27 (s, 1H), 8.14 (d, *J* = 8.0 Hz, 2H), 7.54 (t, *J* = 8.0 Hz, 2H), 7.45 (t, *J* = 8.0 Hz, 1H).

#### 2.1.2. Synthesis of the title compounds 8a-q

A 50 ml round-bottom flask equipped with a condenser and containing a well-mixed powder of naphthalen-2-ol (**6**, 1 mmol), 6-substitutedbenzo[*d*]thiazol-2-amine (**7**, 1 mmol) and heteroaryl aldehyde (**2**, 1.1 mmol) was put into a 115–125 °C oil-bath under stirring. The mixture was maintained at this temperature for 1–2 h. Methanol (15 ml) was added to the mixture when the temperature of oil-bath was decreased to 80 °C. The mixture was further stirred for 10 min. After cooling down to room temperature, the mixture was filtered and the residue was washed successively or submitted for silica gel column chromatography using ethyl acetate and petroleum ether (v/v 1:7) as solvents to afford compound **8**(**a-q**).

##### 2.1.2.1. 1-((Benzo[d]thiazol-2-ylamino)(1H-imidazol-2-yl)methyl)naphthalen-2-ol (8a)

White solid, yield 62%, R*_f_* 0.64 (dichloromethane/methanol 100:1), mp 277–280 °C. ^1^H NMR (400 MHz, DMSO-*d*_6_) *δ*: 9.97 (s, 1H), 9.02 (d, *J* = 8.4 Hz, 1H), 8.63 (s, 1H), 8.04 (d, *J* = 8.5 Hz, 1H), 7.84–7.94 (m, 2H), 7.74–7.82 (m, 2H), 7.66 (d, *J* = 8.7 Hz, 1H), 7.35–7.53 (m, 3H), 7.29 (t, *J* = 7.2 Hz, 1H), 7.08–7.16 (m, 1H), 6.85 (d, *J* = 8.8 Hz, 1H), 6.74 (s, 1H); ^13 ^C NMR (101 MHz, DMSO-*d*_6_) *δ* 164.9, 159.4, 155.8, 155.0, 148.0, 132.2, 131.1, 130.0, 129.2, 128.6, 126.9, 126.5, 124.5, 123.8, 123.5, 122.5, 119.6, 118.6, 117.0, 115.1, 48.6. HRMS (ESI): calcd for C_21_H_17_N_4_OS [M + H]^+^ 373.1123, found, 373.1116, the error 1.88 ppm.

##### 2.1.2.2. 1-((Benzo[d]thiazol-2-ylamino)(1H-pyrazol-5-yl)methyl)naphthalen-2-ol (8 b)

White solid, yield 67%, R*_f_* 0.63 (dichloromethane/methanol 100:1), mp 190–193 °C. ^1^H NMR (400 MHz, DMSO-*d*_6_) *δ*: 10.07 (s, 1H), 9.00 (d, *J* = 8.6 Hz, 1H), 8.02 (d, *J* = 8.5 Hz, 1H), 7.91 (d, *J* = 8.9 Hz, 1H), 7.82–7.87 (m, 2H), 7.79 (d, *J* = 7.1 Hz, 2H), 7.67 (d, *J* = 8.8 Hz, 1H), 7.55 (d, *J* = 7.8 Hz, 1H), 7.45 (d, *J* = 8.9 Hz, 1H), 7.40 (d, *J* = 7.3 Hz, 1H), 7.30 (d, *J* = 7.5 Hz, 1H), 7.09–7.15 (m, 1H), 6.89 (d, *J* = 8.8 Hz, 1H), 6.71 (s, 1H); ^13 ^C NMR (101 MHz, DMSO-*d*_6_) *δ* 166.4, 160.7, 155.5, 148.2, 132.6, 131.4, 130.7, 129.9, 129.3, 129.1, 128.0, 127.5, 127.1, 125.2, 124.0, 123.1, 119.8, 119.1, 117.3, 114.9, 31.5. HRMS (ESI): calcd for C_21_H_17_N_4_OS [M + H]^+^ 373.1123, found, 373.1112, the error 2.95 ppm.

##### 2.1.2.3. 1-((Benzo[d]thiazol-2-ylamino)(1,3,5-trimethyl-1H-pyrazol-4-yl)methyl)naphthalen-2-ol (8c)

White solid, yield 57%, R*_f_* 0.67 (dichloromethane/methanol 100:1), mp 179–181 °C. ^1^H NMR (400 MHz, DMSO-*d*_6_) *δ*: 9.97 (br, 1H), 8.60 (d, *J* = 6.4 Hz, 1H), 8.02 (d, *J* = 8.6 Hz, 1H), 7.74–7.79 (m, 3H), 7.68 (d, *J* = 8.2 Hz, 1H), 7.63 (d, *J* = 7.7 Hz, 1H), 7.40 (d, *J* = 7.1 Hz, 1H), 7.25–7.28 (m, 1H), 7.21 (d, *J* = 8.8 Hz, 1H), 7.12 (s, 1H), 6.98–7.11 (m, 1H), 3.56 (s, 3H), 1.93 (s, 3H), 1.87 (s, 3H); ^13 ^C NMR (101 MHz, DMSO-*d*_6_) *δ*: 165.9, 155.7, 153.5, 152.9, 144.2, 136.3, 135.1, 132.9, 131.0, 129.7, 128.1, 126.5, 125.8, 123.1, 122.8, 121.3, 118.9, 118.7, 115.8, 109.1, 48.3, 36.0, 13.1, 9.9. HRMS (ESI) calcd for C_24_H_23_N_4_OS [M + H]^+^ 415.1593, found 415.1584, the error 2.17 ppm.

##### 2.1.2.4. 1-((Benzo[d]thiazol-2-ylamino)(1-methyl-3-(trifluoromethyl)-1H-pyrazol-4-yl)methyl)naphthalen-2-ol (8d)

White solid, yield 68%, R*_f_* 0.65 (dichloromethane/methanol 100:1), mp 184–186 °C. ^1^H NMR (400 MHz, CDCl_3_) *δ*: 12.44 (br, 1H), 8.03 (d, *J* = 8.4 Hz, 1H), 7.79 (d, *J* = 8.0 Hz, 1H), 7.75 (d, *J* = 8.8 Hz, 1H), 7.49–7.60 (m, 3H), 7.47 (s, 1H), 7.27–7.37 (m, 3H), 7.11 (t, *J* = 7.6 Hz, 1H), 6.63 (s, 1H), 3.72 (s, 3H); ^13 ^C NMR (101 MHz, CDCl_3_) *δ*: 169.3, 153.7, 150.0, 138.9 (q, *J* = 36.36 Hz, Pyrazole), 132.8, 131.9, 130.6, 129.5, 129.0, 128.7, 127.4, 126.3, 123.3, 122.2, 121.8 (q, *J* = 269.67 Hz, CF_3_), 121.5, 119.7, 119.0, 117.7, 117.3, 110.0, 50.0, 39.6. HRMS (ESI) calcd for C_23_H_16_F_3_N_4_OS [M-H]^-^ 453.0997, found 453.1001, the error 0.88 ppm.

##### 2.1.2.5. 1-((Benzo[d]thiazol-2-ylamino)(5-chloro-1,3-dimethyl-1H-pyrazol-4-yl)methyl)naphthalen-2-ol (8e)

White solid, yield 83%, R*_f_* 0.09 (petroleum ether/ethyl acetate 2:1), mp 275–278 °C. ^1^H NMR (400 MHz, DMSO-*d*_6_) *δ*: 10.71 (s, 1H), 9.48 (s, 1H), 7.87–8.14 (m, 1H), 7.86 (d, *J* = 7.6 Hz, 1H), 7.65 (s, 1H), 7.49–7.52 (m, 2H), 7.39–7.44 (m, 2H), 7.34–7.37 (m, 1H), 7.25 (s, 1H), 7.01–7.04 (m, 1H), 6.77 (s, 1H), 3.74 (s, 3H), 1.82 (s, 3H); ^13 ^C NMR (101 MHz, DMSO-*d*_6_) *δ*: 169.0, 151.7, 149.2, 143.3, 132.7, 131.3, 130.0, 129.7, 129.4, 128.9, 127.6, 127.1, 126.1, 125.1, 124.1, 123.2, 122.3, 118.1, 115.3, 97.4, 33.9, 28.9, 12.5. HRMS (ESI) calcd for C_23_H_20_ClN_4_OS [M + H]^+^ 435.1046, found 435.1039, the error 1.61 ppm.

##### 2.1.2.6. 1-((Benzo[d]thiazol-2-ylamino)(5-chloro-1-phenyl-3-(trifluoromethyl)-1H-pyrazol-4-yl)methyl)naphthalen-2-ol (8f)

White solid, yield 92%, R*_f_* 0.38 (petroleum ether/ethyl acetate 2:1), mp 194–196 °C. ^1^H NMR (400 MHz, CDCl_3_) *δ*: 12.05 (br, 1H), 8.04 (d, *J* = 8.0 Hz, 1H), 7.77 (d, *J* = 8.0 Hz, 1H), 7.72 (d, *J* = 8.0 Hz, 1H), 7.62 (d, *J* = 8.0 Hz, 1H), 7.53–7.57 (m, 2H), 7.29–7.36 (m, 8H), 7.14 (d, *J* = 8.0 Hz, 1H), 6.89 (s, 1H); ^13 ^C NMR (101 MHz, CDCl_3_) *δ*: 168.55, 154.72, 150.03, 140.51, 137.19, 132.08, 131.06, 129.54, 129.32, 129.10, 129.06, 128.86, 128.15, 127.10, 126.49, 125.87, 122.97, 122.34, 121.49, 121.37, 119.71, 118.76, 118.34, 115.98, 115.66, 50.15. HRMS (ESI) calcd for C_28_H_19_ClF_3_N_4_OS [M + H]^+^ 551.0920, found 551.0913, the error 1.27 ppm.

##### 2.1.2.7. 1-((Benzo[d]thiazol-2-ylamino)(pyrimidin-5-yl)methyl)naphthalen-2-ol (8 g)

White solid, yield 77%, R*_f_* 0.61 (dichloromethane/methanol 100:1), mp 216–219 °C. ^1^H NMR (400 MHz, DMSO-*d*_6_) *δ*: 10.08 (br, 1H), 9.03 (s, 1H), 8.94 (s, 1H), 8.64 (s, 2H), 7.99 (d, *J* = 7.4 Hz, 1H), 7.86 (d, *J* = 8.9 Hz, 2H), 7.71 (d, *J* = 7.8 Hz, 1H), 7.42–7.47 (m, 1H), 7.41 (d, *J* = 8.0 Hz, 1H), 7.28–7.35 (m, 2H), 7.20–7.26 (m, 2H), 7.03–7.07 (m, 1H); ^13 ^C NMR (101 MHz, DMSO-*d*_6_) *δ*: 165.6, 156.5, 154.6, 153.6, 151.7, 135.9, 131.9, 130.9, 130.3, 128.7, 128.4, 126.9, 125.5, 122.9, 122.7, 121.3, 121.0, 118.4, 116.6, 99.1, 50.1. HRMS (ESI) calcd for C_22_H_17_N_4_OS [M + H]^+^ 385.1123, found 385.1118, the error 1.30 ppm.

##### 2.1.2.8. 1-((Benzo[d]thiazol-2-ylamino)(3-bromo-1-(3-chloropyridin-2-yl)-1H-pyrazol-5-yl)methyl)naphthalen-2-ol (8 h)

White solid, yield 73%, R*_f_* 0.68 (petroleum ether/ethyl acetate 10:1), mp 163–165 °C. ^1^H NMR (400 MHz, CDCl_3_) *δ*: 10.85 (br, 1H), 8.18 (d, *J* = 4.0 Hz, 1H), 7.76 (d, *J* = 8.3 Hz, 1H), 7.65 (d, *J* = 8.0 Hz, 1H), 7.51–7.57 (m, 3H), 7.36–7.46 (m, 2H), 7.30–7.34 (m, 2H), 7.26–7.29 (m, 1H), 7.06–7.18 (m, 2H), 6.93–7.01 (m, 2H), 6.57 (s, 1H); ^13 ^C NMR (101 MHz, DMSO-*d*_6_) *δ*: 164.0, 163.8, 154.1, 148.0, 146.6, 139.0, 130.9, 128.9, 128.0, 126.8, 126.6, 125.1, 124.7, 123.2, 122.7, 122.1, 120.9, 119.7, 119.0, 115.7, 115.3, 112.2, 111.0, 110.6, 109.0, 50.1. HRMS (ESI) calcd for C_26_H_16_BrClN_5_OS [M-H]^-^ 561.9927, found 561.9924, the error 0.53 ppm.

##### 2.1.2.9. 1-((Benzo[d]thiazol-2-ylamino)(2-phenyl-2H-1,2,3-triazol-4-yl)methyl)naphthalen-2-ol (8i)

White solid, yield 91%, R*_f_* 0.73 (dichloromethane/methanol 100:1), mp 198–200 °C. ^1^H NMR (400 MHz, CDCl_3_) *δ*: 11.56 (br, 1H), 8.14 (d, *J* = 7.8 Hz, 2H), 7.75–7.85 (m, 2H), 7.67 (d, *J* = 8.1 Hz, 1H), 7.56–7.62 (m, 2H), 7.52–7.55 (m, 2H), 7.40–7.44 (m, 1H), 7.35–7.40 (m, 2H), 7.28–7.34 (m, 4H), 7.14–7.16 (m, 1H), 7.06 (s, 1H); ^13 ^C NMR (101 MHz, CDCl_3,_ DMSO-*d*_6_) *δ*: 167.2, 154.3, 150.6, 149.9, 139.7, 134.1, 132.0, 130.7, 130.0, 129.6, 129.2, 128.9, 127.4, 126.6, 126.1, 123.0, 122.9, 122.1, 121.0, 120.2, 118.7, 118.6, 117.2, 49.9. HRMS (ESI) calcd for C_26_H_18_N_5_OS [M-H]^-^ 448.1232, found 448.1235, the error 0.67 ppm.

##### 2.1.2.10. 1-((Benzo[d]thiazol-2-ylamino)(benzofuran-2-yl)methyl)naphthalen-2-ol (8j)

Yellow solid, yield 87%, R*_f_* 0.69 (dichloromethane/methanol 100:1), mp 166–168 °C. ^1^H NMR (400 MHz, DMSO-*d*_6_) *δ*: 10.35 (s, 1H), 9.07 (d, *J* = 5.6 Hz, 1H), 8.19 (d, *J* = 8.5 Hz, 1H), 7.85 (d, *J* = 8.9 Hz, 2H), 7.72 (d, *J* = 7.7 Hz, 1H), 7.53–7.59 (m, 1H), 7.51 (d, *J* = 4.6 Hz, 1H), 7.42–7.48 (m, 3H, Ph-H), 7.30–7.33 (m, 1H), 7.25 (d, *J* = 7.6 Hz, 1H), 7.17–7.22 (m, 2H), 7.05 (t, *J* = 7.5 Hz, 1H), 6.76 (s, 1H), 5.78 (s, 1H); ^13 ^C NMR (101 MHz, DMSO-*d*_6_) *δ*: 166.4, 158.7, 154.7, 154.2, 152.4, 133.0, 131.3, 130.6, 129.1, 129.0, 128.7, 127.0, 126.0, 124.1, 123.6, 123.2, 123.1, 121.7, 121.5, 121.3, 118.9, 118.8, 115.9, 111.4, 103.6, 55.4. HRMS (ESI) calcd for C_26_H_19_N_2_O_2_S [M + H]^+^ 423.1167, found 423.1153, the error 3.31 ppm.

##### 2.1.2.11. 1-(Benzo[b]thiophen-3-yl(benzo[d]thiazol-2-ylamino)methyl)naphthalen-2-ol (8k)

White solid, yield 86%, R*_f_* 0.66 (dichloromethane/methanol 100:1), mp 199–200 °C. ^1^H NMR (400 MHz, DMSO-*d*_6_) *δ*: 10.36 (s, 1H), 9.01 (d, *J* = 6.9 Hz, 1H), 8.23 (d, *J* = 8.5 Hz, 1H), 7.94 (d, *J* = 7.9 Hz, 1H), 7.80 (d, *J* = 8.7 Hz, 2H), 7.68 (d, *J* = 7.7 Hz, 1H), 7.62 (d, *J* = 8.0 Hz, 1H), 7.50 (d, *J* = 6.7 Hz, 2H), 7.37 (d, *J* = 7.8 Hz, 2H), 7.30 (t, *J* = 7.8 Hz, 2H), 7.22–7.27 (m, 2H), 7.19 (d, *J* = 7.4 Hz, 1H), 7.01–7.05 (m, 1H); ^13 ^C NMR (101 MHz, DMSO-*d*_6_) *δ*: 165.7, 153.2, 152.1, 140.2, 137.6, 136.4, 132.5, 130.7, 129.8, 128.6, 126.2, 125.4, 124.2, 124.0, 123.7, 123.5, 123.0, 122.4, 121.8, 121.0, 120.9, 118.4, 118.1, 116.9, 99.5, 50.1. HRMS (ESI) calcd for C_26_H_19_N_2_OS_2_ [M + H]^+^ 439.0939, found 439.0925, the error 3.19 ppm.

##### 2.1.2.12. 1-((Benzo[d]thiazol-2-ylamino)(6-chloroimidazo[1,2-a]pyridin-3-yl)methyl)naphthalen-2-ol (8 l)

Yellow solid, yield 75%, R*_f_* 0.13 (petroleum ether/ethyl acetate 3:2), mp 185–187 °C. ^1^H NMR (400 MHz, CDCl_3_) *δ*: 9.95 (s, 1H), 9.24 (s, 1H), 8.27 (s, 1H), 7.96 (d, *J* = 8.0 Hz, 1H), 7.84 (d, *J* = 8.0 Hz, 1H), 7.71–7.75 (m, 2H), 7.46–7.53 (m, 3H), 7.35–7.38 (m, 3H), 7.07–7.10 (m, 2H), 6.98 (s, 1H), 6.92 (s, 1H); ^13 ^C NMR (101 MHz, CDCl_3_) *δ*: 178.1, 152.0, 148.2, 146.8, 143.3, 130.8, 130.5, 130.3, 129.6, 129.3, 127.9, 127.3, 126.6, 126.3, 126.0, 125.0, 123.6, 123.0, 122.8, 122.1, 121.7, 120.9, 118.1, 116.2, 33.4. HRMS (ESI) calcd for C_25_H_16_ClN_4_OS [M-H]^-^ 455.0733, found 455.0735, the error 0.44 ppm.

##### 2.1.2.13. 1-((Benzo[d]thiazol-2-ylamino)(6-bromoimidazo[1,2-a]pyridin-3-yl)methyl)naphthalen-2-ol (8 m)

Yellow solid, yield 72%, R*_f_* 0.12 (petroleum ether/ethyl acetate 3:2), mp 186–188 °C. ^1^H NMR (400 MHz, CDCl_3_) *δ*: 9.95 (s, 2H), 9.68 (s, 2H), 8.31 (s, 2H), 7.81 (br, 1H), 7.61–7.70 (m, 5H), 7.44 (s, 1H), 7.35 (br, 1H), 7.17 (brs, 1H), 6.99 (s, 1H), 6.93 (s, 1H); ^13 ^C NMR (101 MHz, CDCl_3_) *δ*: 178.1, 159.4, 149.2, 147.7, 146.6, 143.5, 133.6, 130.3, 129.6, 129.3, 129.1, 128.8, 128.2, 127.4, 124.9, 124.4, 123.1, 121.9, 118.4, 116.6, 110.4, 107.9, 107.3, 100.0, 33.4. HRMS (ESI) calcd for C_25_H_16_BrN_4_OS [M-H]^-^ 499.0228, found 499.0232, the error 0.80 ppm.

##### 2.1.2.14. 1-((3-Bromo-1-(3-chloropyridin-2-yl)-1H-pyrazol-5-yl)((6-chlorobenzo[d]thiazol-2-yl)amino)methyl)naphthalen-2-ol (8n)

White solid, yield 59%, R*_f_* 0.65 (petroleum ether/ethyl acetate 10:1), mp 213–217 °C. ^1^H NMR (400 MHz, CDCl_3_, DMSO-d_6_) *δ*: 10.15 (br, 1H), 7.95 (s, 1H), 7.81 (d, *J* = 8.6 Hz, 1H), 7.41–7.48 (m, 1H), 7.33–7.38 (m, 1H), 7.20–7.32 (m, 5H), 7.13 (s, 1H), 6.96–7.09 (m, 2H), 6.68–6.88 (m, 2H), 6.42 (s, 1H); ^13 ^C NMR (101 MHz, CDCl_3_, DMSO-d_6_) *δ*: 154.9, 153.9, 147.9, 147.7, 146.6, 145.5, 138.7, 137.5, 137.3, 131.6, 131.6, 130.3, 128.3, 127.2, 126.6, 126.6, 126.0, 124.6, 122.9, 122.4, 120.2, 119.4, 119.1, 116.2, 109.0, 48.6. HRMS (ESI) calcd for C_26_H_17_BrCl_2_N_5_OS [M + H]^+^ 595.9714, found 595.9702, the error 2.01 ppm.

##### 2.1.2.15. 1-(((6-Chlorobenzo[d]thiazol-2-yl)amino)(2-phenyl-2H-1,2,3-triazol-4-yl)methyl)naphthalen-2-ol (8o)

White solid, yield 93%, R*_f_* 0.72 (dichloromethane/methanol 100:1), mp 194–197 °C. ^1^H NMR (400 MHz, CDCl_3_) *δ*: 11.24 (br, 1H), 8.12 (d, *J* = 7.9 Hz, 2H), 7.76–7.83 (m, 2H), 7.60 (s, 1H), 7.50–7.58 (m, 4H), 7.38–7.43 (m, 2H), 7.35 (d, *J* = 8.9 Hz, 1H), 7.32 (d, *J* = 2.1 Hz, 1H), 7.30 (d, *J* = 2.1 Hz, 1H), 7.29 (s, 2H), 7.02 (s, 1H); ^13 ^C NMR (101 MHz, CDCl_3_) *δ*: 174.9, 167.0, 154.6, 149.3, 148.7, 139.6, 134.0, 131.8, 131.3, 131.1, 130.2, 129.4, 129.0, 127.9, 127.7, 126.8, 126.5, 123.2, 123.1, 120.8, 119.5, 118.8, 100.0, 50.1. HRMS (ESI) calcd for C_26_H_19_ClN_5_OS [M + H]^+^ 484.0999, found 484.0985, the error 2.89 ppm.

##### 2.1.2.16. 1-((3-Bromo-1-(3-chloropyridin-2-yl)-1H-pyrazol-5-yl)((6-(trifluoromethyl)benzo[d]thiazol-2-yl)amino)methyl)naphthalen-2-ol (8p)

White solid, yield 60%, R*_f_* 0.64 (petroleum ether/ethyl acetate 10:1), mp 215–218 °C. ^1^H NMR (400 MHz, DMSO-*d*_6_) *δ*: 10.07 (s, 1H), 9.21 (d, *J* = 7.9 Hz, 1H), 8.13–8.16 (m, 2H), 7.92 (d, *J* = 8.5 Hz, 1H), 7.66 (t, *J* = 7.5 Hz, 2H), 7.52–7.63 (m, 3H), 7.46 (d, *J* = 7.5 Hz, 1H), 7.38 (t, *J* = 7.5 Hz, 1H), 7.22 (t, *J* = 7.2 Hz, 1H), 7.10 (s, 1H), 6.97 (d, *J* = 7.2 Hz, 1H), 6.59 (s, 1H); ^13 ^C NMR (101 MHz, DMSO-*d*_6_) *δ*: 168.2, 154.8, 153.8, 148.8, 146.9, 139.2, 131.6, 130.1, 128.3, 128.2, 127.7, 127.4, 126.4, 125.5, 124.0 (q, *J* = 286.84 Hz, CF_3_), 123.5, 122.5, 122.5, 122.2, 121.2, 118.7, 118.2, 117.9, 114.5, 108.6, 99.5, 47.3. HRMS (ESI) calcd for C_27_H_17_BrClF_3_N_5_OS [M + H]^+^ 629.9978, found 629.9973, the error 0.79 ppm.

##### 2.1.2.17. 1-((2-Phenyl-2H-1,2,3-triazol-4-yl)((6-(trifluoromethyl)benzo[d]thiazol-2-yl)amino)methyl)naphthalen-2-ol (8q)

White solid, yield 63%, R*_f_* 0.73 (dichloromethane/methanol 100:1), mp 176–180 °C. ^1^H NMR (400 MHz, CDCl_3_) *δ*: 11.02 (br, 1H), 8.10 (d, *J* = 7.8 Hz, 2H), 7.87 (s, 1H), 7.76–7.83 (m, 2H), 7.69 (d, *J* = 8.5 Hz, 2H), 7.59 (d, *J* = 8.4 Hz, 2H), 7.49–7.54 (m, 2H), 7.36–7.42 (m, 3H), 7.27–7.33 (m, 2H), 7.06 (s, 1H); ^13 ^C NMR (101 MHz, CDCl_3_) *δ*: 154.2, 152.5, 149.0, 139.6, 135.3, 135.3, 131.8, 131.4, 130.0, 129.4, 129.1, 127.7, 127.0, 126.8, 125.4, 124.3 (q, *J* = 272.70 Hz, CF_3_), 123.7, 123.7, 123.4, 118.8, 118.6, 116.6, 115.5, 113.8, 50.4. HRMS (ESI) calcd for C_27_H_19_F_3_N_5_OS [M + H]^+^ 518.1262, found 518.1254, the error 1.54 ppm.

### 2.2. Biological activity test

The larvicidal/acaricidal activities of the title compounds against oriental armyworm (*Mythimna separata* Walker), corn borer (*Ostrinia nubilalis*), diamondback moth (*Plutella xylostella* L.), mosquito (*Culex pipiens pallen*), bean aphid (*Aphis craccivora*), and spider mite (*Tetranychus cinnabarinus*) were investigated in relationship with the contrasts **viii**, **ix**, and chlorantraniliprole in a greenhouse in accordance with the published literature precedents^36–42^. Partial bioassay procedures can also be found in the supporting information. According to statistical requirements, each bioassay was repeated three times. Assessments were made on a dead-alive basis, and lethality rates were corrected using Abbott’s formula^43^. Evaluations were based on a percentage scale of 0–100%, where 0% equals no activity, and 100% equals total kill. The standard deviations of the tested biological values were within ±5%.

### 2.3. Calcium imaging experiments

Effects of **8 h**, **8i**, and **viii** on calcium ion channels in the central neurons isolated from the third-instar of *M. separata* were studied by calcium imaging techniques according to the references^34^^,^^44^. The detailed procedure is presented in the supporting information.

## 3. Results and discussion

### 3.1. Chemistry

**Scheme 1.**
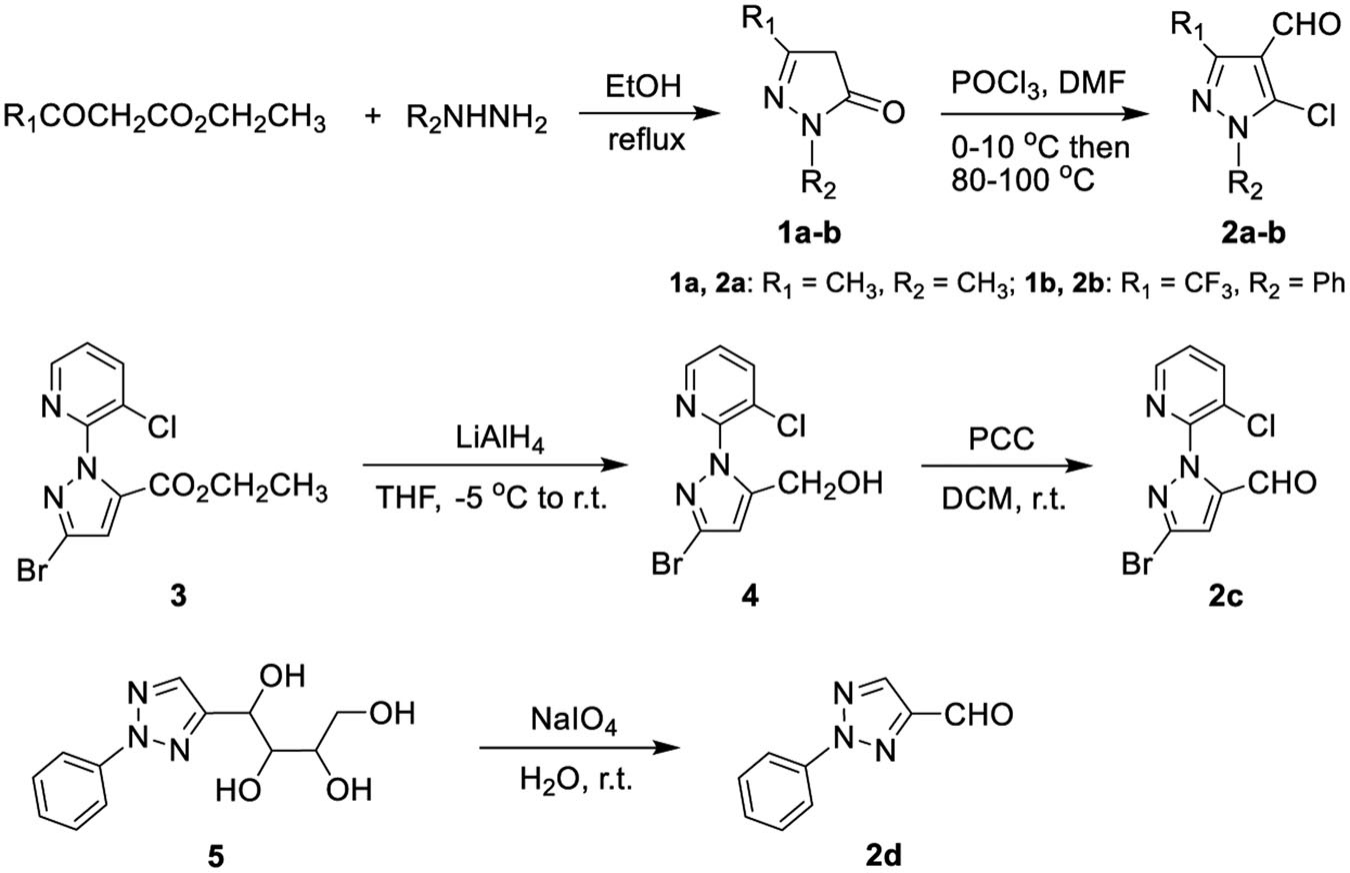
Synthetic routes for partial heteroaryl aldehyde intermediates **2a-d**.

As shown in Scheme 1, intermediate 5-chloro-1,3-disubstituted-1*H*-pyrazole-4-carbaldehydes (**2a-b**) were prepared *via* the cyclisation reaction of *β*-keto ester with hydrazine, and the subsequent POCl_3_-DMF Vilsmeier-Haack reaction of the generated pyrazolones (**1a-b**) with high yields following the known literature protocols^28–32^; using a similar procedure in the earlier report^34^, pyridylpyrazole carbaldehyde **2c** was prepared *via* LiAlH_4_-assisted reduction of pyrazole ester **3**, followed by subsequent PCC-mediated oxidation of the resultant pyrazole alcohol **4**; phenyltriazole carbaldehyde **2d** was smoothly synthesised from the intermediate triazolyl tetraol **5** using NaIO_4_-oxidation method^35^.

**Scheme 2.**
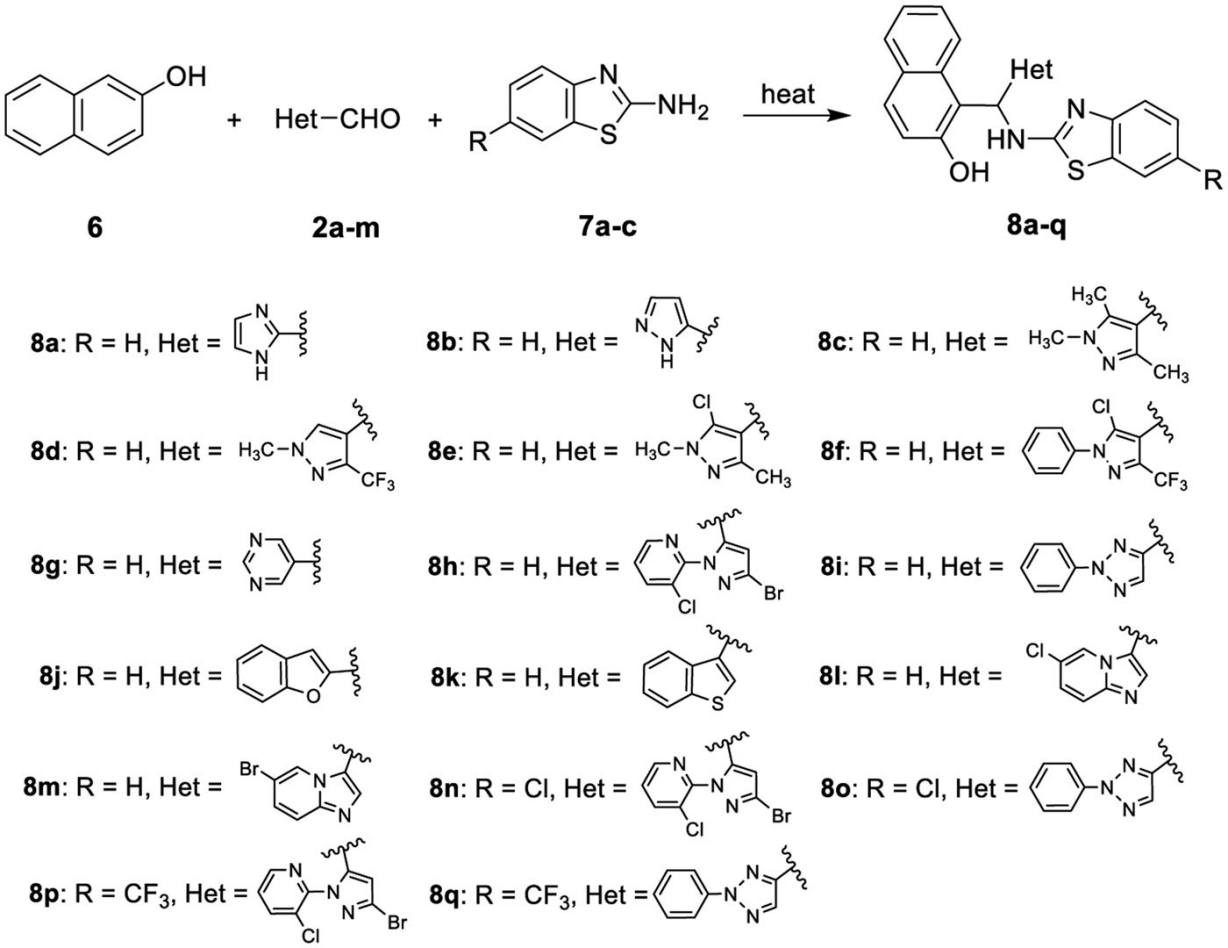
Synthetic route for the title compounds **8a-q**.

The synthetic route for the title compounds 1-(((6-substitutedbenzo[*d*]thiazol-2-yl)amino)(heteroaryl)methyl) naphthalen-2-ol (**8a-q**) is shown in Scheme 2. Using a one-pot pattern of the three component Betti reaction^45–47^, naphthalen-2-ol (**6**), heteroaryl aldehyde (**2**) and 6-substitutedbenzo[*d*]thiazol-2-amine (**7**) were reacted at 115–125 °C without any solvent for 1–2 h to give the title compounds **8a-q** in 57–93% yields. In the workup part of the reaction, the products were purified through either adding solvent-filtering-washing operation sequence, or direct column chromatography of the reaction mixture, indicating a simple, convenient and efficient procedure. Based on an Agilent 1260-II high performance liquid chromatography (HPLC) analysis, the representative compounds **8a**, **8d-8i**, **8 l**, **8o**, and **8q** were found to possess high purities of 90.11–100.00%.

**Scheme 3.**
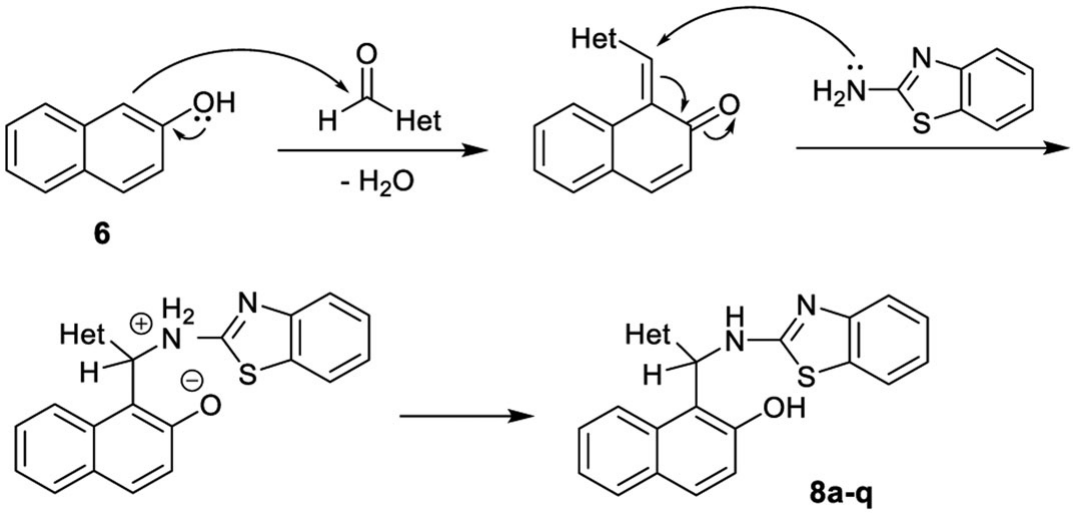
Possible mechanisms for the formation of the compounds **8a-q**.

The possible mechanism for this three-component reaction is presented in Scheme 3. In the second step of the process, the nucleophilic attack by the NH_2_ group of the reactant 2-aminobenzothiazole to the planar C=C double bond of *α*,*β*-unsaturated ketone should generate the corresponding intermediate containing a chiral carbon atom in the form of racemic mixture, due to the equal probability of the attack from both sides of the plane. Meanwhile, considering that the final compounds **8** were prepared under achiral condition, and taking into account their respective ^1^H NMR and ^13 ^C NMR spectra as well, the obtained products **8a-8q** should be considered as racemic mixtures.

The novel title compounds **8** were identified and characterised by means of ^1^H NMR, ^13 ^C NMR, HRMS and the melting point analyses. In their ^1^H NMR spectra, the chemical shifts of the –OH protons appeared at *δ* 9.95–12.44 ppm; the CH proton signals in the –CH–NH– part were observed at *δ* 5.59–7.11 ppm due to the presence of different heteroaryl substituents. In the ^13 ^C NMR spectra, the signals of carbon at 2- position (between N and S atoms) of the benzothiazole moiety, in majority of cases were found in the most high frequency area, at *δ* 165.6–178.1 ppm; the carbon signals in the –CH–NH– part were observed at *δ* 31.2–60.9 ppm, likewise, thanks to the various heteroaryl motifs.

### 3.2. Biological activities

The larvicidal/acaricidal activities of the title compounds against oriental armyworm (*Mythimna separata* Walker), corn borer (*Ostrinia nubilalis*), diamondback moth (*Plutella xylostella* L.), mosquito (*Culex pipiens pallen*), bean aphid (*Aphis craccivora*), and spider mite (*Tetranychus cinnabarinus*) were investigated in relationship with the contrasts **viii**, **ix**, and chlorantraniliprole in a greenhouse.

The insecticidal activities of the title compounds against larvae of oriental armyworm, corn borer, bean aphid, and mosquito are shown in Table 1. At a test concentration of 200 mg·L^−1^, most of the compounds were found to exhibit obvious larvicidal activities against oriental armyworm, among which **8a-8c**, **8f**, **8 h**-**8k**, **8 m**, **8o**, and **8p** possessed lethality rates of 50–100%, thus showing better insecticidal activities than the control **viii** (30%). Compounds **8a-8c**, **8 h**, and **8j** could still display favourable activities of 50–70% at a 100 mg·L^−1^ concentration, similar to that of the control **ix** (60%). Towards corn borer, most of the compounds also showed apparent larvicidal activities at a 200 mg·L^−1^ concentration with lethality rates of 25–65%, and were better than the control **viii** (10%), but less effective than **ix** (75%). Compounds **8 h**, **8i**, **8 m**, and **8p** exhibited insecticidal activities of 25–60% against bean aphid at a concentration of 200 mg·L^−1^, among which **8 h** and **8i** lethality rates of which were 40% and 60%, respectively, had similar insecticidal effects as for **ix** (50%). Surprisingly, the control **viii** showed no activity against bean aphid at this test concentration. The bioassay result for partial compounds (**8d**, **8 h**, **8i**, etc.) against mosquito indicated they have moderate to weak insecticidal activities (5–35%) at 10 mg·L^−1^ concentration, likewise with those of the two controls – **viii** and **ix**.

**Table 1.** Insecticidall activity (% lethality rate) of the title compounds against larvae of oriental armyworm (*Mythimna separata* Walker), corn borer (*Ostrinia nubilalis*), bean aphid (*Aphis craccivora*), and mosquito (*Culex pipiens pallens*) at different test concentrations.

Compd.	Oriental armyworm		Corn borer	Bean aphid	Mosquito
	200 mg·L^-1^	100 mg·L^-1^	200 mg·L^-1^	200 (100) mg·L^-1^	10 mg·L^-1^
**8a**	100	60	50	0	n.t.^a^
**8b**	100	50	50	20	n.t.
**8c**	100	50	40	0	n.t.
**8d**	10	n.t.	0	0	10
**8e**	40	n.t.	20	0	n.t.
**8f**	70	n.t.	35	15	n.t.
**8g**	10	n.t.	0	0	n.t.
**8h**	100	50	35	40	25
**8i**	100	20	10	60 (20)	20
**8j**	100	70	65	7	n.t.
**8k**	50	n.t.	10	0	n.t.
**8l**	30	n.t.	15	0	10
**8m**	50	n.t.	25	30	5
**8n**	0	n.t.	0	10	n.t.
**8o**	80	n.t.	40	20	n.t.
**8p**	50	n.t.	30	25	n.t.
**8q**	40	n.t.	25	0	n.t.
**viii**	30	n.t.	10	0	30
**ix**	100	60	75	50	5
chlorantraniliprole	100	100 (40%^b^)	100 (70%^c^)	90 (60)	100

^a^n.t.: not tested.

^b^at a concentration of 0.25 mg·L^−1^.

^c^at a concentration of 10 mg·L^−1^.

As shown in Table 2, most of the title compounds and the controls **viii** and **ix** displayed excellent insecticidal activities against diamondback moth larvae at the test concentrations of 200 mg·L^−1^ and 100 mg·L^−1^. At lower dosage, compounds **8a-8c**, **8f-8h**, **8j-8l**, **8n**, and **8o** had 50–95% insecticidal activities at a concentration of 10 mg·L^−1^, even higher than those of above two controls; on top of this, **8 b**, **8f**, **8 g**, **8j**, **8k**, **8n**, and **8o** also possessed lethality rates of 65–88% at a concentration of 1 mg·L^−1^, indicating that these compounds have better insecticidal effect than those of the two controls towards diamondback moth.

**Table 2.** Insecticidal activity (% lethality rate) against larvae of diamondback moth (*Plutella xylostella* L.) and acaricidal activity against adult spider mite (*Tetranychus cinnabarinus*) of the title compounds at different test concentrations.

Compd.	Diamondback moth				Spider mite	
	200 mg·L^−1^	100 mg·L^−1^	10 mg·L^−1^	1 mg·L^−1^	200 mg·L^−1^	100 mg·L^−1^
**8a**	100	80	65	n.t.^a^	0	n.t.
**8b**	100	94	85	70	0	n.t.
**8c**	100	100	75	35	0	n.t.
**8d**	80	40	0	n.t.	0	0
**8e**	75	45	n.t.	n.t.	0	n.t.
**8f**	100	100	92	77	0	n.t.
**8g**	100	95	80	65	0	n.t.
**8h**	100	80	50	n.t.	50	0
**8i**	100	75	40	n.t.	100	90
**8j**	100	100	90	85	n.t.	n.t.
**8k**	100	100	90	80	0	n.t.
**8l**	100	90	50	n.t.	60	30
**8m**	100	70	30	n.t.	80	40
**8n**	100	100	95	88	30	n.t.
**8o**	100	100	90	74	0	n.t.
**8p**	80	65	n.t.	n.t.	0	n.t.
**8q**	67	40	n.t.	n.t.	0	n.t.
**viii**	100	90	60	n.t.	90	60
**ix**	100	90	70	n.t.	70	30
chlorantraniliprole	100	100	100	87 ^b^	40	0

^a^n.t.: not tested.

^b^at a concentration of 0.001 mg·L^−1^.

Moreover, the acaricidal activity results of the compounds against adult spider mite are also listed in Table 2. As shown in the table, some of the compounds exhibited good acaricidal activities at 200 mg·L^−1^ concentration, such as **8 h** − 50%, **8i** − 100%, **8 l** − 60%, **8 m** − 80%, and were comparable with the controls **viii** (90%) and **ix** (70%). At a concentration of 100 mg·L^−1^, compounds **8 l** and **8 m** lethality rates of which were 30% and 40%, respectively, had the similar acaricidal levels as for **ix** (30%), while being less effective than **viii** (60%). It is worth noting that compound **8i** with 90% activity at 100 mg·L^−1^ concentration is superior to all the controls. Interestingly, the control chlorantraniliprole which exhibited excellent and the best insecticidal activity in all these biological tests, just showed 40% and 0% acaricidal activities against spider mite at 200 mg·L^−1^ and 100 mg·L^−1^ concentrations, respectively.

### 3.3. Structure-activity relationships

Based on the preliminary biological assay results, the following structure-activity relationships could be found:

Generally, when R = H, the compound bearing benzofuryl group (**8j**) displayed remarkable insecticidal activities. The other heterocyclic substituents (Het), such as imidazolyl, (substituted)pyrazolyl, (Ph)triazolyl and benzothienyl groups also exhibited different degrees of favourable insecticidal potentials. Moreover, when R = H, the Het group in corresponding compounds against spider mite showed an acaricidal activity trend: (Ph)triazolyl > (Br)imidazopyridyl > (Cl)imidazopyridyl > (Br)(2-ClPy)pyrazolyl (i.e. in Table 2, **8i** > **8 m** > **8 l** > **8 h**). Among these compounds, **8i** with a heterocyclic substituent being a (Ph)triazolyl group possessed the best acaricidal potential. These results indicate that the smaller and less electronegative groups would be more favourable for the increase of acaricidal activities of the compounds.

When R = Cl and CF_3_, respectively, the Het group in corresponding compounds against both oriental armyworm and corn borer showed the activity sequence of (Ph)triazolyl ≫ (Br)(2-ClPy)pyrazolyl (i.e. **8o** ≫ **8n**), and (Ph)triazolyl ≈ (Br)(2-ClPy)pyrazolyl (i.e. **8q** ≈ **8p**), respectively, indicating the similar positive influence of the smaller and less electronegative Het groups on the insecticidal activities; when R = Cl or CF_3_, the Het group in the related compounds against diamondback moth showed an opposite activity trend of (Br)(2-ClPy)pyrazolyl > (Ph)triazolyl (i.e. **8n** > **8o**; **8p** > **8q**). When the Het group is fixed as (Br)(2-ClPy)pyrazolyl or (Ph)triazolyl, the activity trend for R group is Cl > H > CF_3_ (i.e. **8n** > **8 h** > **8p**; **8o** > **8i** > **8q**) in diamondback moth tests.

When the group R in benzothiazole ring is H, the heterocycle substituents (Het) such as imidazole, pyridine, pyrimidine and simply-substituted pyrazole moiety are beneficial for the maintenance of the insecticidal activities towards diamondback moth, e.g., in the case of compounds **8a-8c** and **8 g**. In particular, pyrazole ring bearing three electron-donating Me groups (i.e., **8c**) greatly contributed to the activity improvement compared to those bearing electron-withdrawing groups CF_3_ (i.e., **8d**) and Cl (i.e., **8e**), indicating that groups with stronger electronegativity in pyrazole ring generally was unfavourable. Furthermore, the fused-heterocycle substituents such as benzofuran and benzothiophene groups could lead to high insecticidal activities of the related compounds. While the former exhibited a better trend for the activity enhancement than that of the latter, hence indicating that the smaller and more electronegative groups would be favourable. Bi-(hetero)aryl substituents also showed similar trend, that is, the activity sequence corresponding to the Het groups being as follows: (Ph)(Cl,CF_3_)pyrazolyl (**8f**) > (Br)(2-ClPy)pyrazolyl (**8 h**) > (Ph)triazolyl (**8i**).

In addition, OH and NH groups present in the skeleton of this type of compounds can both serve as hydrogen bond donors as well as acceptors. Besides this, there are also one or more O/N atoms in the Het substituents that could be hydrogen bond acceptors. Whereas according to the preliminary bioassay results, it seems that the type and number of these hydrogen bond acceptors do not have much influence on the insecticidal activities of the corresponding compounds.

Taking it by and large, these compounds towards oriental armyworm showed a consistent activity trend against diamondback moth. Interestingly, there are a few exceptions, e.g., compounds **8n** and **8 g** which possessed very high insecticidal activities towards diamondback moth, while showing rather weak activities in oriental armyworm tests at 200 mg·L^−1^ concentration. This may be due to the different selectivity of the compounds to different insects, *Mythimna separata* Walker and *Plutella xylostella* L.

Compounds **8 b**, **8f**, **8 g**, **8j**, **8k**, **8n**, and **8o** with favourable insecticidal potentials were also determined the LC_50_ values against diamondback moth (*Plutella xylostella* L.) with compounds **ix** and chlorantraniliprole as controls. As shown in Table 3, the tested compounds held LC_50_ values of 0.0988–5.8864 mg·L^−1^ and were all superior to the control **ix** (LC_50_: 13.5493 mg·L^−1^) towards diamondback moth.

**Table 3.** LC_50_ values of the compounds against diamondback moth (*Plutella xylostella* L.)^a^

Compd.	*y* = a + b*x*	LC_50_ (mg·L^-1^)	*R*
**8b**	*y* = 4.5471 + 1.0361*x*	2.7359	0.9982
**8f**	*y* = 4.6346 + 1.0784*x*	2.1821	0.9965
**8g**	*y* = 5.1328 + 0.7661*x*	0.6710	0.9374
**8j**	*y* = 4.2828 + 1.1704*x*	4.1003	0.9986
**8k**	*y* = 4.0871 + 1.1858*x*	5.8864	0.9990
**8n**	*y* = 3.9003 + 1.4515*x*	5.7228	0.9971
**8o**	*y* = 5.6404 + 0.6370*x*	0.0988	0.9471
**ix**	*y* = 3.4459 + 1.3730*x*	13.5493	0.9651
chlorantraniliprole	*y* = 8.3144 + 1.0316*x*	0.0006	0.9962

^a^The bioassays were carried out at a different period time than those in Table 2, however in the same batch.

In general, compared with the phenyl groups in the structures of the controls **viii** and **ix**, some of the heterocyclic groups in these synthesised compounds showed favourable potential for the enhancement of the insecticidal and acaricidal activities of the parent structures. Compounds **8 b**, **8c**, **8f**, **8 g**, **8 h**, **8i**, **8j**, **8k**, **8n**, and **8o** could be considered as new insecticidal leading structures, meanwhile compounds **8i**, **8 l**, and **8 m** could also be viewed as novel acaricidal leading structures for further investigations.

### 3.4. The effects of β-naphthol derivatives on the insect calcium ion channels

**Figure 2.**
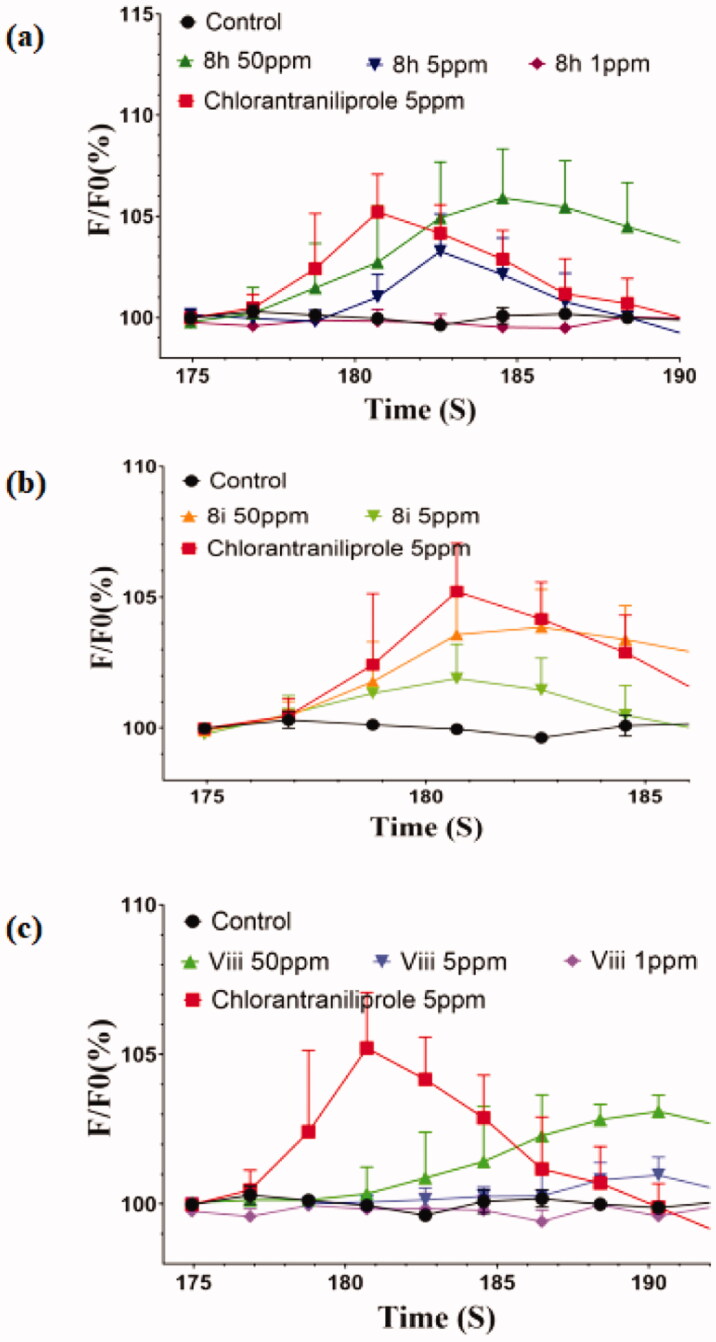
Effects of compounds **8 h (a)**, **8i (b)**, and **viii (c)** at different concentrations on [Ca^2+^]_i_ on the central neurons of *M. separata* when extracellular calcium was absent (The central neurons of third-instar larvae of *M. separata* were dyed with fluo-3 AM).

Taking into account the promising bioactivities of these synthesised novel *β*-naphthol derivatives, the effects on the calcium ion channels of the representative compounds **8 h**, **8i**, and **viii** with high bioactivities were further tested. The experiments were performed by using the calcium imaging techniques^34^^,^^44^ on central neurons isolated from the third-instar of oriental armyworm (*M. separata*) after neurons being loaded with fluo-3 AM (The detailed procedure was presented in the supporting information). Figure 2 shows the free calcium ion change in neuron cytoplasm versus recorded time when the central neurons were treated with each of **8 h**, **8i**, **viii**, and chlorantraniliprole in the absence of extracellular calcium ions.

According to the information shown in Figure 2, the following results could be found: when treating neurons with each of compounds **8 h**, **8i**, and **viii** at a low concentration of 1 mg·L^−1^, no significant increase in [Ca^2+^]_i_ was noticed; however, when being treated with each of these three compounds and chlorantraniliprole at 5 mg·L^−1^ concentration, increase of about 3.27% for **8 h** (103.27 ± 1.85%, *n* = 12), 1.90% for **8i** (101.90 ± 1.31%, *n* = 8), and 0.96% for **viii** (100.96 ± 0.60%, *n* = 7) in [Ca^2+^]_i_ was detected, respectively, which in turn is lower than that of the chlorantraniliprole (5 mg·L^−1^, 105.22 ± 1.86%, *n* = 4); when treating the neurons with each of the three compounds (**8 h**, **8i**, and **viii**) at a higher concentration of 50 mg·L^−1^, the increase of [Ca^2+^]_i_ caused by these compounds was more notable, that is, there was an increase of about 5.92% of [Ca^2+^]_i_ for **8 h** (105.92 ± 2.42%, *n* = 10), 3.85% for **8i** (103.85 ± 1.44%, *n* = 9), and 3.09% for **viii** (103.09 ± 0.54%, *n* = 5). These results indicate that the three compounds could activate the release of calcium ions in insect central neurons at a comparatively higher concentration.

Furthermore, when the neurons were preincubated with 0.5 µM ryanodine (a potent RyR activator) for 5 min, the free calcium ions were found to be elevated about 1.56% after treating with 5 mg·L^−1^ of **8i** (101.56 ± 1.00, *n* = 5), signifying that the calcium ions released from the endoplasmic reticulum increased to a certain degree compared to those of unincubated with ryanodine^44^^,^^48^. However, there were no significant changes for free calcium ion concentration in the cases of **8 h** (5 mg·L^−1^, 100.63 ± 0.62, *n* = 3) and **viii** (5 mg·L^−1^, 100.38 ± 0.54, *n* = 3). Based on the stress time, the increase of the calcium signal triggered by these novel compounds, is more likely to be a stress response, and probably not derived from the activation of RyR as in case of treatment with chlorantraniliprole.

Compared with chlorantraniliprole, all three compounds overall exhibited a modest effect on activating the release of calcium ions in insect central neurons. Besides the possible reason of being weak RyR modulators, very high liposolubility of these compounds as seen in CLogP values predicted by the ChemDraw Professional software (6.468 for **8 h**, 5.977 for **8i**, 5.799 for **viii**, and 3.194 for chlorantraniprole) could be another key factor determining the observed outcome. Among the three compounds, **8 h** showed better effect on activating the release of calcium ions at higher test concentrations than those of **8i** and **viii**, may be due to the presence of more electronegative and bulkier groups in the heterocyclic moieties (Br, Cl Vs. H, and OMe; (Py)pyrazole ring Vs. (Ph)triazole ring, and benzene ring).

## 4. Conclusions

In summary, a series of novel *β*-naphthol derivatives containing benzothiazolylamino and heteroaryl groups **8a-q** were efficiently synthesised *via* a one-pot pattern of the three component Betti reaction, and their structures were identified and characterised by melting points, ^1^H NMR, ^13 ^C NMR and HRMS analyses. The insecticidal and acaricidal activities of these new compounds towards a variety of agricultural pests were evaluated. The bioassay results showed that most of the title compounds possessed favourable insecticidal activities particularly towards oriental armyworm (50–100% at 200 mg·L^−1^) and diamondback moth (50–95% at 10 mg·L^−1^). Compounds **8 b**, **8f**, **8 g**, **8j**, **8k**, **8n**, and **8o** possessed LC_50_ values of 0.0988–5.8864 mg·L^−1^ against diamondback moth. Moreover, compounds **8 h**, **8i**, **8 l**, and **8 m** held lethality rates of 50–100% against spider mite at 200 mg·L^−1^ concentration, especially **8i**, **8 l**, and **8 m** featuring activities of 30–90% at 100 mg·L^−1^ concentration which were comparable with those of the controls. On the whole, compounds **8 b**, **8c**, **8f**, **8 g**, **8 h**, **8i**, **8j**, **8k**, **8n**, and **8o** could be viewed as new insecticidal leading structures, and on top of that, compounds **8i**, **8 l**, and **8 m** could also serve as novel acaricidal leading structures for further investigations. The calcium imaging experiments revealed that **8 h**, **8i**, and **viii** could activate the release of calcium ions in insect central neurons at a higher concentration (50 mg·L^−1^). It was speculated that this change is more likely to be a stress response in the present study. In view of the promising application potentials of phenolic and heterocyclic compounds in medicinal and pesticidal chemistry, more novel *β*-naphthol compounds could hopefully be developed with potent insecticidal/acaricidal activities and low toxicities with the further optimisation of the leading structures in this research. These studies will give the significant input for the design and development of new generation of insecticidal/acaricidal agrochemicals.

## Supplementary Material

Supplemental Material
