# Supplementary material for: Synthesis and evaluation of novel 1-(((6-substitutedbenzo[d]thiazol-2-yl)amino)(heteroaryl)methyl)naphthalen-2-ol as pesticidal agents
Source: J Enzyme Inhib Med Chem. 2022 Jan 27;37(1):641–51. doi: 10.1080/14756366.2022.2032687 (PMC8797731; doi:10.1080/14756366.2022.2032687)
Supplement: Supplemental Material [file IENZ_A_2032687_SM8281.pdf]

Supporting Information

Synthesis and evaluation of novel 1-(((6-substitutedbenzo[d]thiazol-2-yl)amino)(heteroaryl)methyl)naphthalen-2-ol as pesticidal agents

Junfeng Shang [a], Yuxin Li [a,b], Na Yang [a], Lixia Xiong [a] and Baolei Wang [a]

[a] *State Key Laboratory of Elemento-Organic Chemistry, College of Chemistry, Nankai University, Tianjin 300071, China*

[b] *Key Laboratory of Study and Discovery of Small Targeted Molecules of Hunan Province, School of Medicine, Hunan Normal University, Changsha 410125, China*

CONTACT Baolei Wang, E-mail address: nkwbl@nankai.edu.cn; correspondence address: State Key Laboratory of Elemento-Organic Chemistry, College of Chemistry, Nankai University, Tianjin 300071, China

---

1. The ^{1}H NMR spectra of the intermediates S2

2. The ^{1}H and ^{13}C NMR spectra of the title compounds **8a-q** S6

3. The HRMS spectra of the title compounds **8a-q** S23

4. Biological activity test S31

5. Calcium imaging ~~experiment~~ experiments S33

6. References S34

1. The ^{1}H NMR spectra of the intermediates

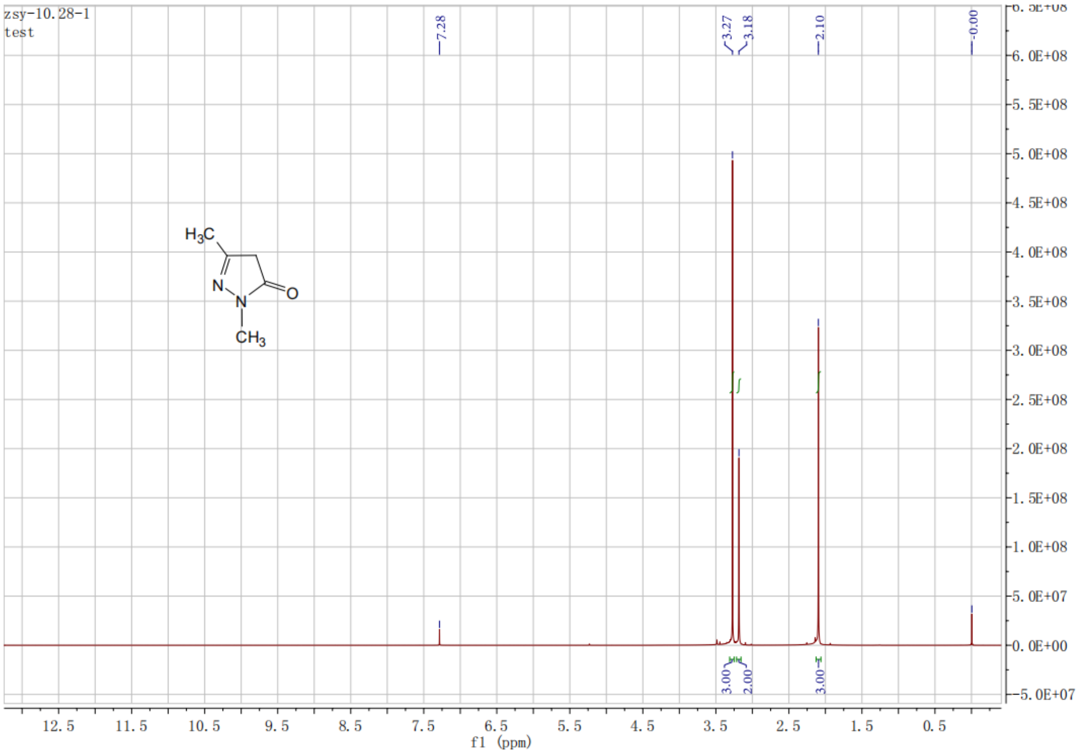

Figure S1. The ^{1}H NMR spectrum of intermediate **1a** (400 MHz, $CDCl_3$).

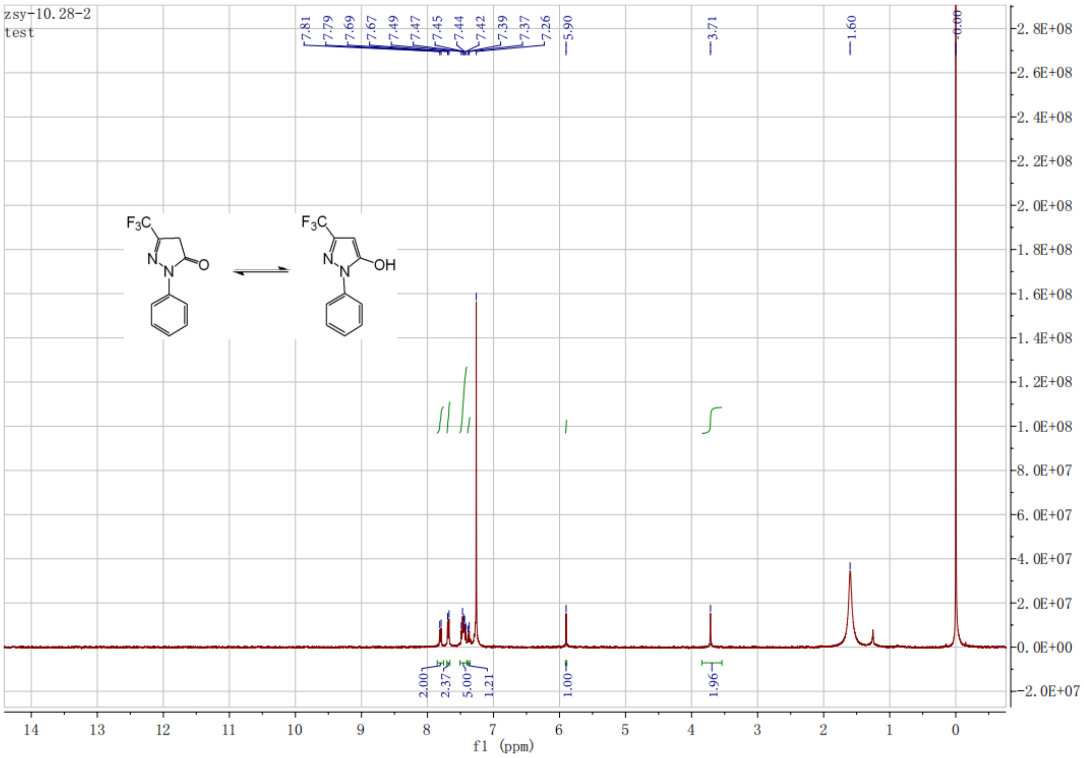

Figure S2. The ^{1}H NMR spectrum of intermediate **1b** (400 MHz, $CDCl_3$).

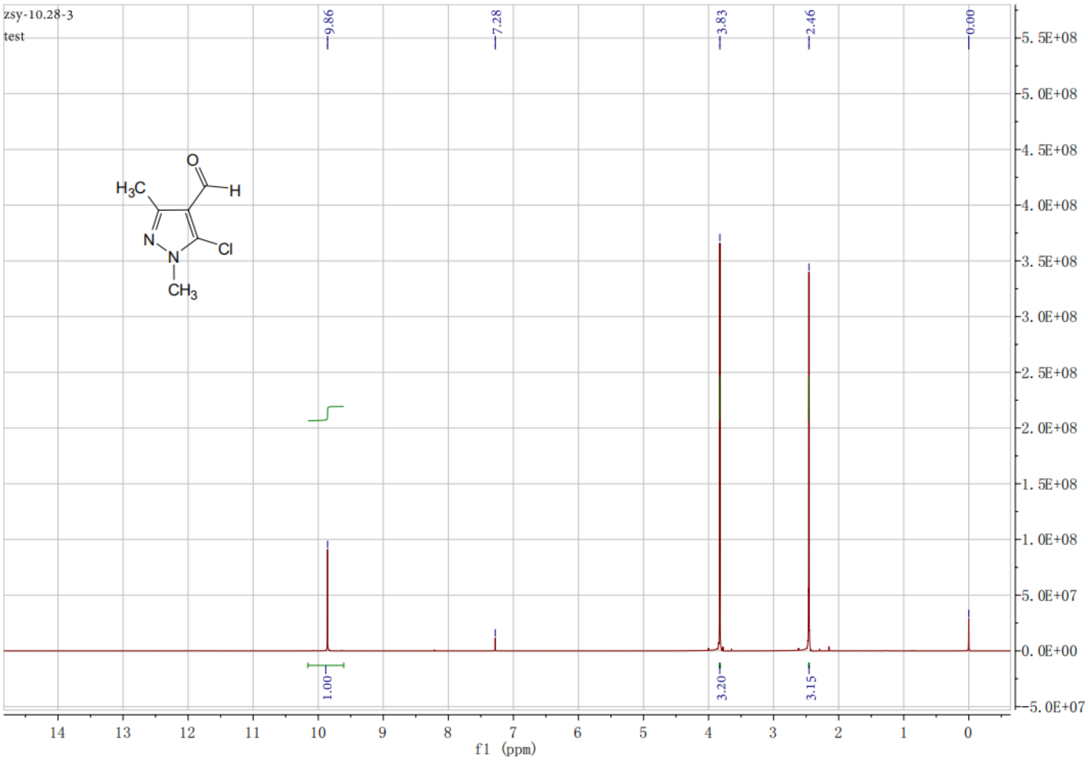

Figure S3. The ^{1}H NMR spectrum of intermediate **2a** (400 MHz, $CDCl_3$).

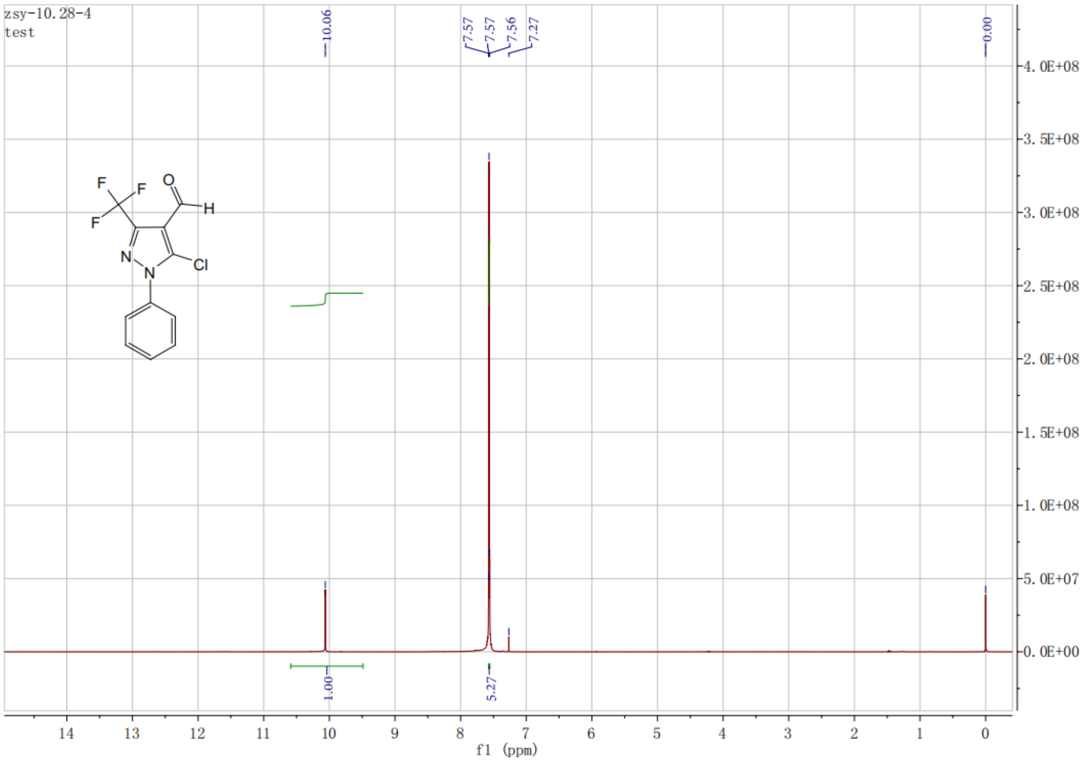

Figure S4. The ^{1}H NMR spectrum of intermediate **2b** (400 MHz, $CDCl_3$).

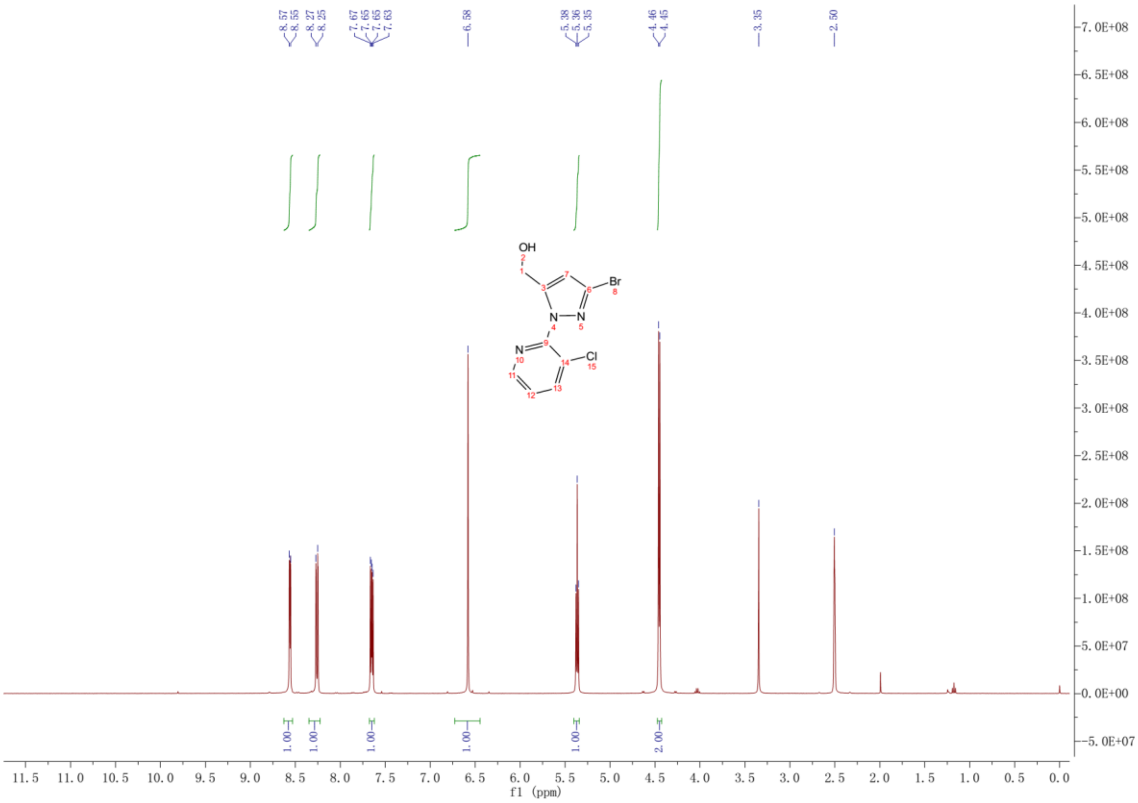

Figure S5. The ^{1}H NMR spectrum of intermediate **4** (400 MHz, DMSO-d_6).

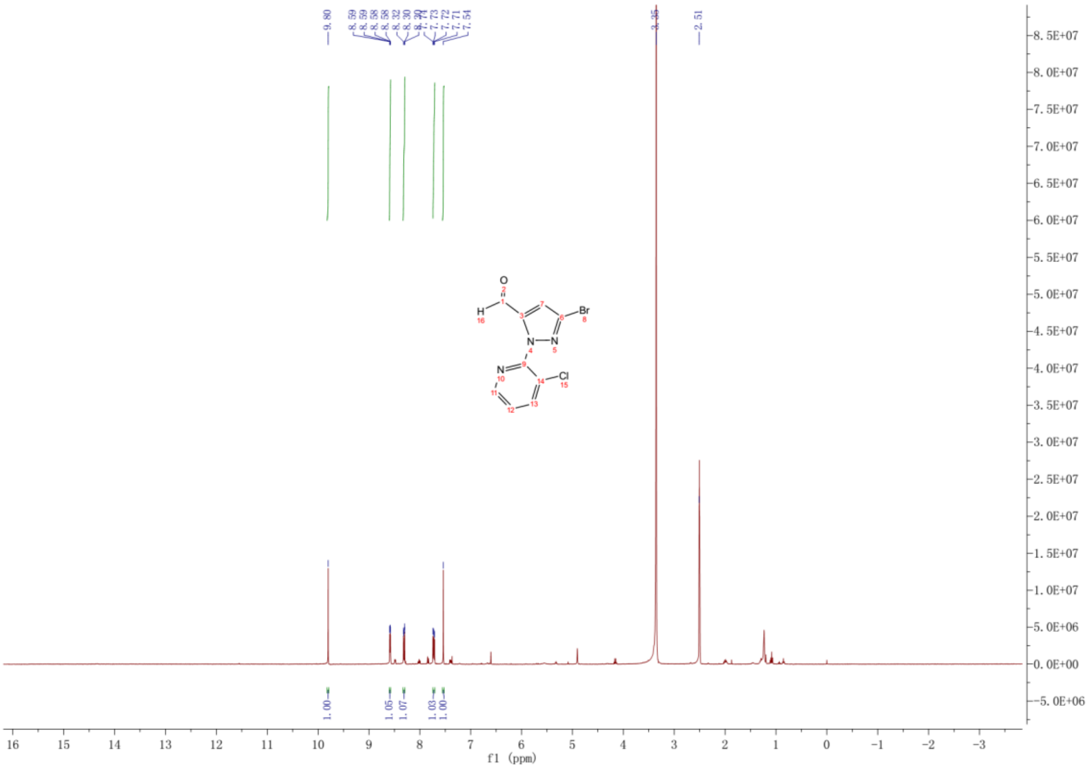

Figure S6. The ^{1}H NMR spectrum of intermediate **2c** (400 MHz, DMSO-d_6).

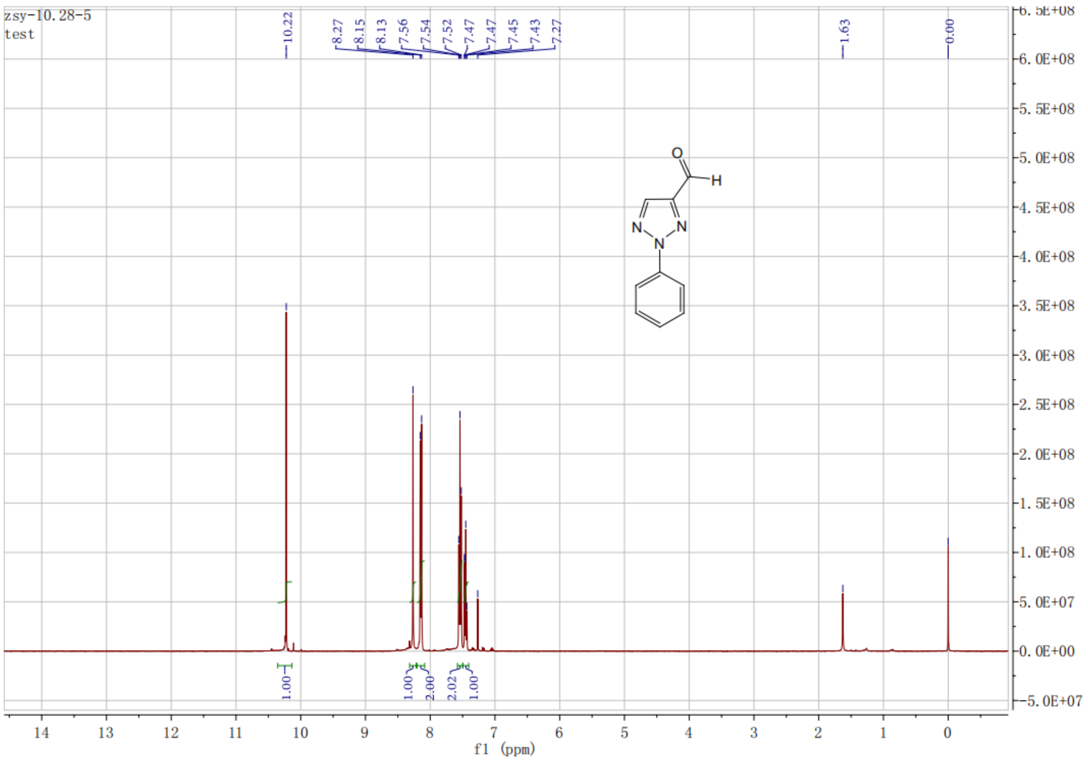

Figure S7. The ^{1}H NMR spectrum of intermediate **2d** (400 MHz, $CDCl_3$).

2. The ^{1}H and ^{13}C NMR spectra of the title compounds 8a-q

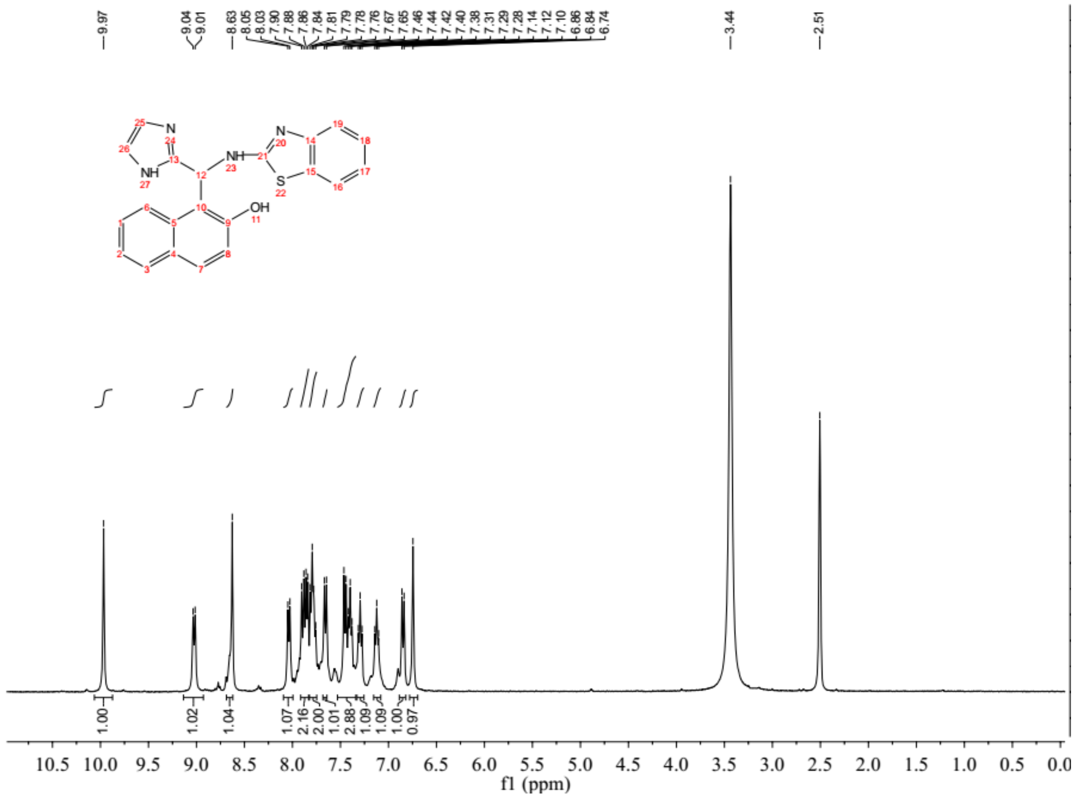

Figure S8. The ^{1}H NMR spectrum of compound **8a** (400 MHz, DMSO-d_6).

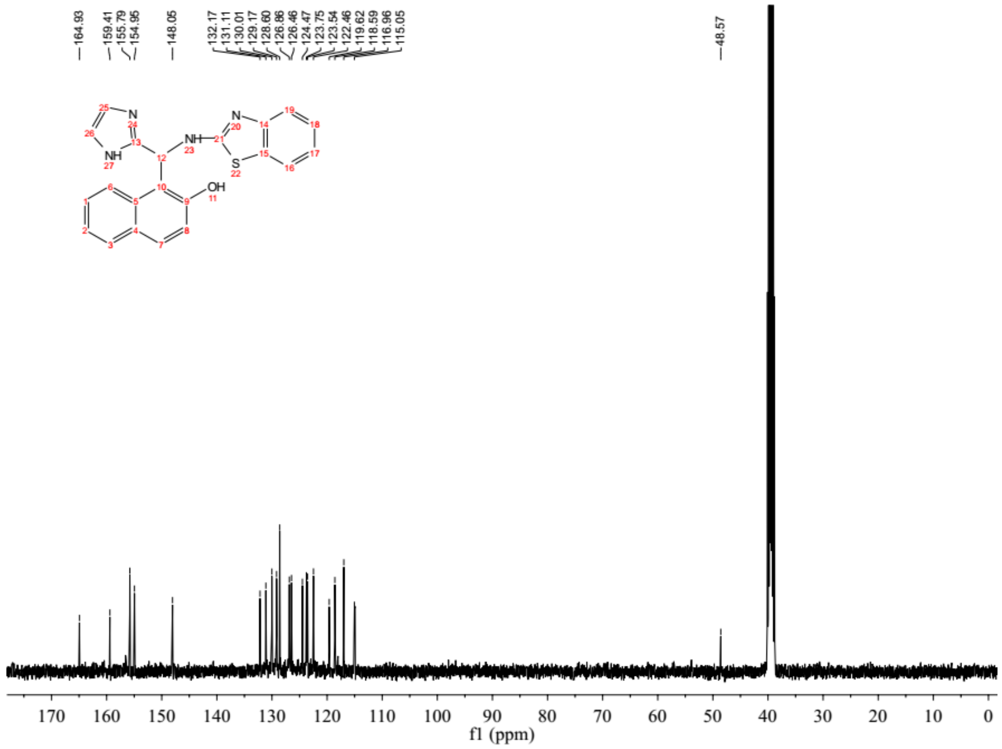

Figure S9. The ^{13}C NMR spectrum of compound **8a** (101 MHz, DMSO-d_6).

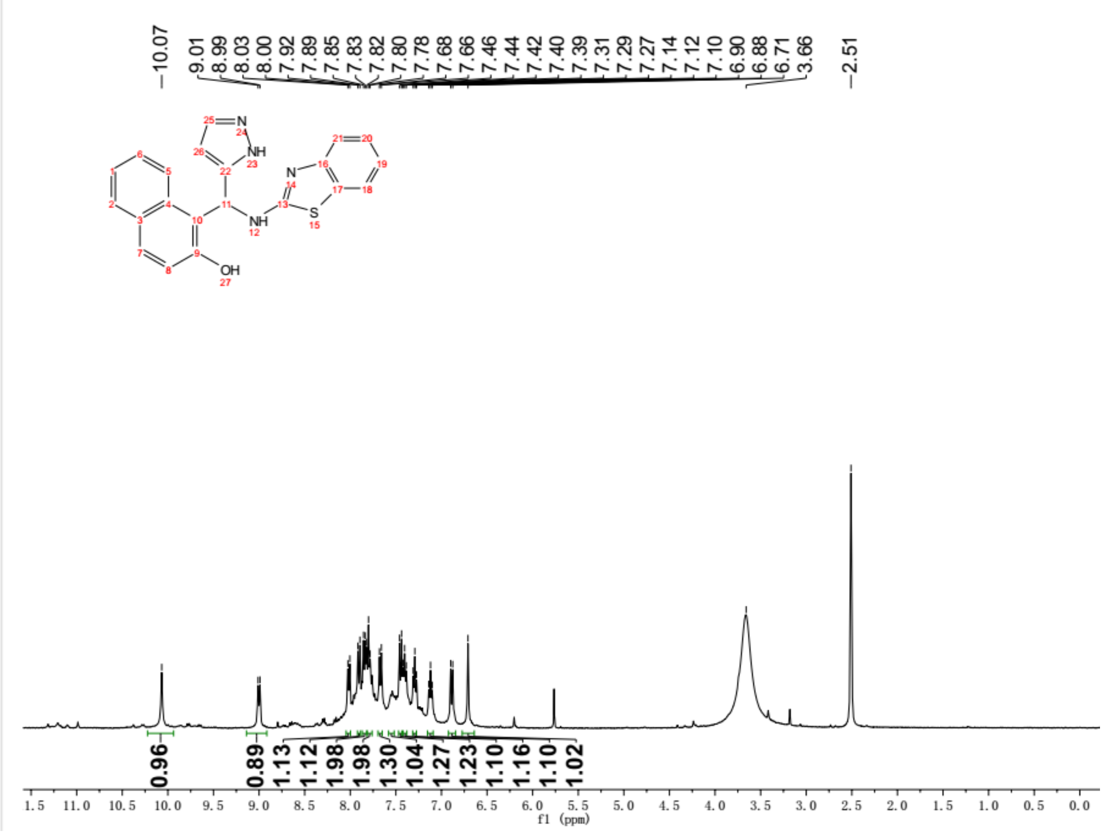

Figure S10. The ^{1}H NMR spectrum of compound **8b** (400 MHz, DMSO-d_6).

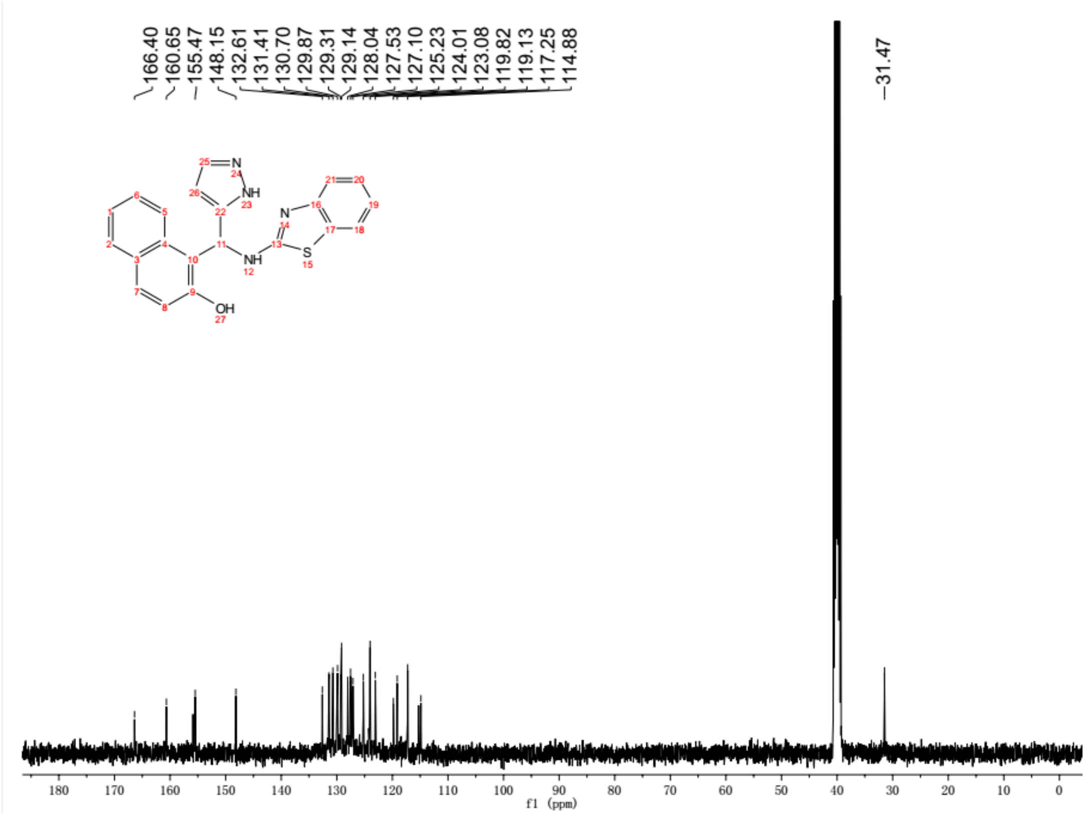

Figure S11. The ^{13}C NMR spectrum of compound **8b** (101 MHz, DMSO-d_6).

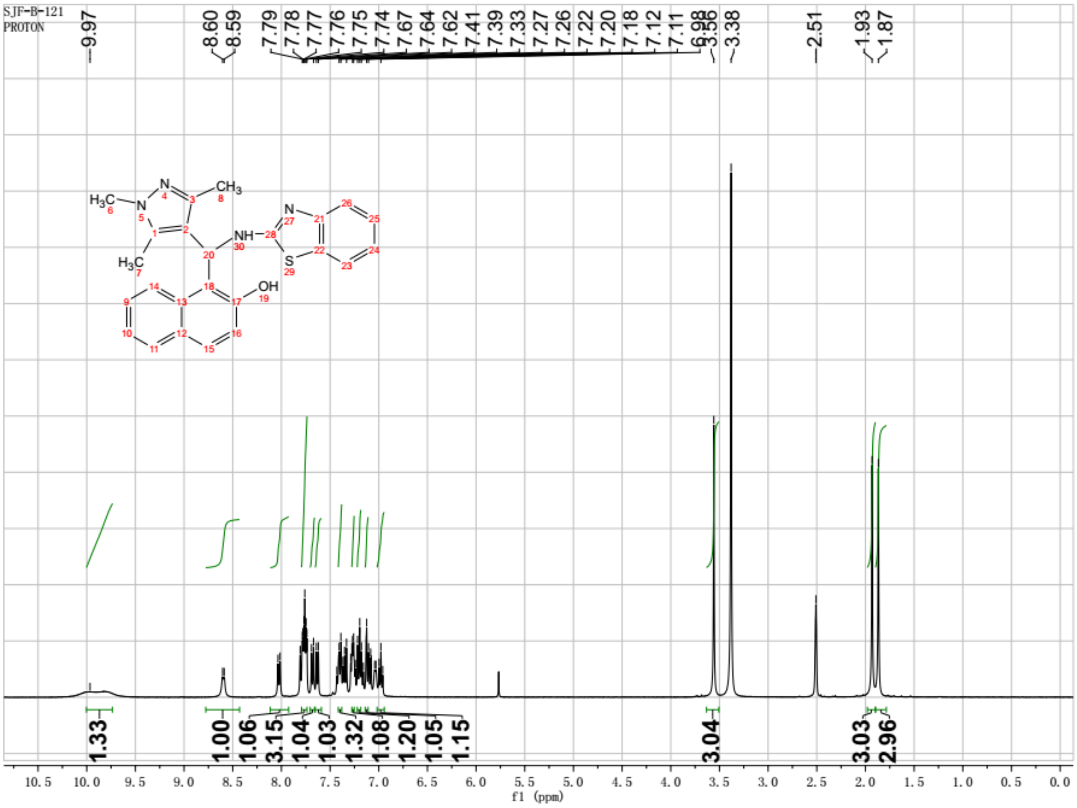

Figure S12. The ^{1}H NMR spectrum of compound **8c** (400 MHz, DMSO-d_6).

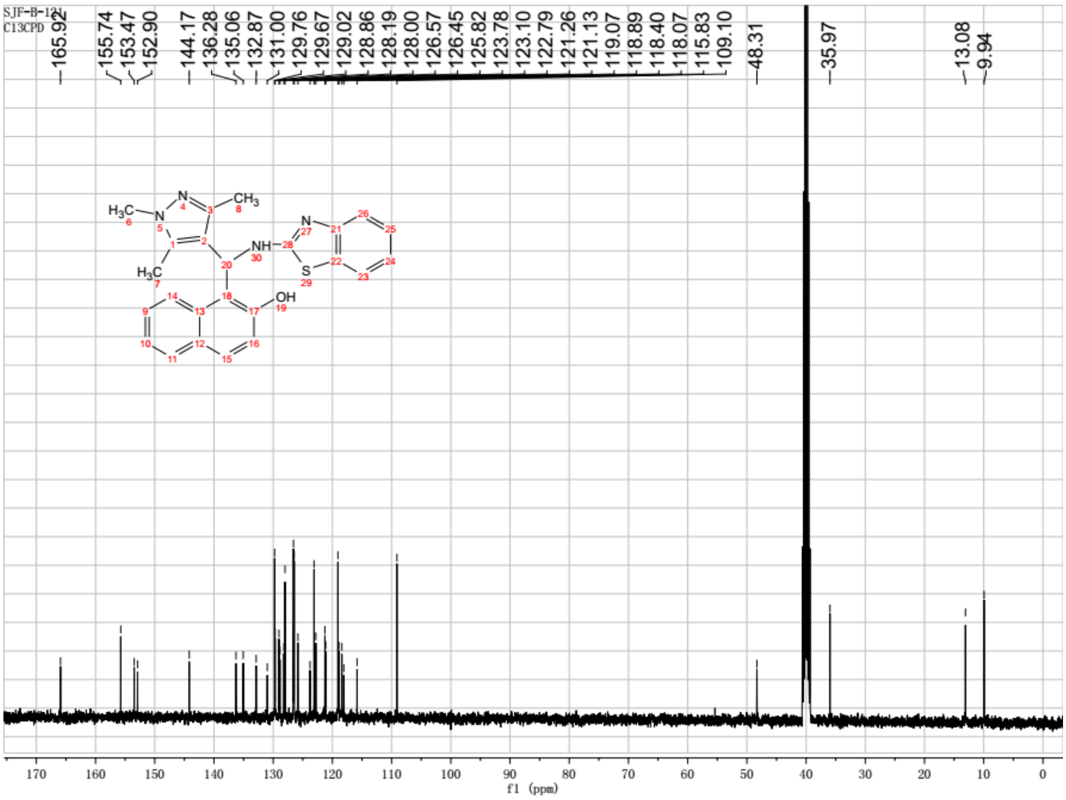

Figure S13. The ^{13}C NMR spectrum of compound **8c** (101 MHz, DMSO-d_6).

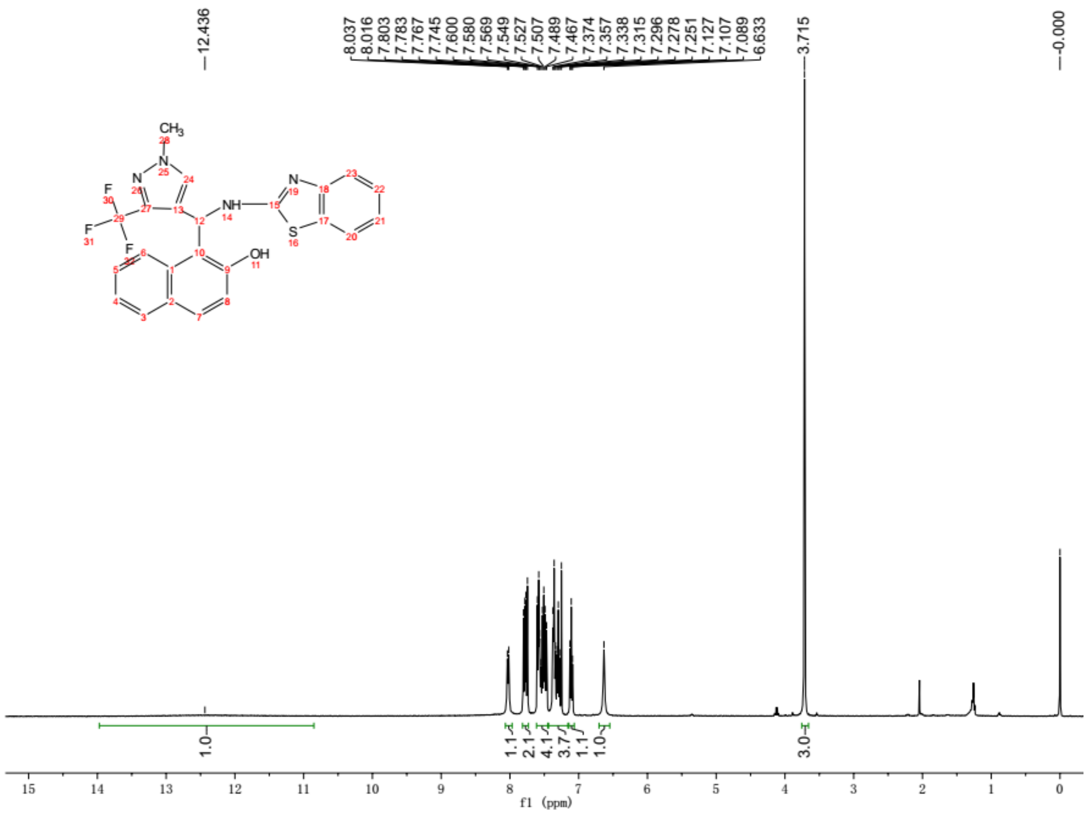

Figure S14. The ^{1}H NMR spectrum of compound **8d** (400 MHz, $CDCl_3$).

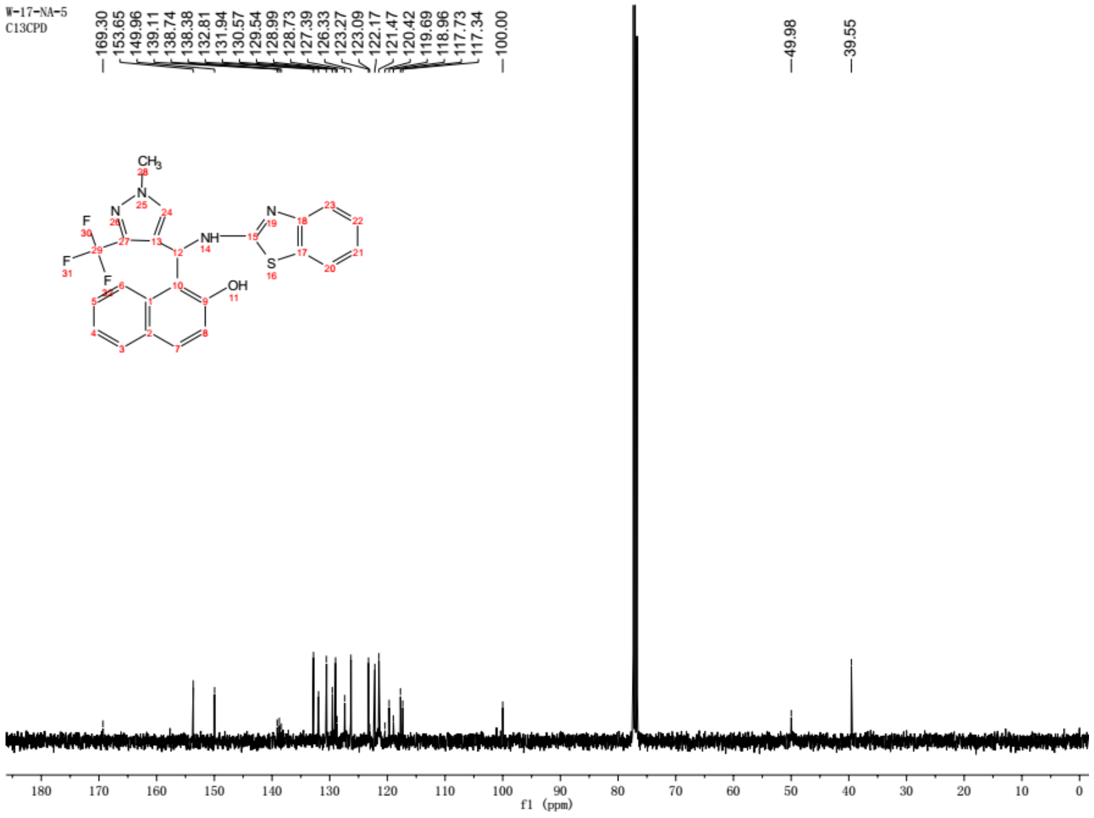

Figure S15. The ^{13}C NMR spectrum of compound **8d** (101 MHz, $CDCl_3$).

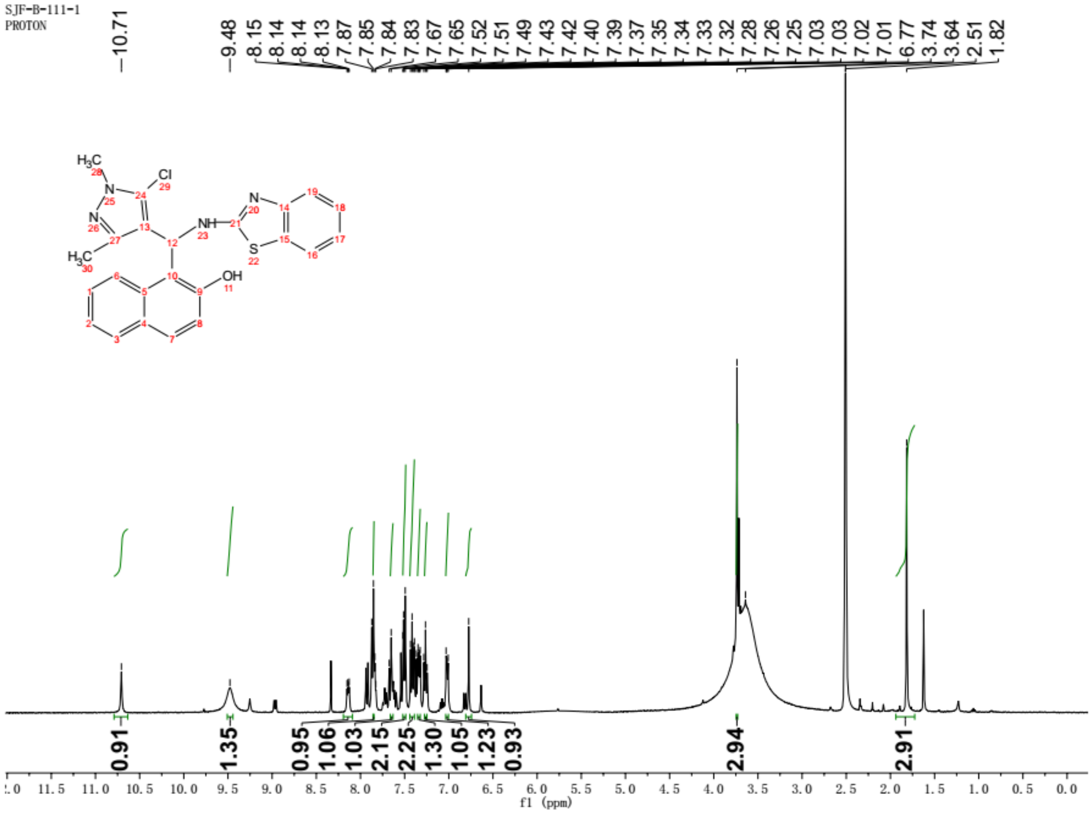

Figure S16. The ^{1}H NMR spectrum of compound **8e** (400 MHz, DMSO-d_6).

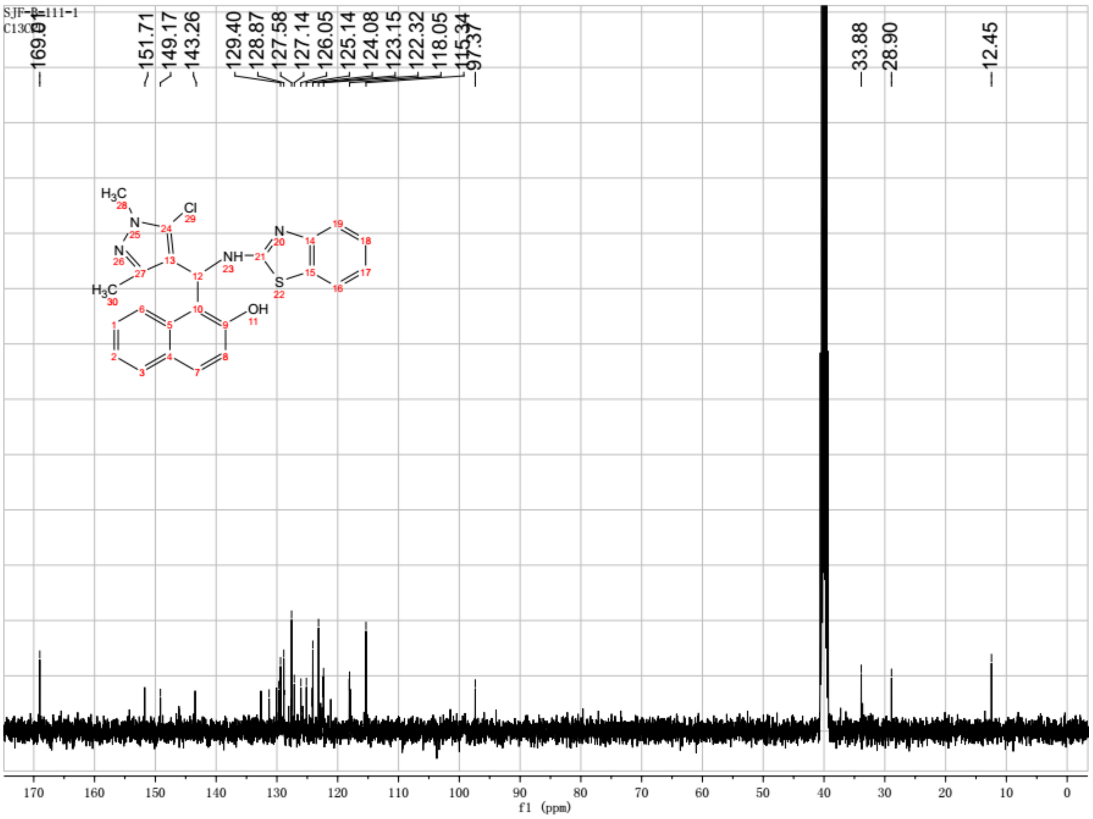

Figure S17. The ^{13}C NMR spectrum of compound **8e** (101 MHz, DMSO-d_6).

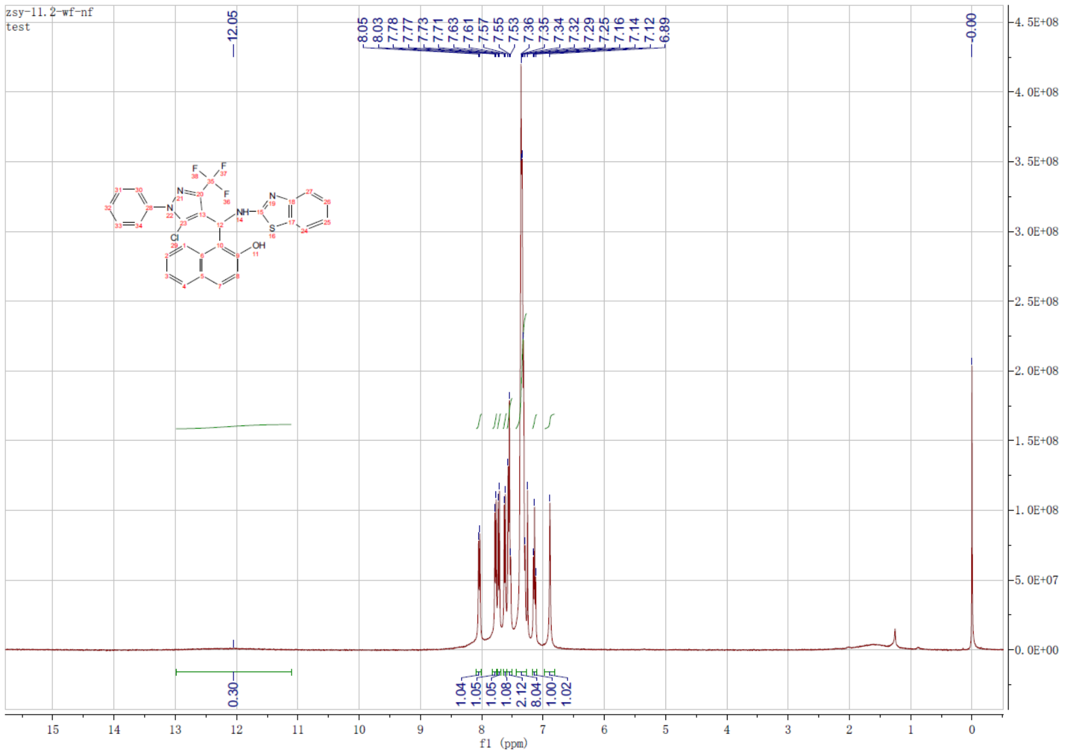

Figure S18. The ^{1}H NMR spectrum of compound **8f** (400 MHz, $CDCl_3$).

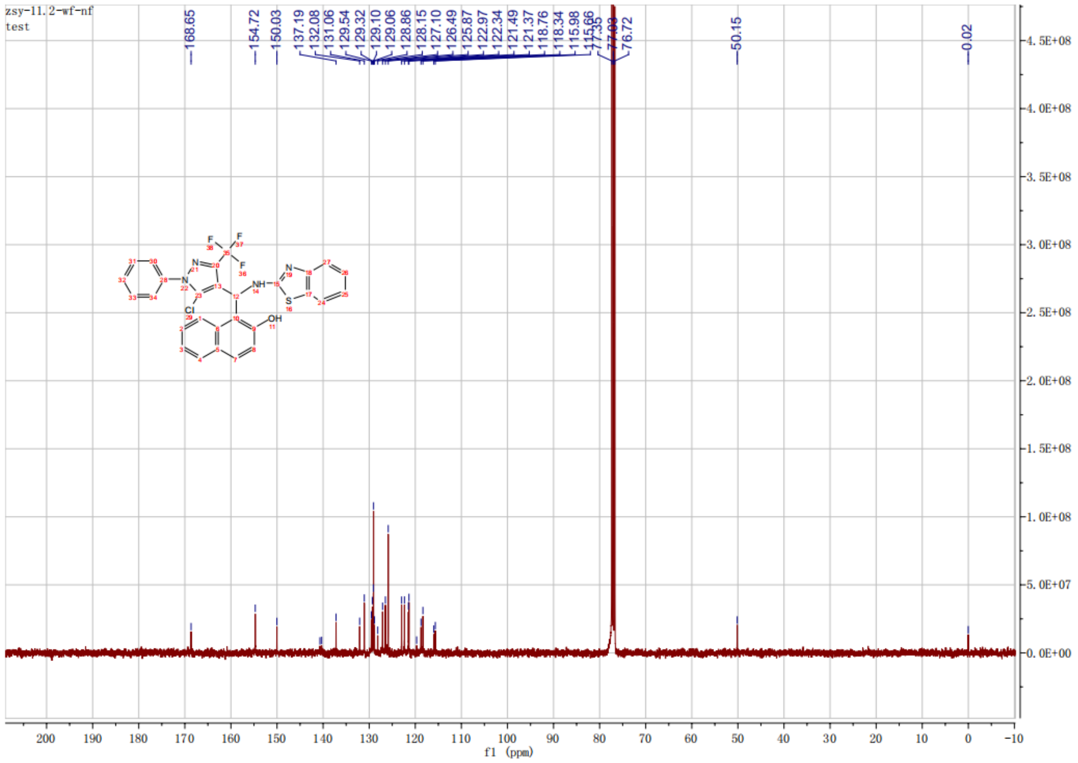

Figure S19. The ^{13}C NMR spectrum of compound **8f** (101 MHz, $CDCl_3$).

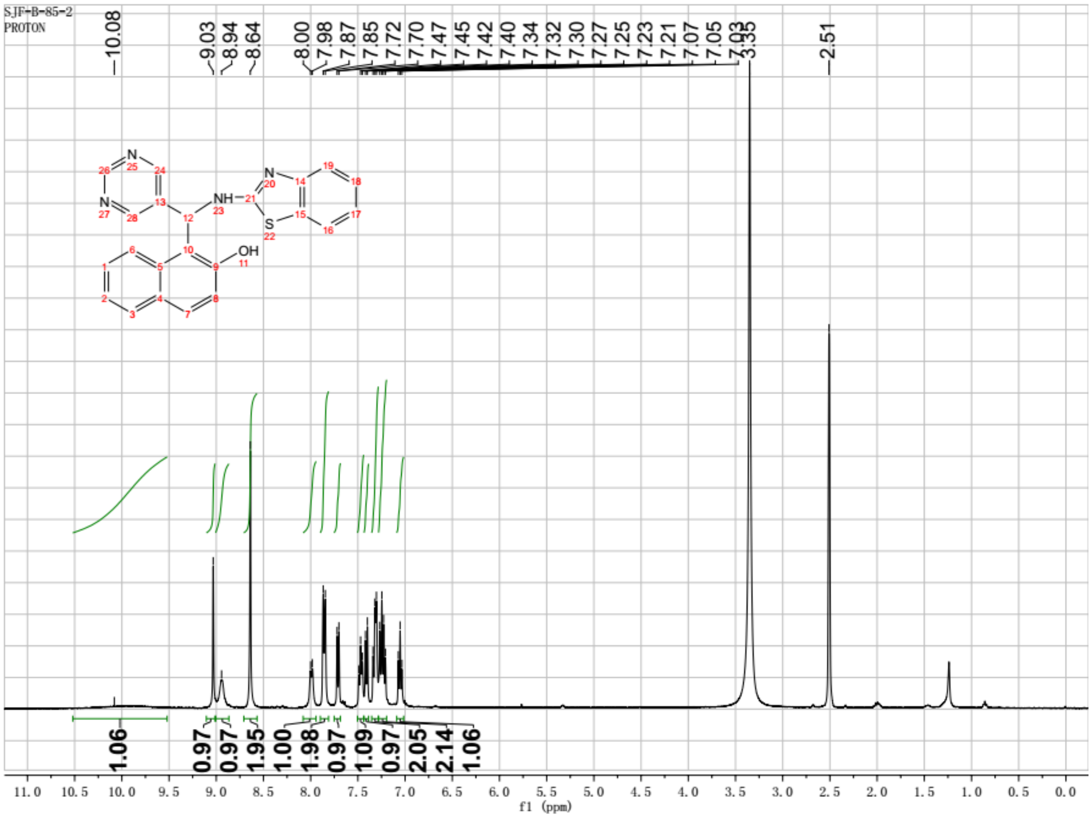

Figure S20. The ^{1}H NMR spectrum of compound **8g** (400 MHz, DMSO-d_6).

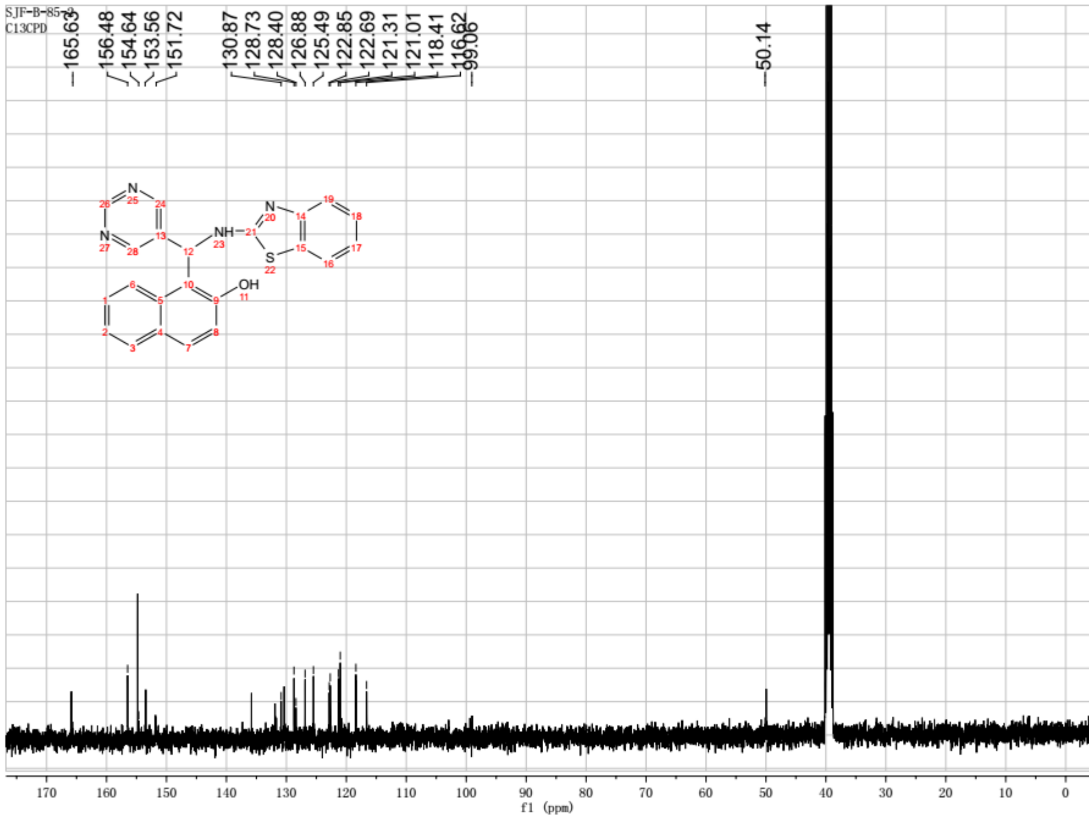

Figure S21. The ^{13}C NMR spectrum of compound **8g** (101 MHz, DMSO-d_6).

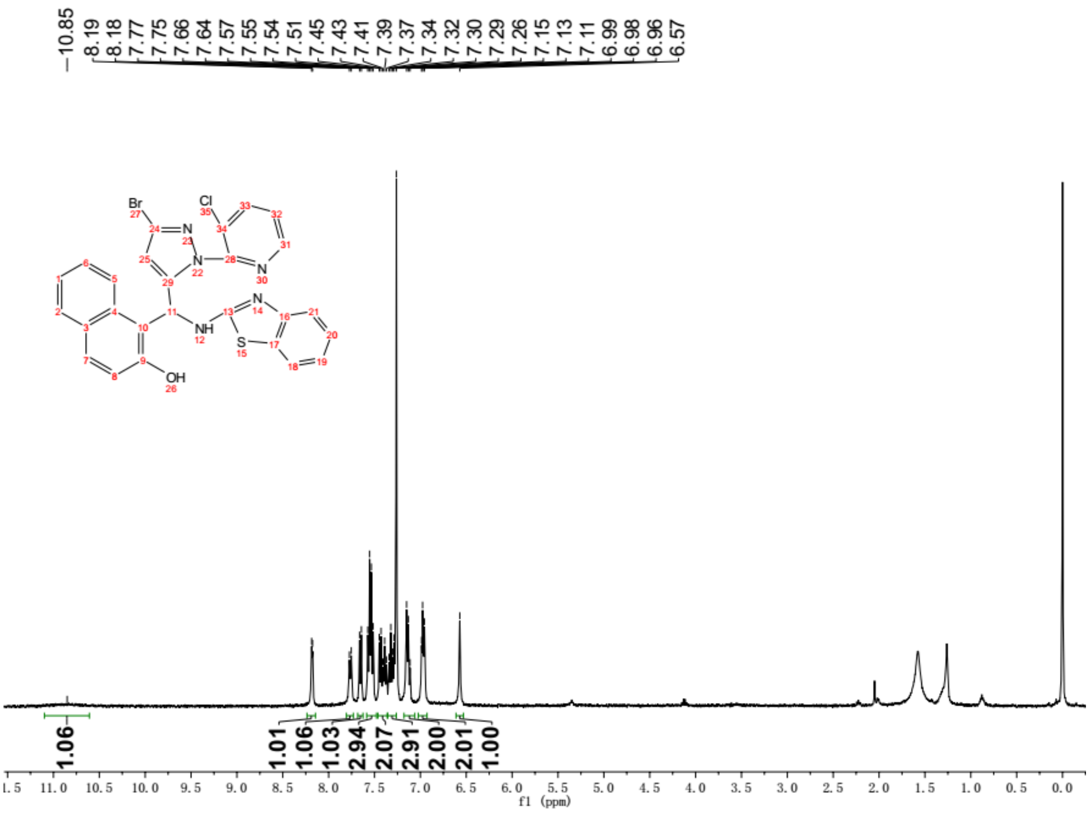

Figure S22. The ^{1}H NMR spectrum of compound **8h** (400 MHz, $CDCl_3$).

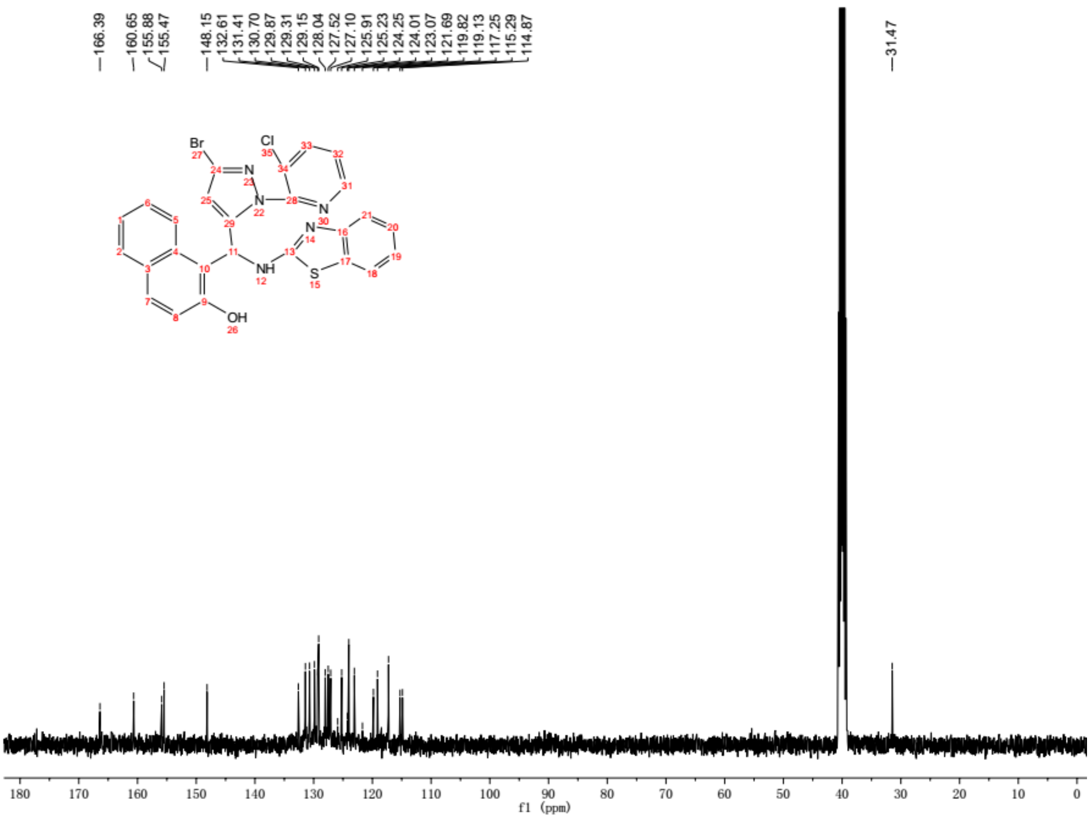

Figure S23. The ^{13}C NMR spectrum of compound **8h** (101 MHz, DMSO-d_6).

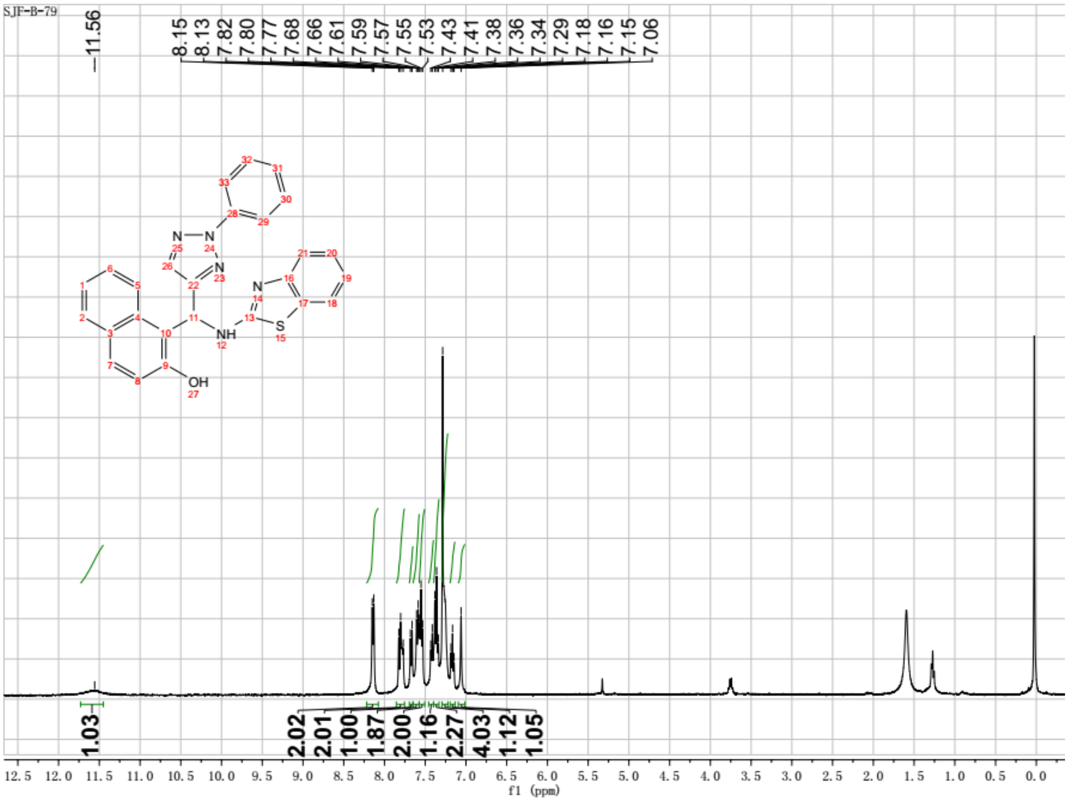

Figure S24. The ^{1}H NMR spectrum of compound **8i** (400 MHz, $CDCl_3$).

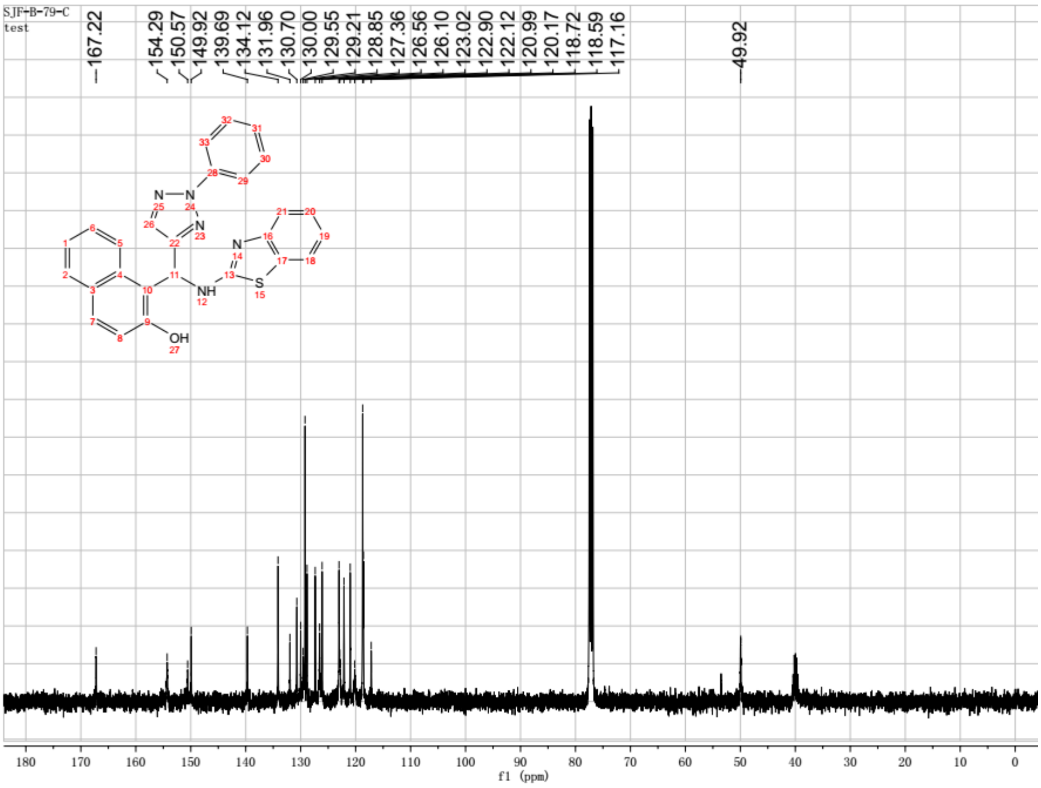

Figure S25. The ^{13}C NMR spectrum of compound **8i** (101 MHz, $CDCl_3$, containing little DMSO-d_6).

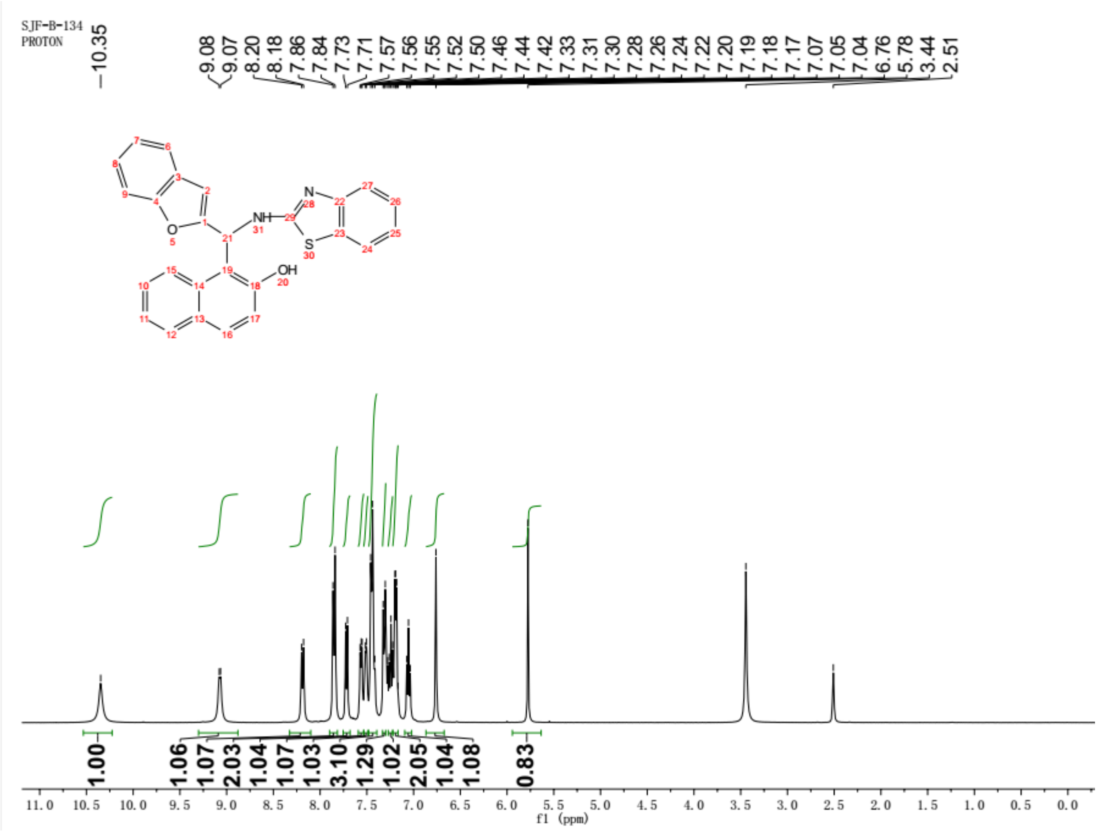

Figure S26. The ^{1}H NMR spectrum of compound **8j** (400 MHz, DMSO-d_6).

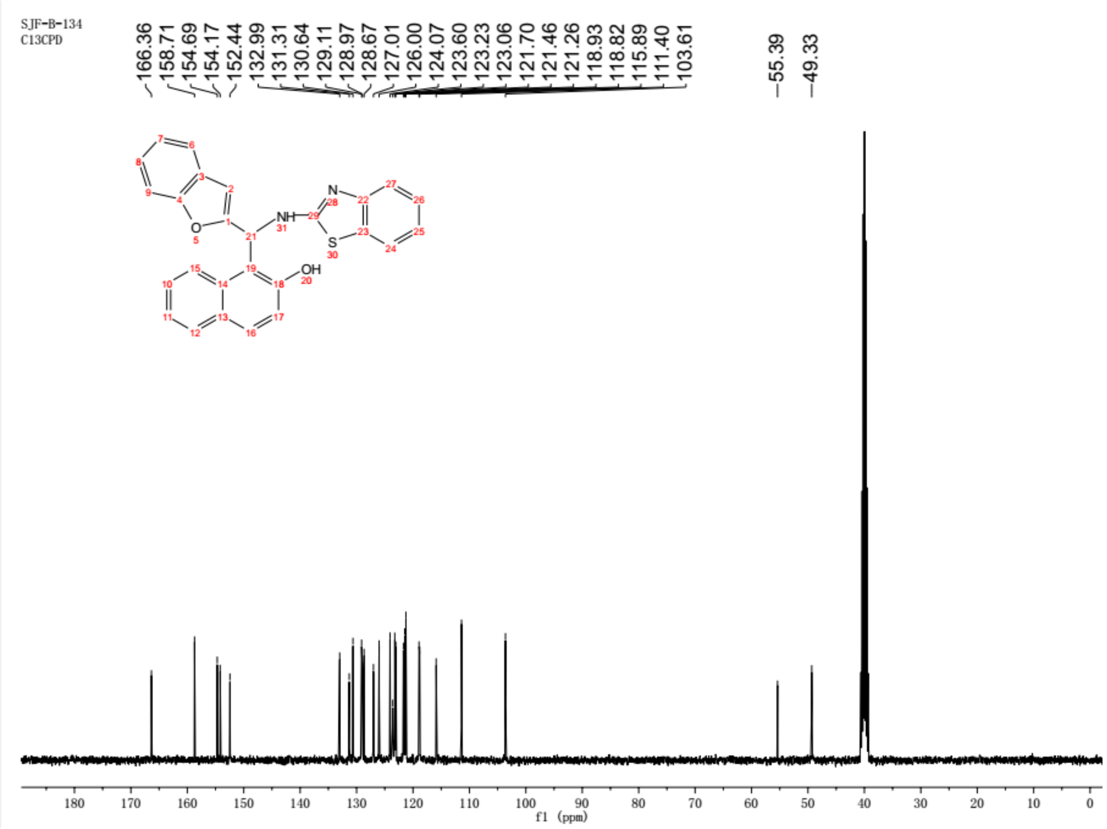

Figure S27. The ^{13}C NMR spectrum of compound **8j** (101 MHz, DMSO-d_6).

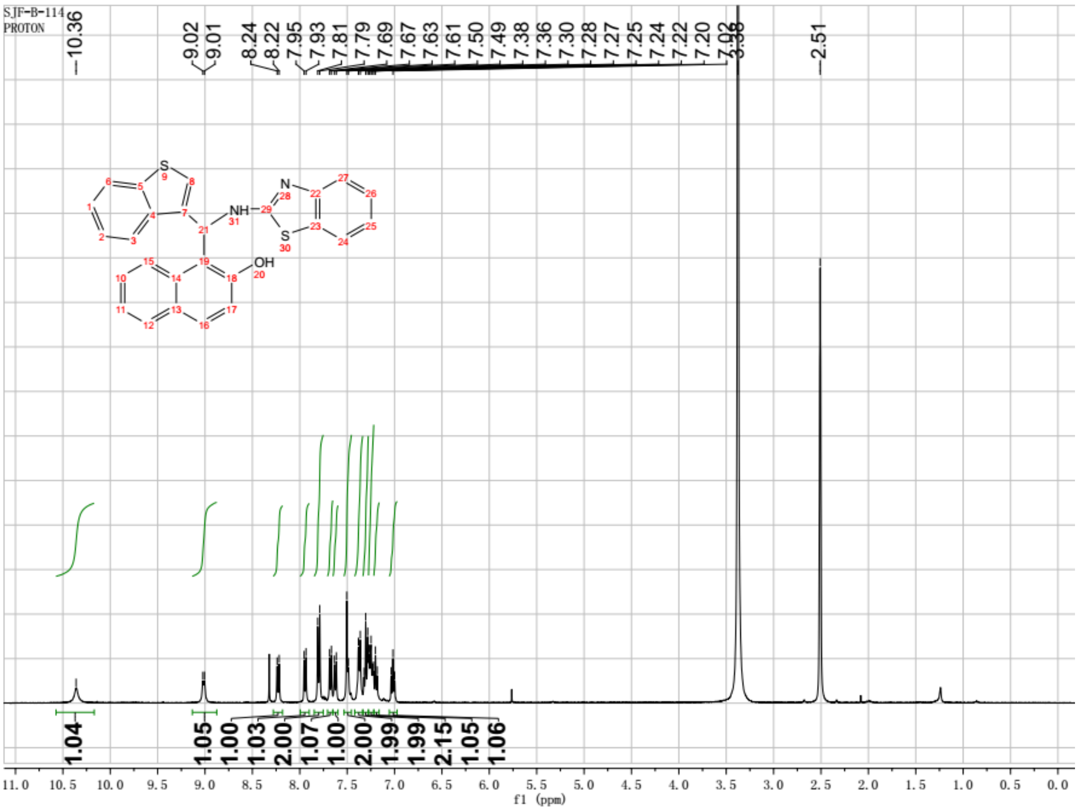

Figure S28. The ^{1}H NMR spectrum of compound **8k** (400 MHz, DMSO-d_6).

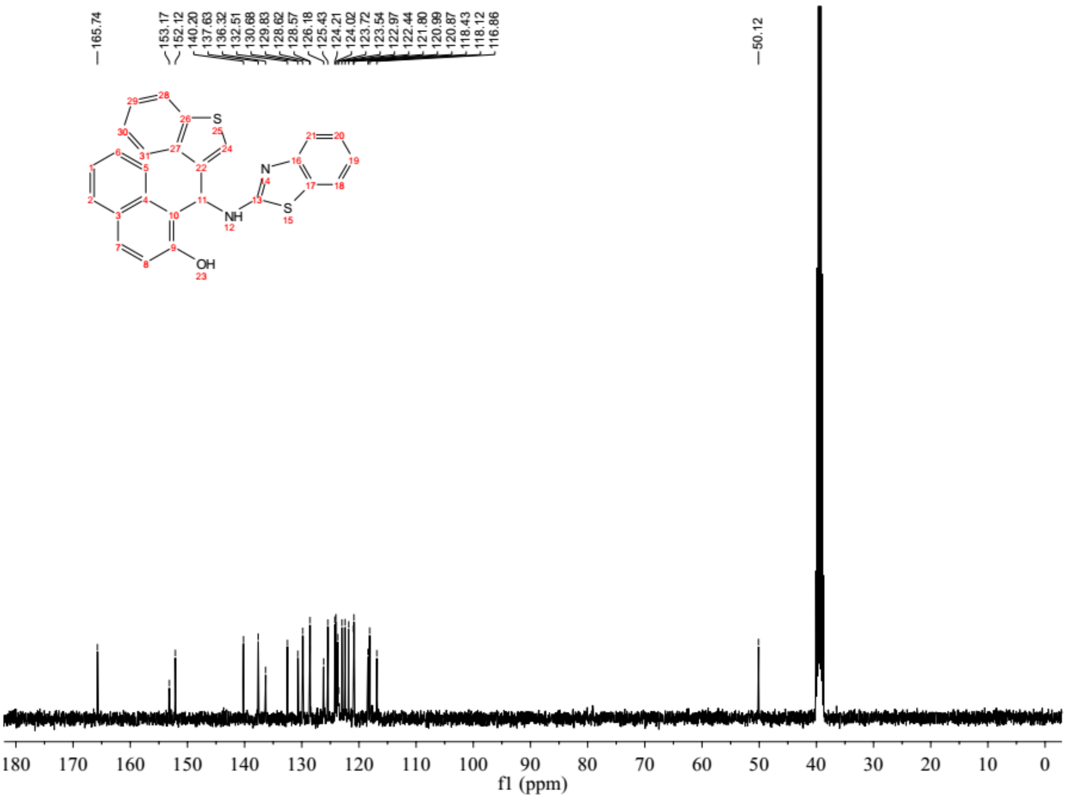

Figure S29. The ^{13}C NMR spectrum of compound **8k** (101 MHz, DMSO-d_6).

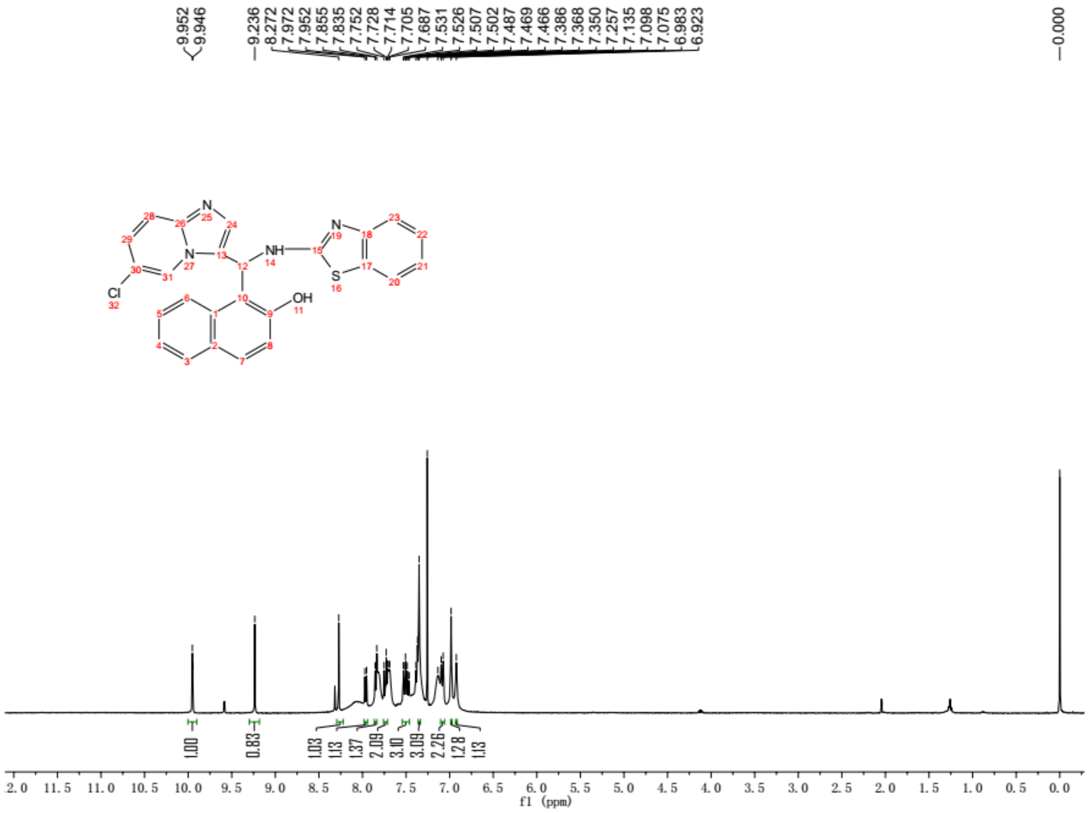

Figure S30. The ^{1}H NMR spectrum of compound **8l** (400 MHz, $CDCl_3$).

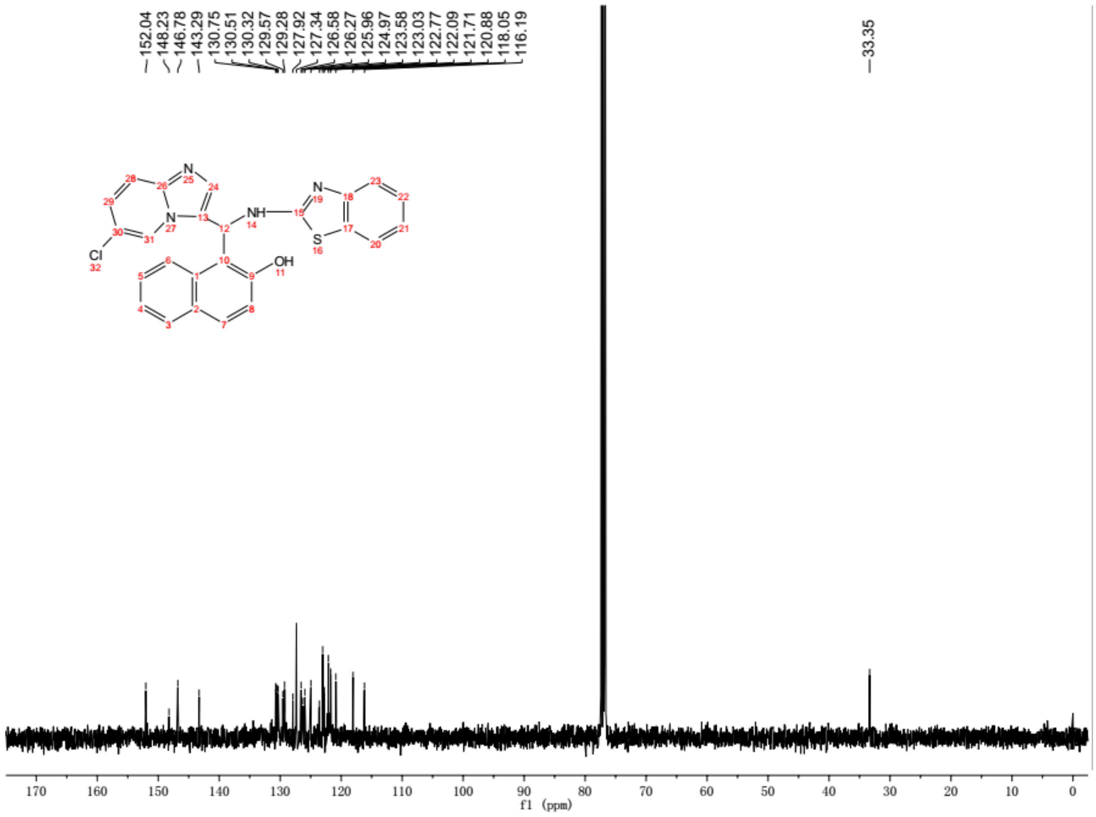

Figure S31. The ^{13}C NMR spectrum of compound **8l** (101 MHz, $CDCl_3$).

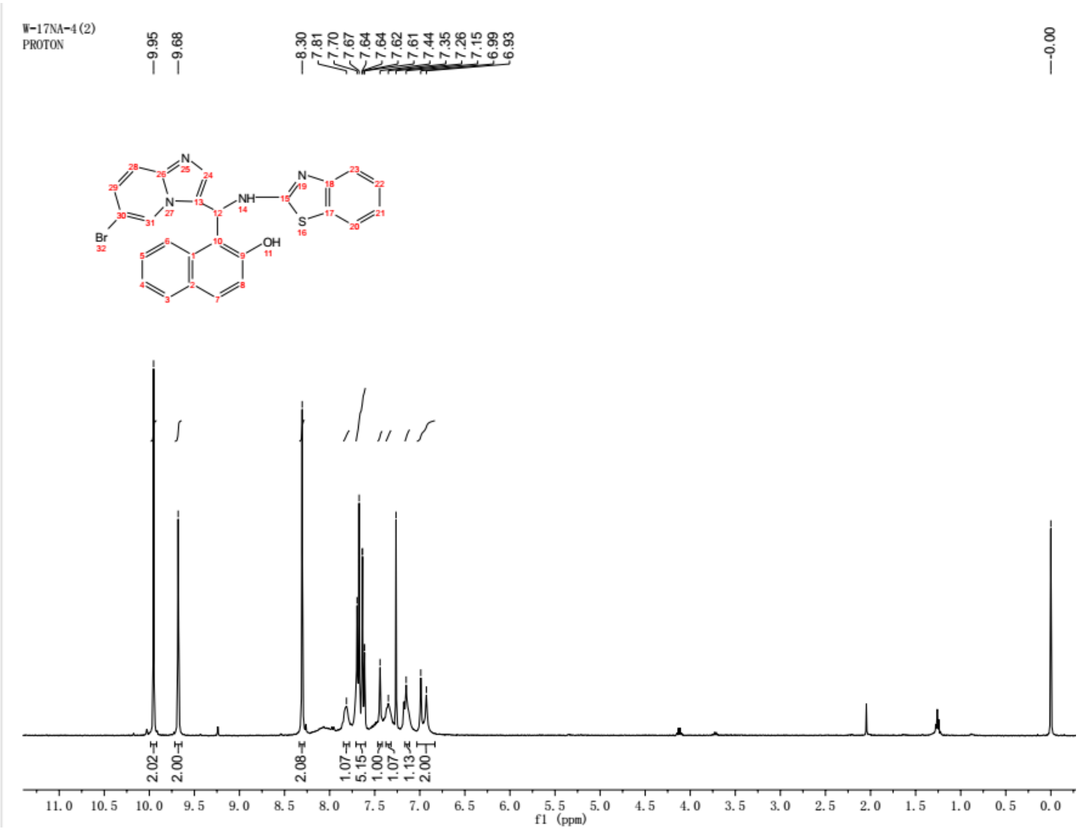

Figure S32. The ^{1}H NMR spectrum of compound **8m** (400 MHz, $CDCl_3$).

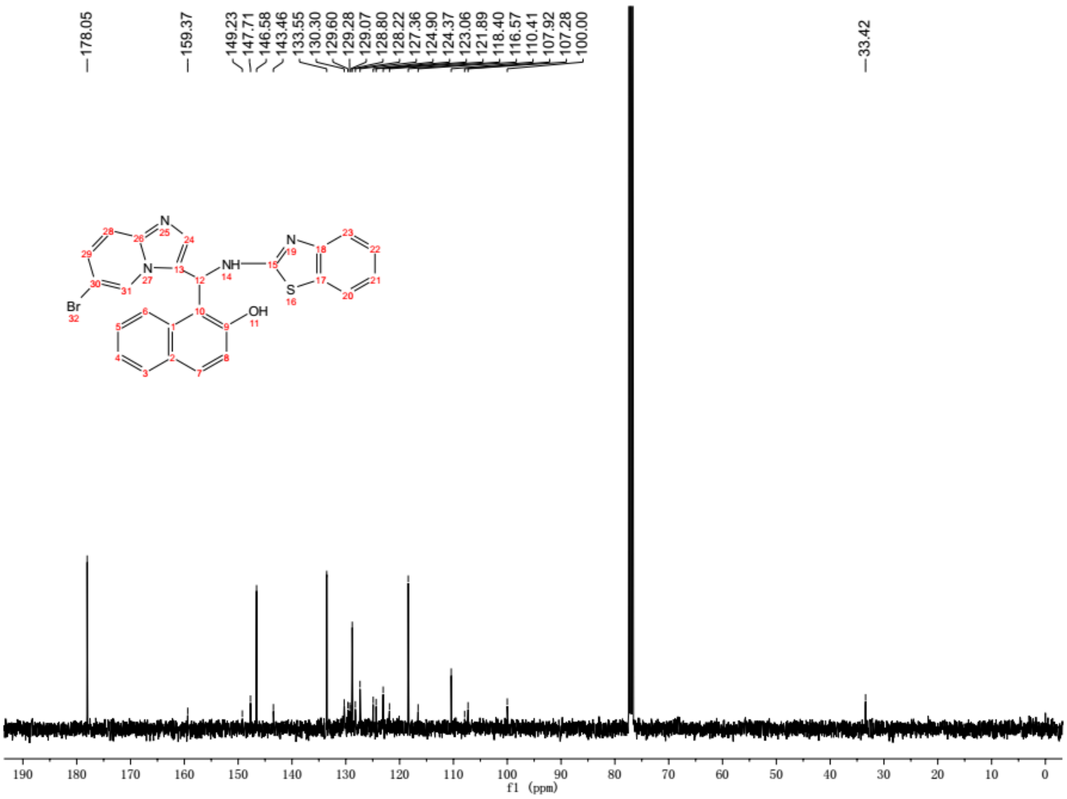

Figure S33. The ^{13}C NMR spectrum of compound **8m** (101 MHz, $CDCl_3$).

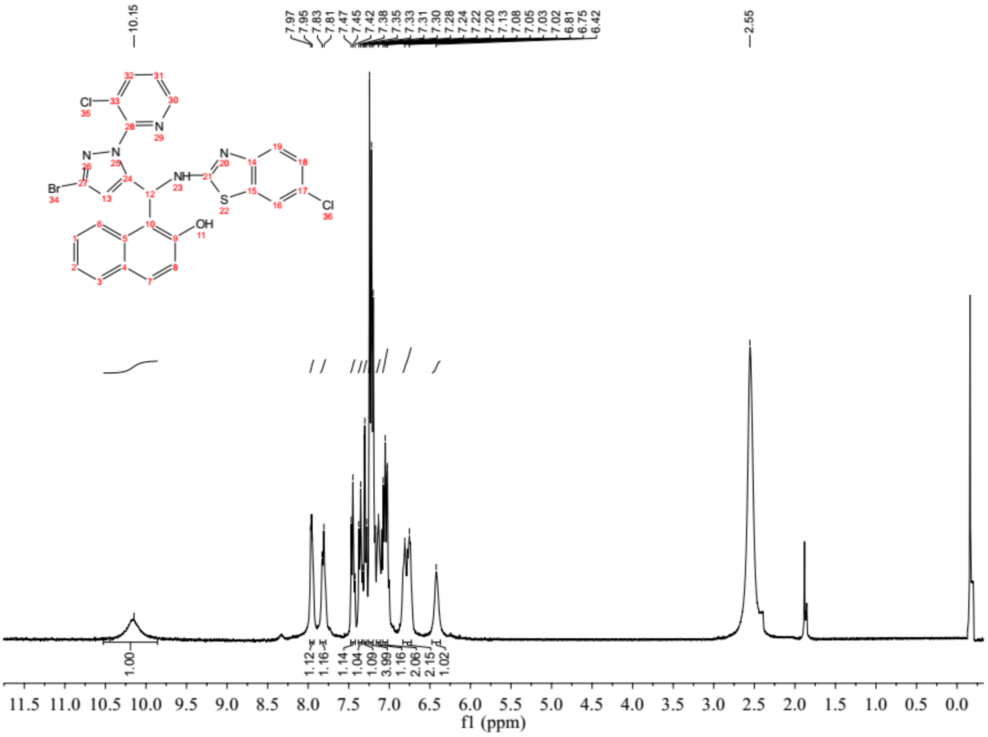

Figure S34. The ^{1}H NMR spectrum of compound **8n** (400 MHz, $CDCl_3$, containing little DMSO-d_6).

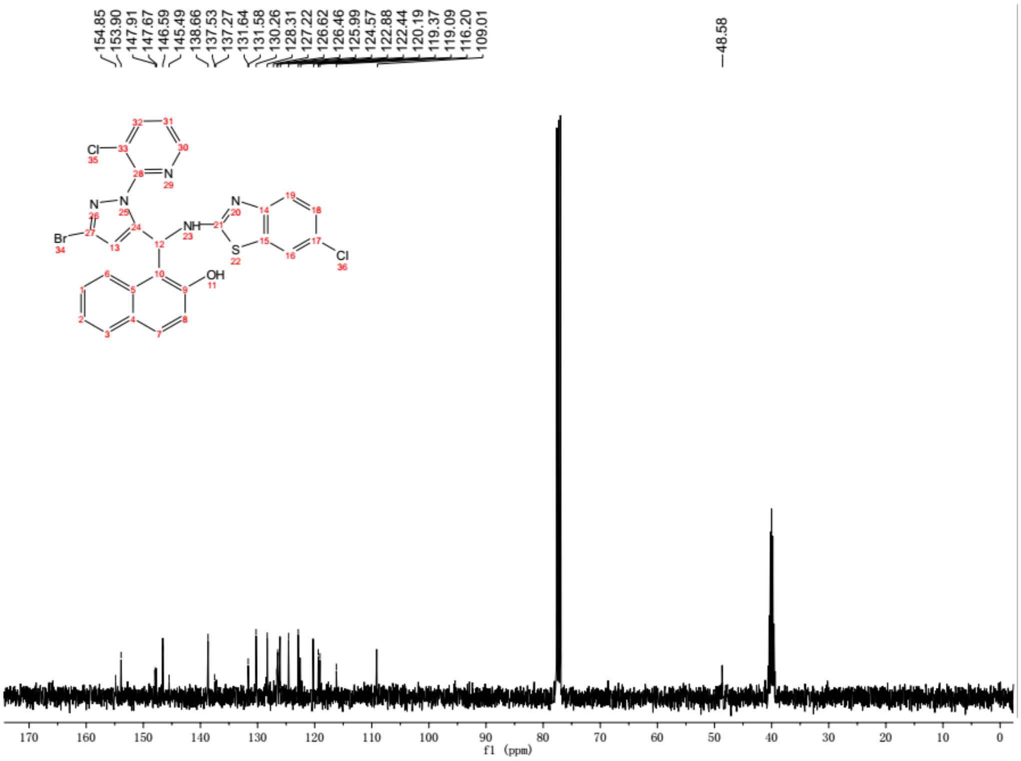

Figure S35. The ^{13}C NMR spectrum of compound **8n** (101 MHz, $CDCl_3$, containing little DMSO-d_6).

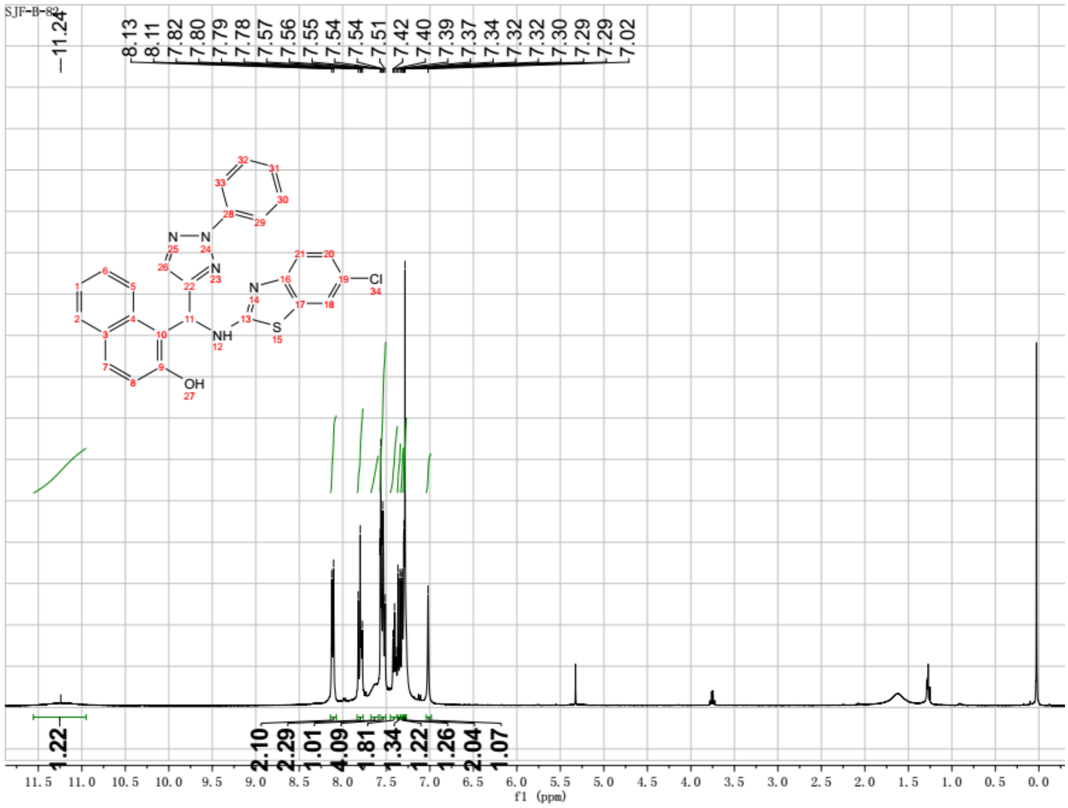

Figure S36. The ^{1}H NMR spectrum of compound **8o** (400 MHz, $CDCl_3$).

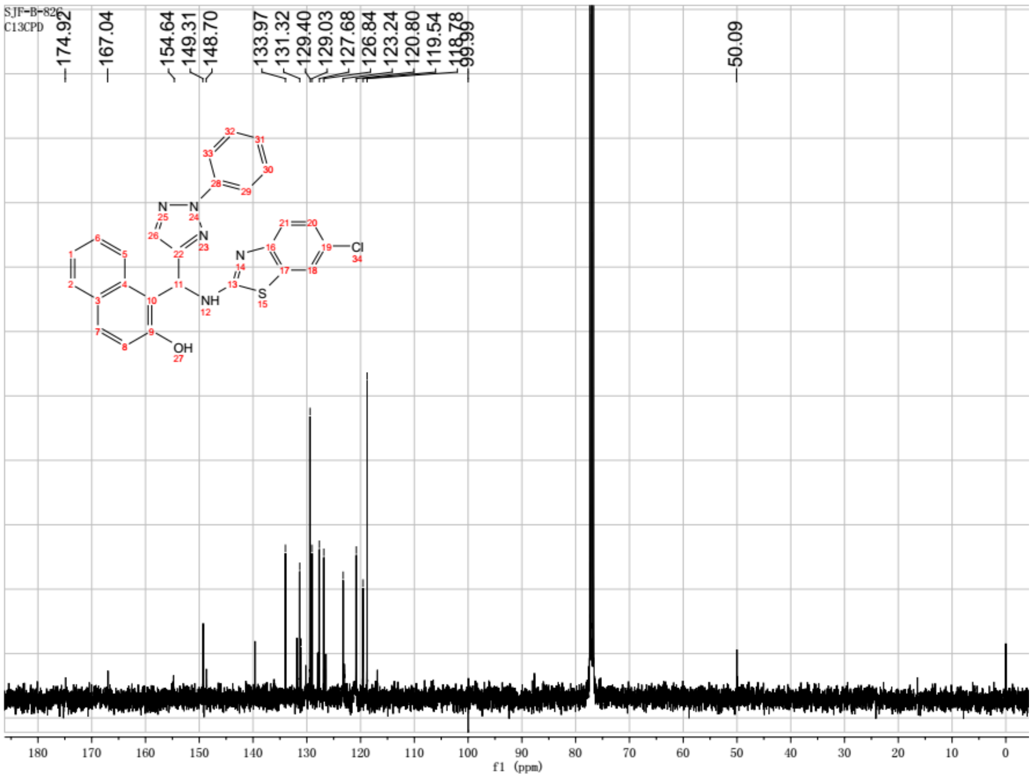

Figure S37. The ^{13}C NMR spectrum of compound **8o** (101 MHz, $CDCl_3$).

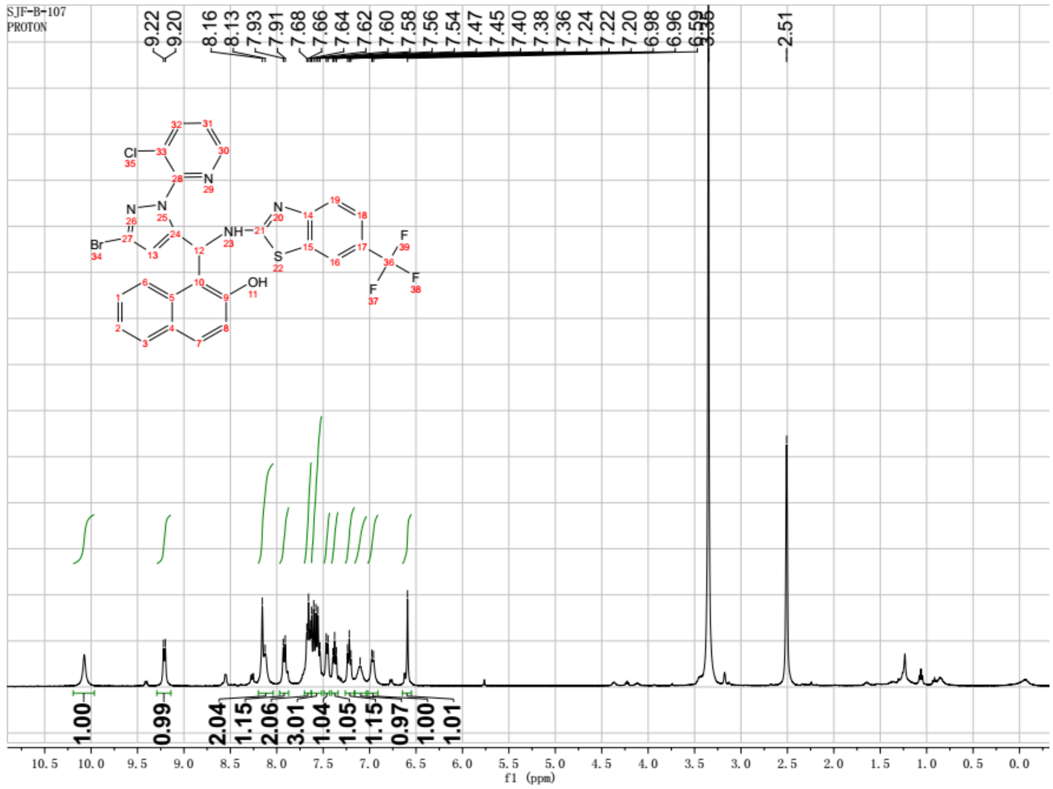

Figure S38. The ^{1}H NMR spectrum of compound **8p** (400 MHz, DMSO-d_6).

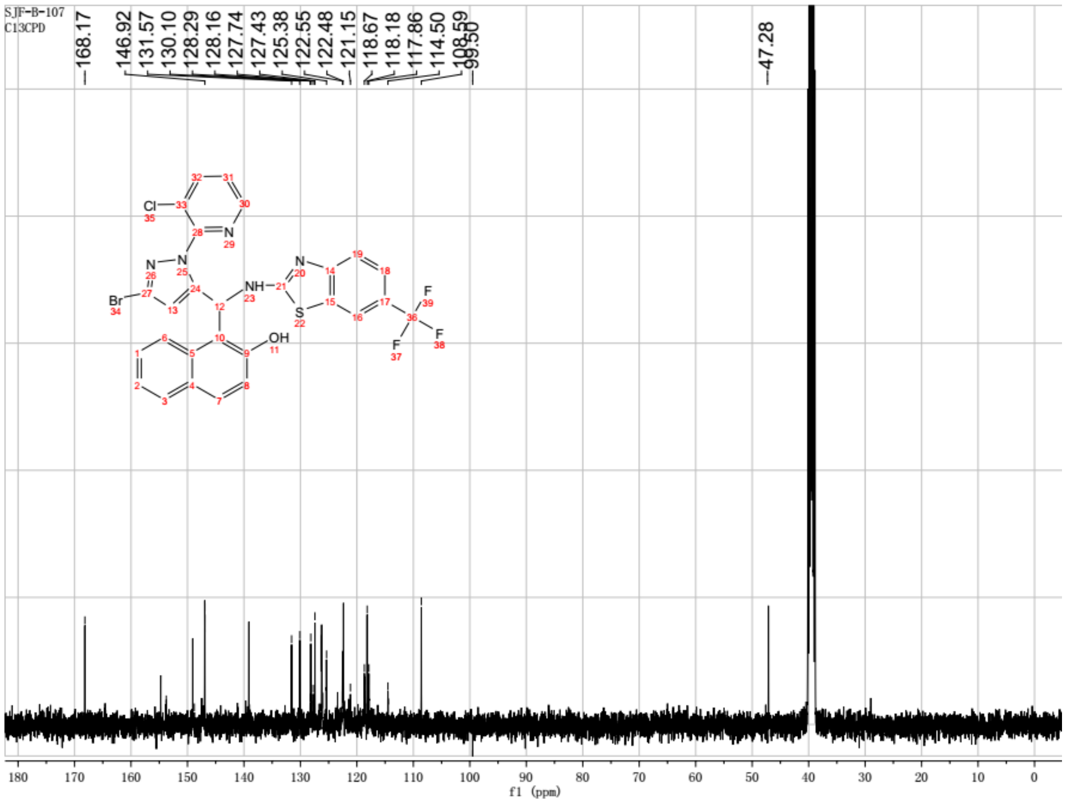

Figure S39. The ^{13}C NMR spectrum of compound **8p** (101 MHz, DMSO-d_6).

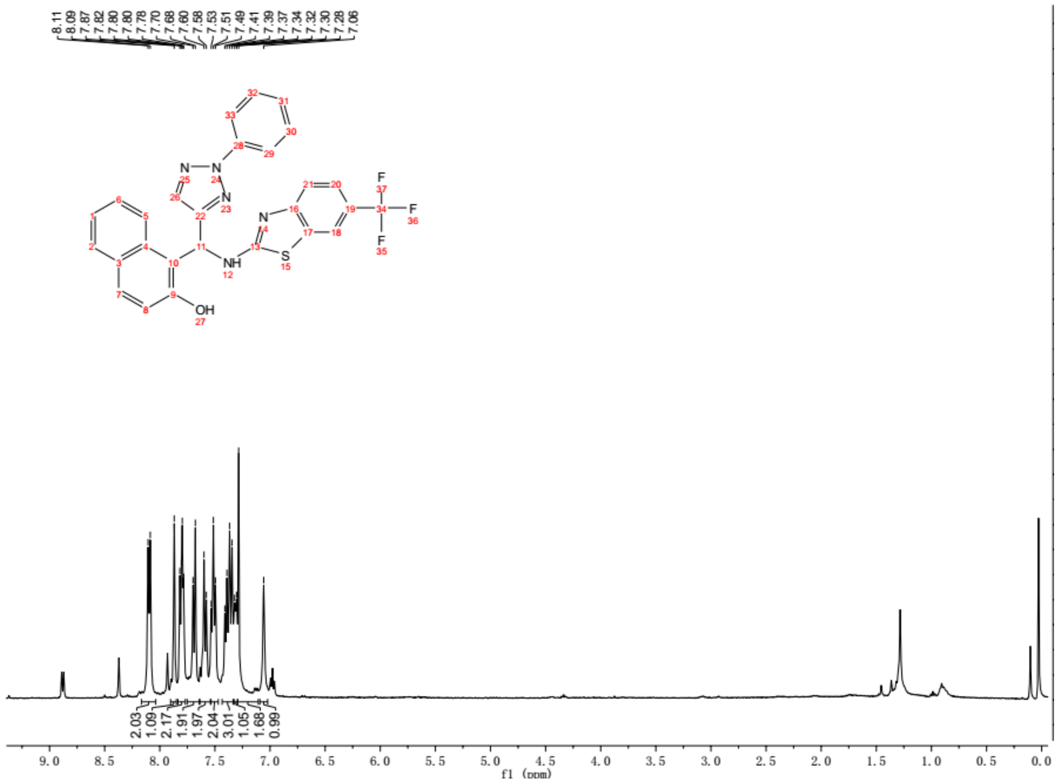

Figure S40. The ^{1}H NMR spectrum of compound **8q** (400 MHz, $CDCl_3$).

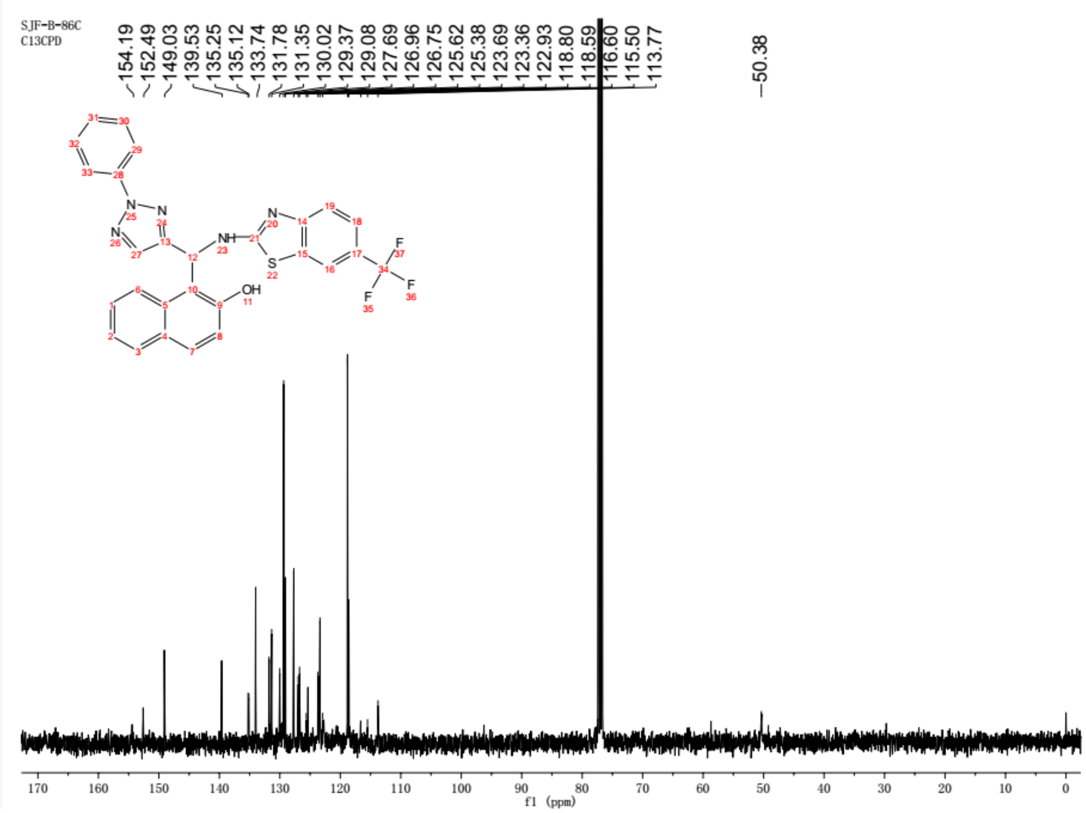

Figure S41. The ^{13}C NMR spectrum of compound **8q** (101 MHz, $CDCl_3$).

3. The HRMS spectra of the title compounds 8a-q

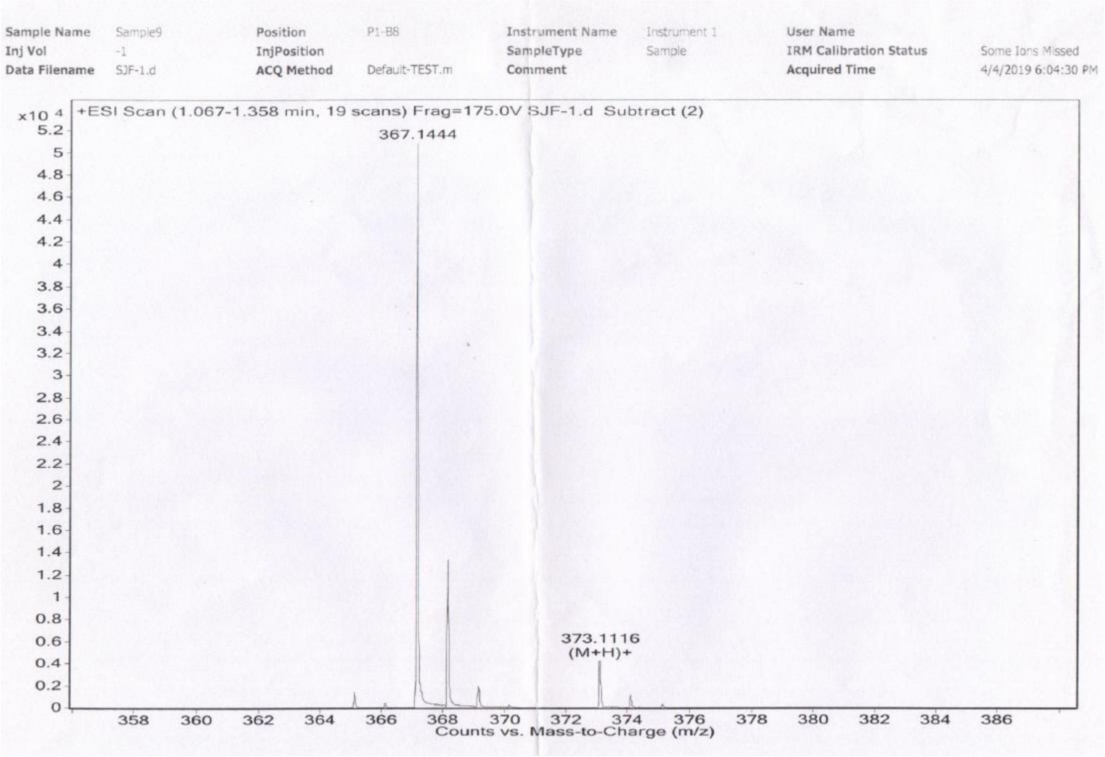

Figure S42. The HRMS spectrum of compound **8a**.

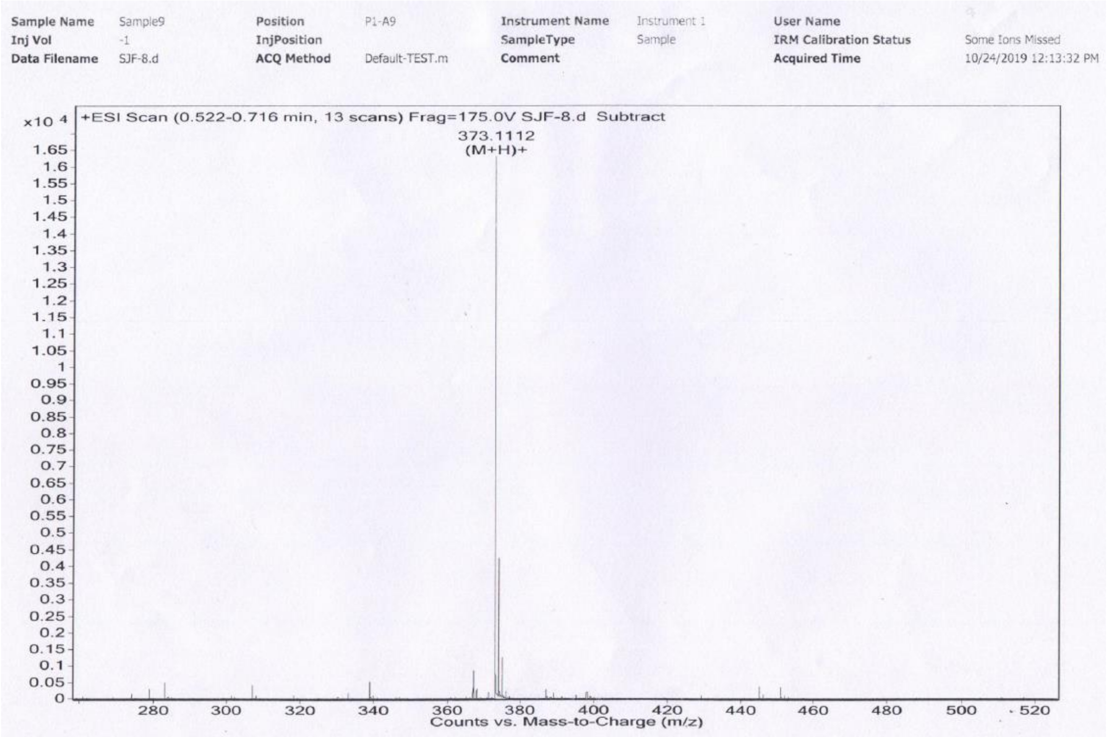

Figure S43. The HRMS spectrum of compound **8b**.

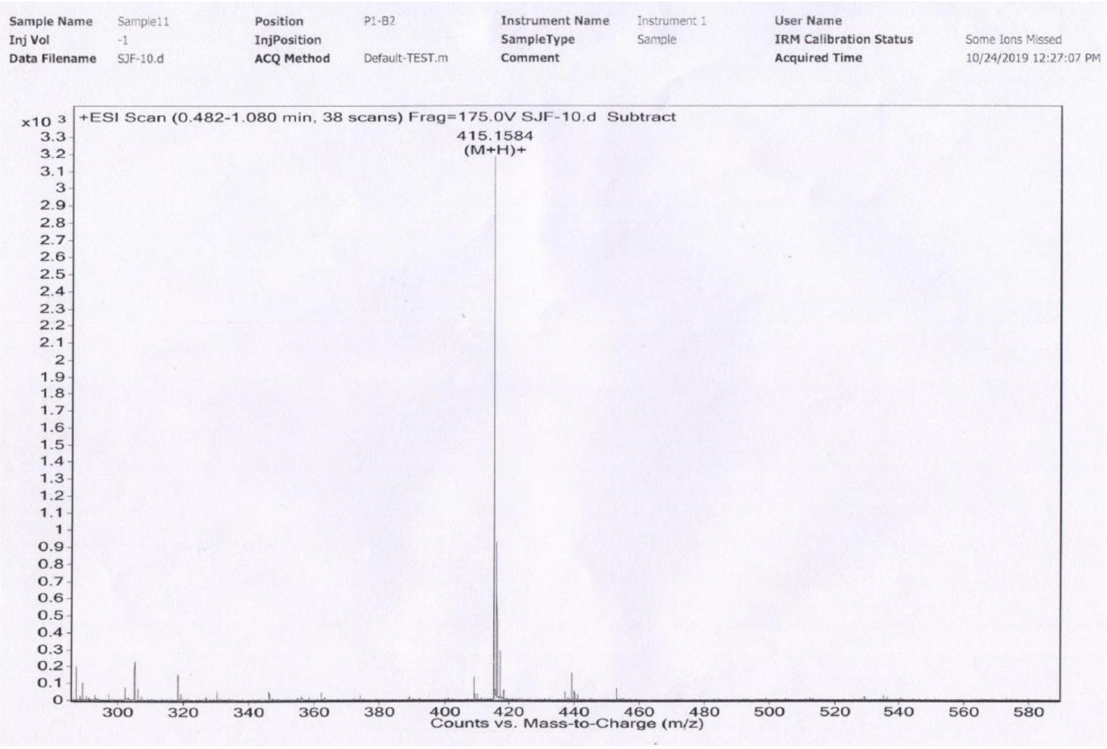

Figure S44. The HRMS spectrum of compound **8c**.

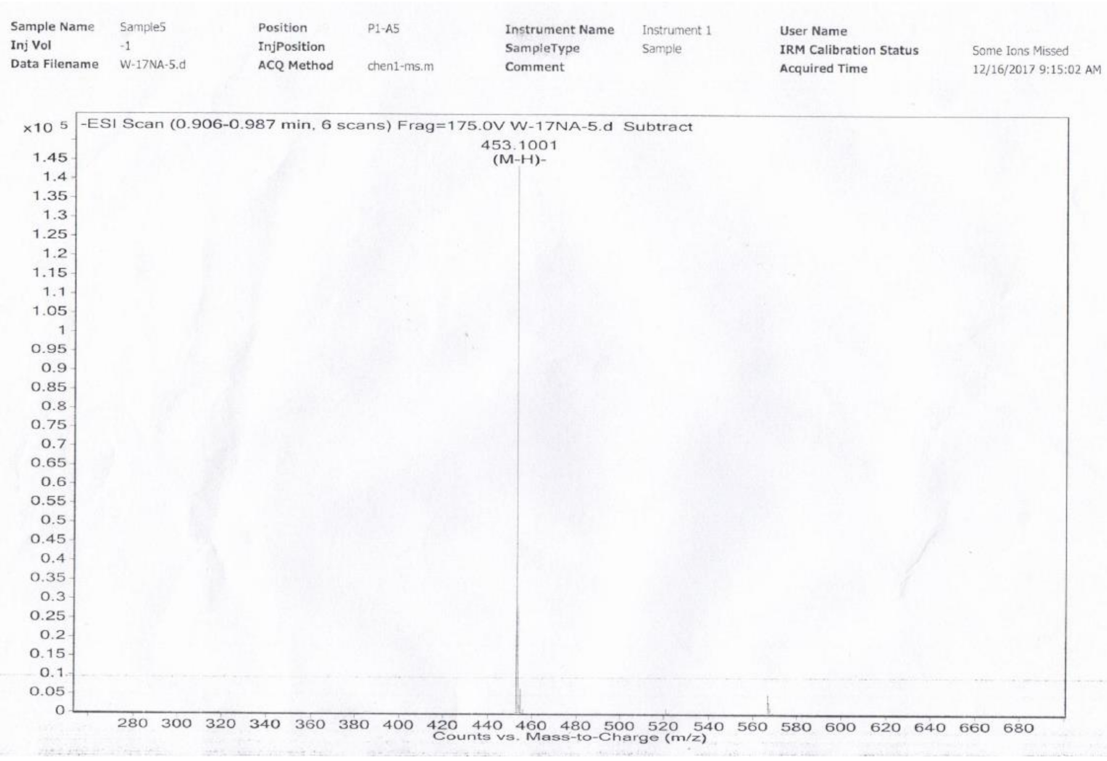

Figure S45. The HRMS spectrum of compound **8d**.

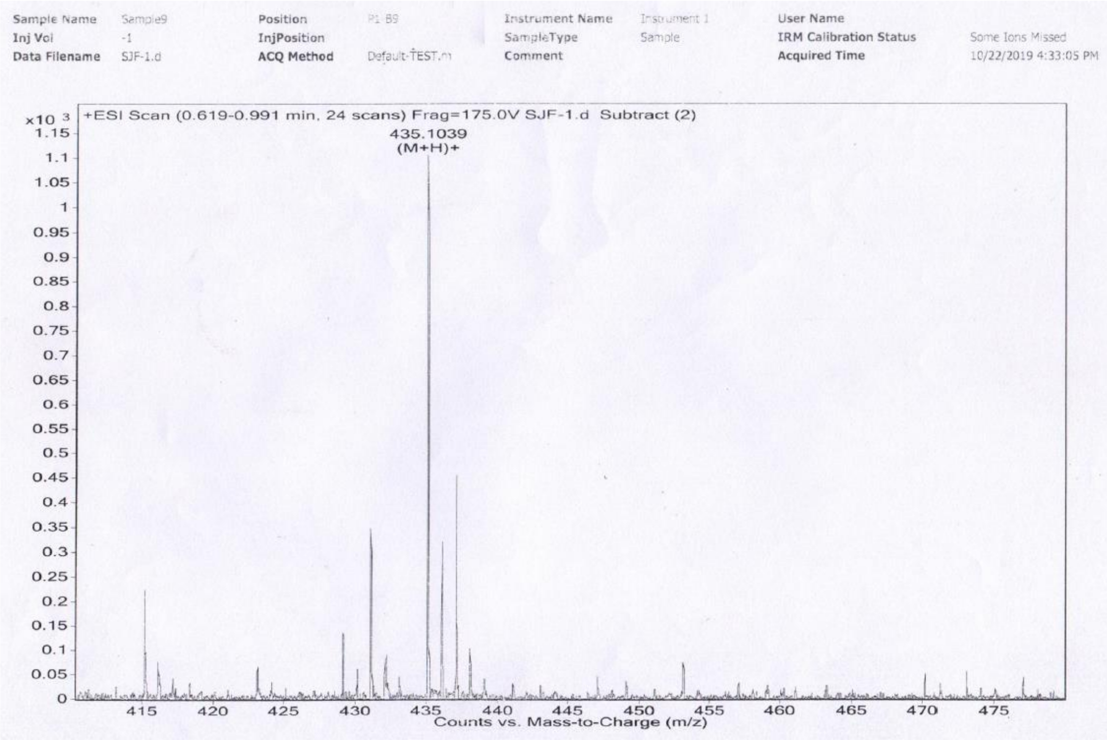

Figure S46. The HRMS spectrum of compound **8e**.

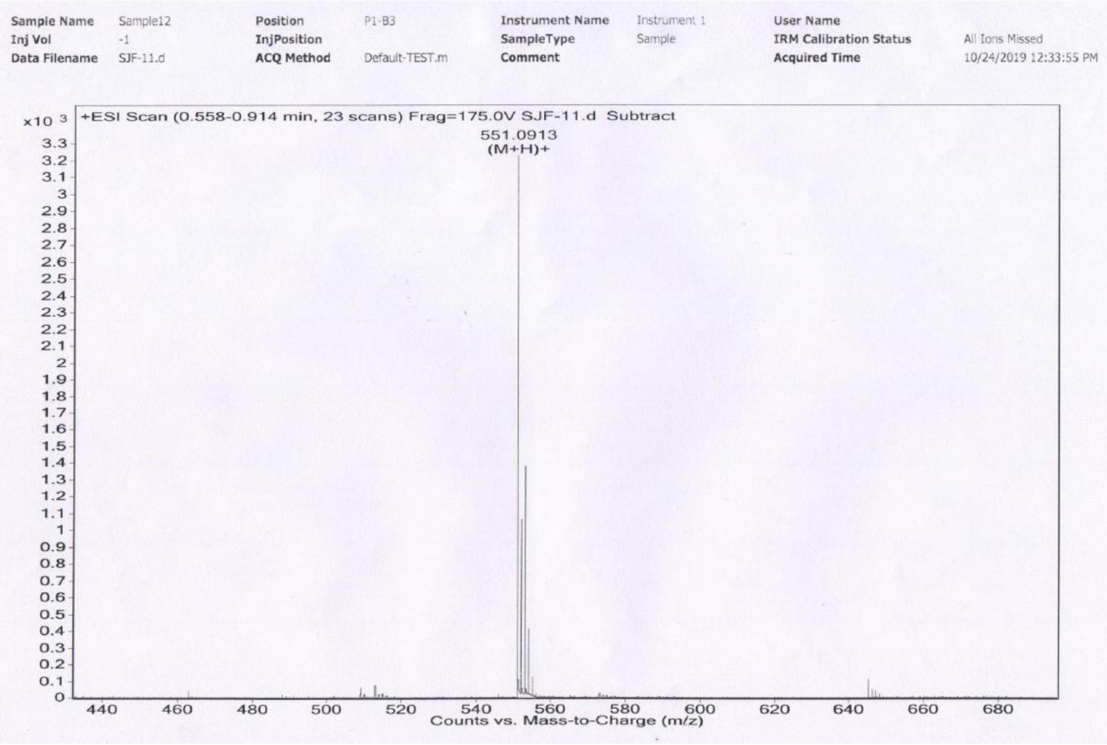

Figure S47. The HRMS spectrum of compound **8f**.

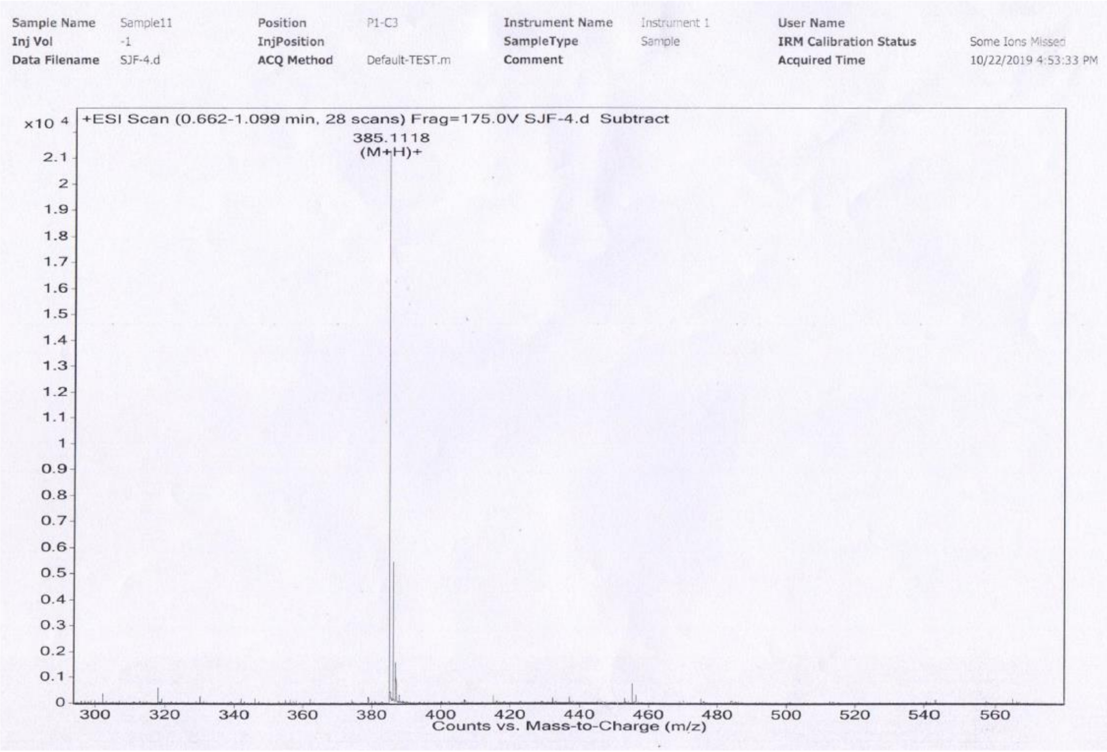

Figure S48. The HRMS spectrum of compound **8g**.

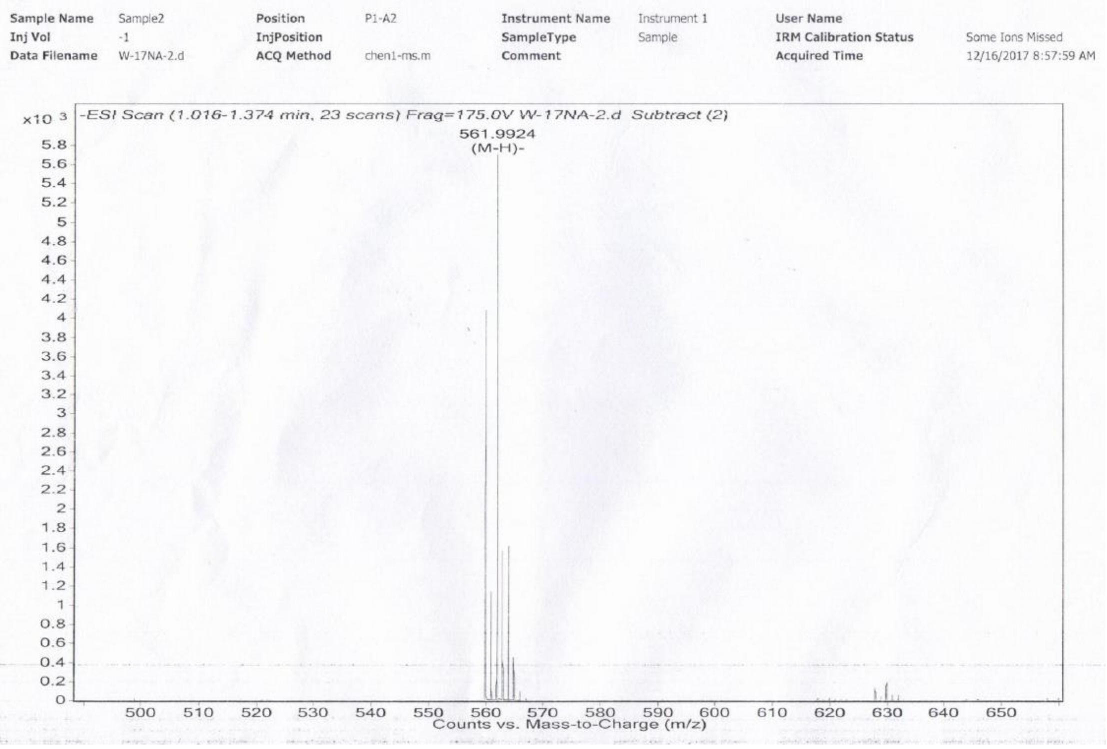

Figure S49. The HRMS spectrum of compound **8h**.

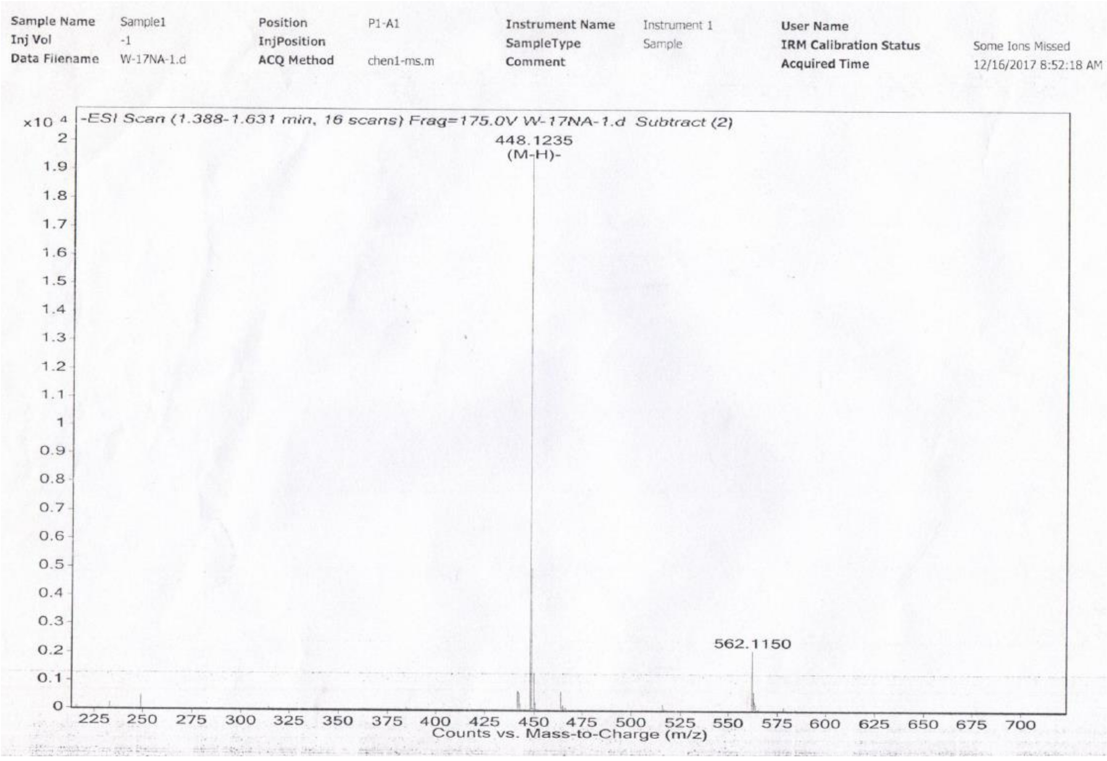

Figure S50. The HRMS spectrum of compound **8i**.

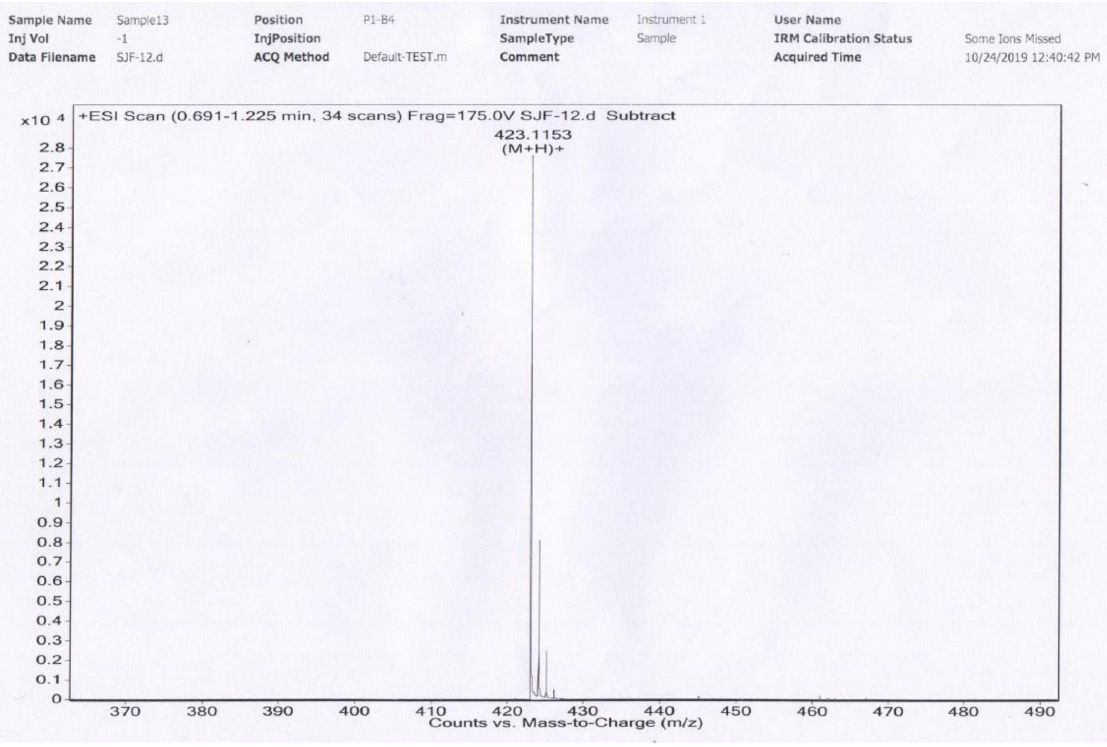

Figure S51. The HRMS spectrum of compound **8j**.

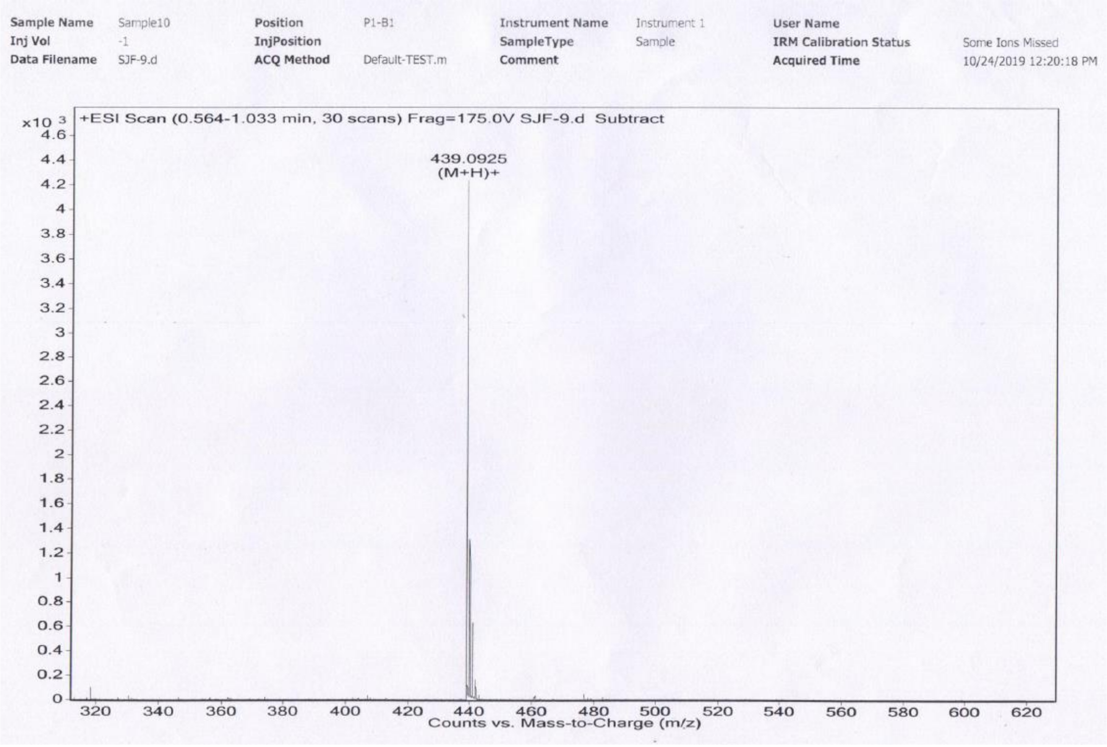

Figure S52. The HRMS spectrum of compound **8k**.

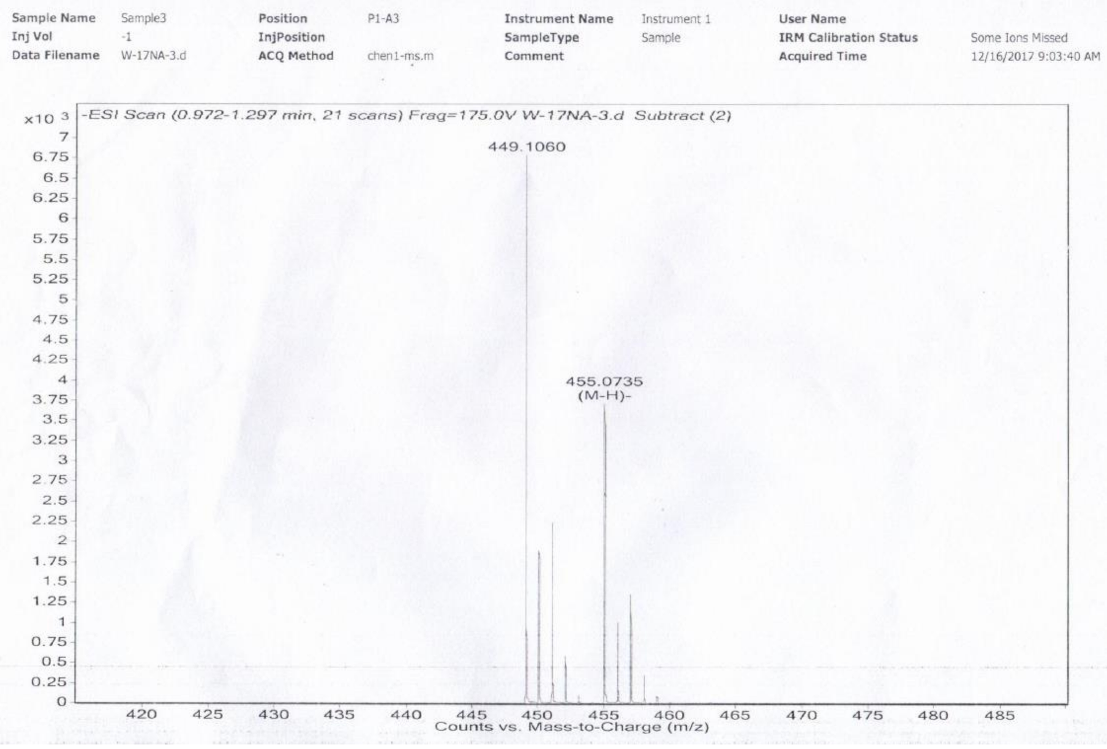

Figure S53. The HRMS spectrum of compound **8l**.

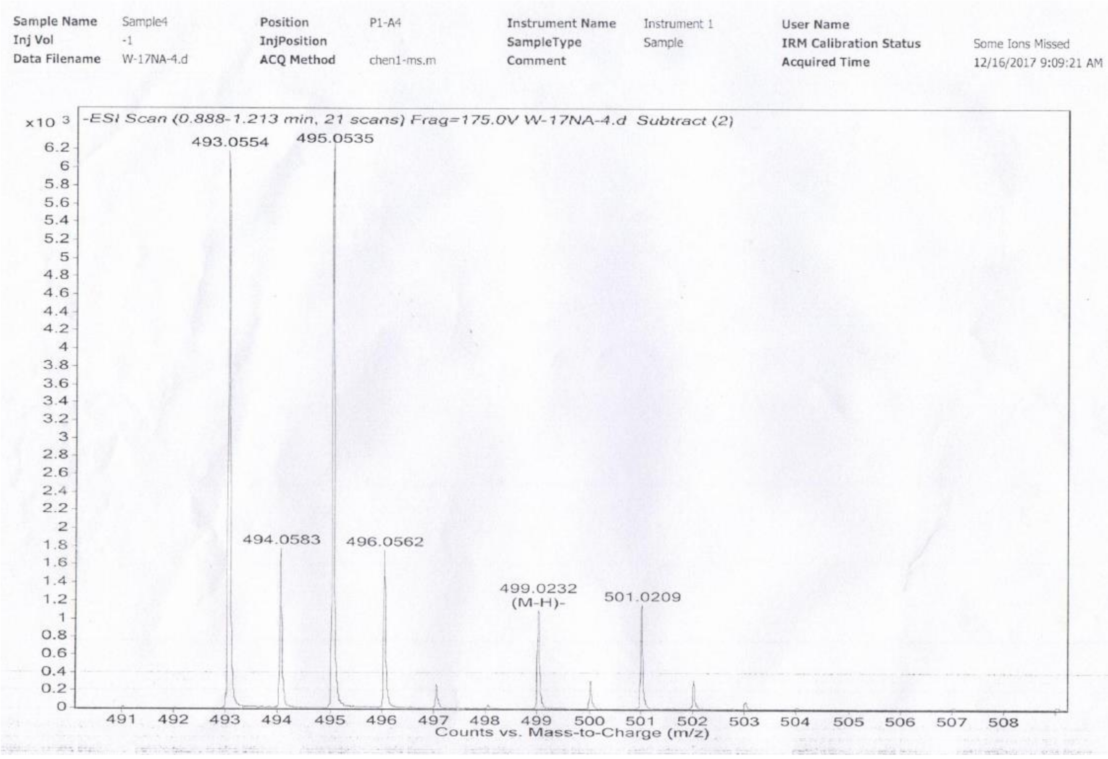

Figure S54. The HRMS spectrum of compound **8m**.

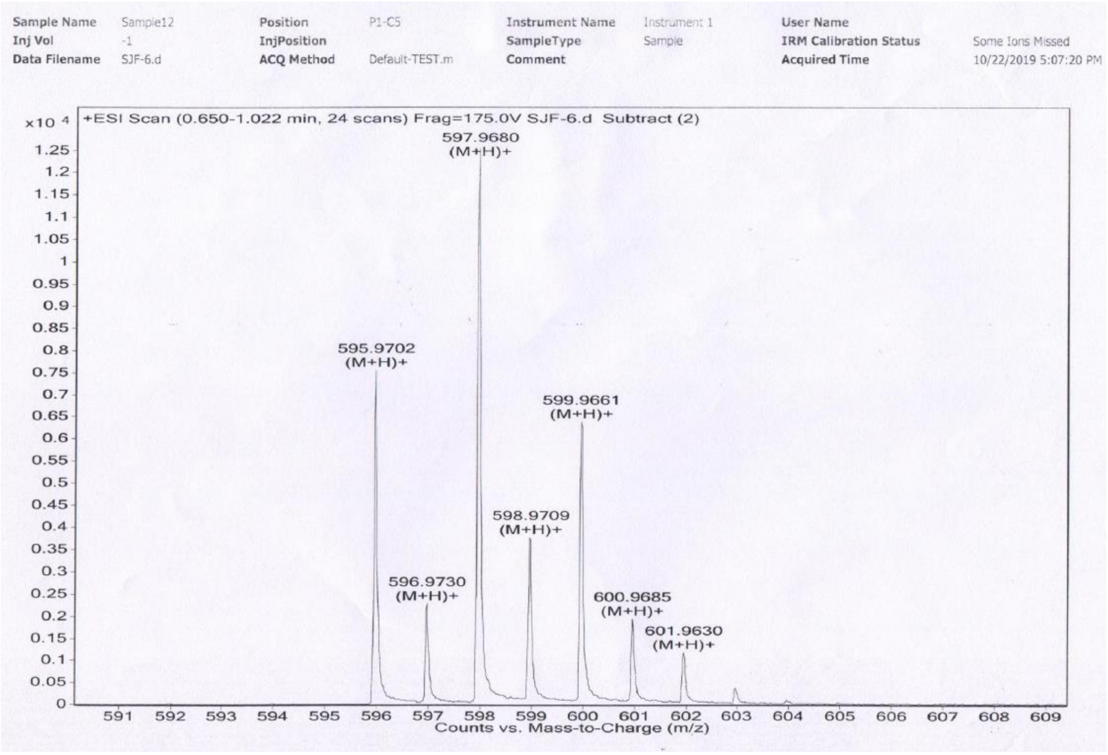

Figure S55. The HRMS spectrum of compound **8n**.

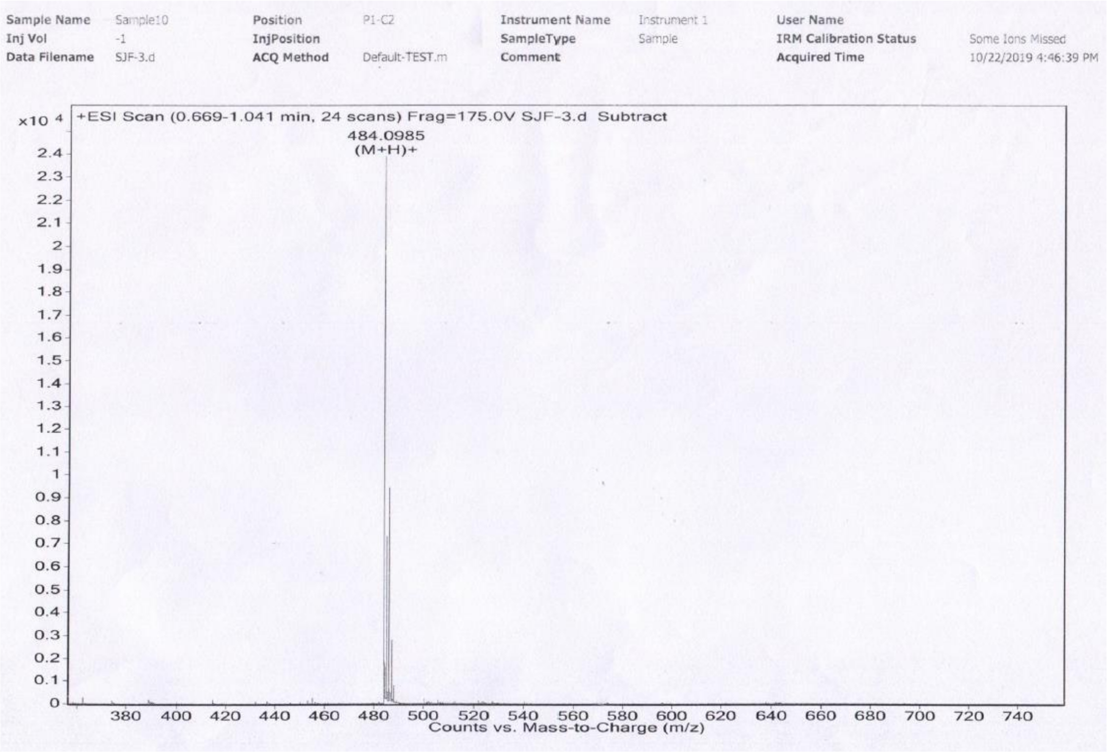

Figure S56. The HRMS spectrum of compound **8o**.

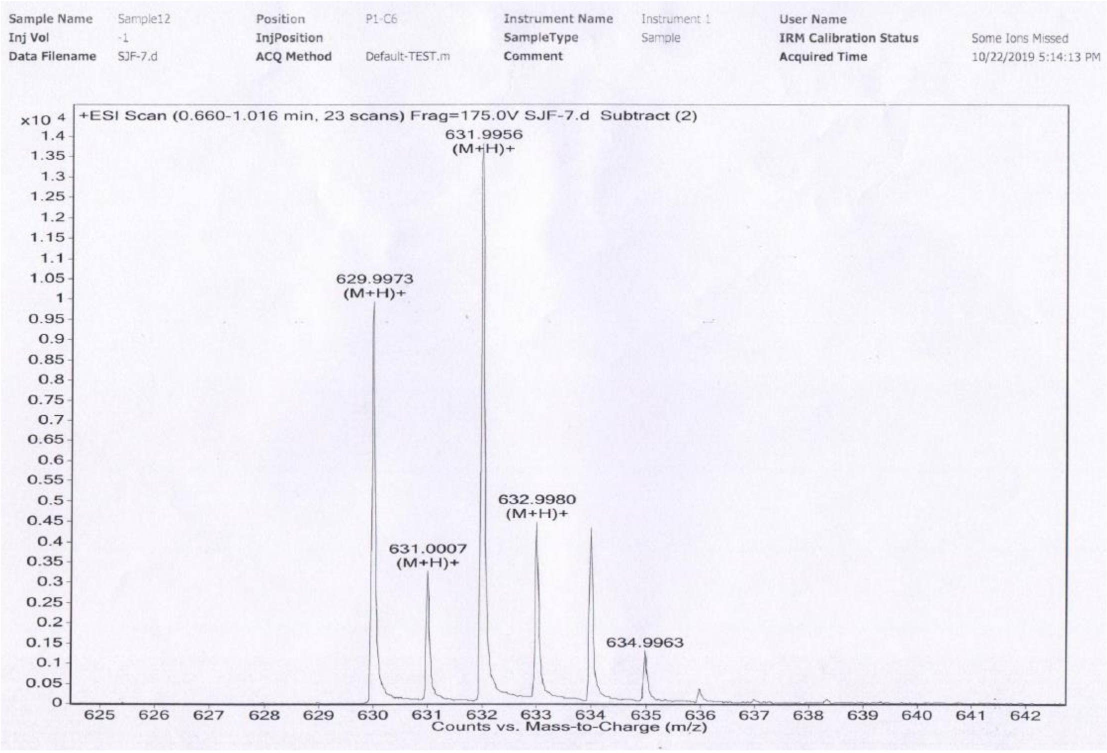

Figure S57. The HRMS spectrum of compound **8p**.

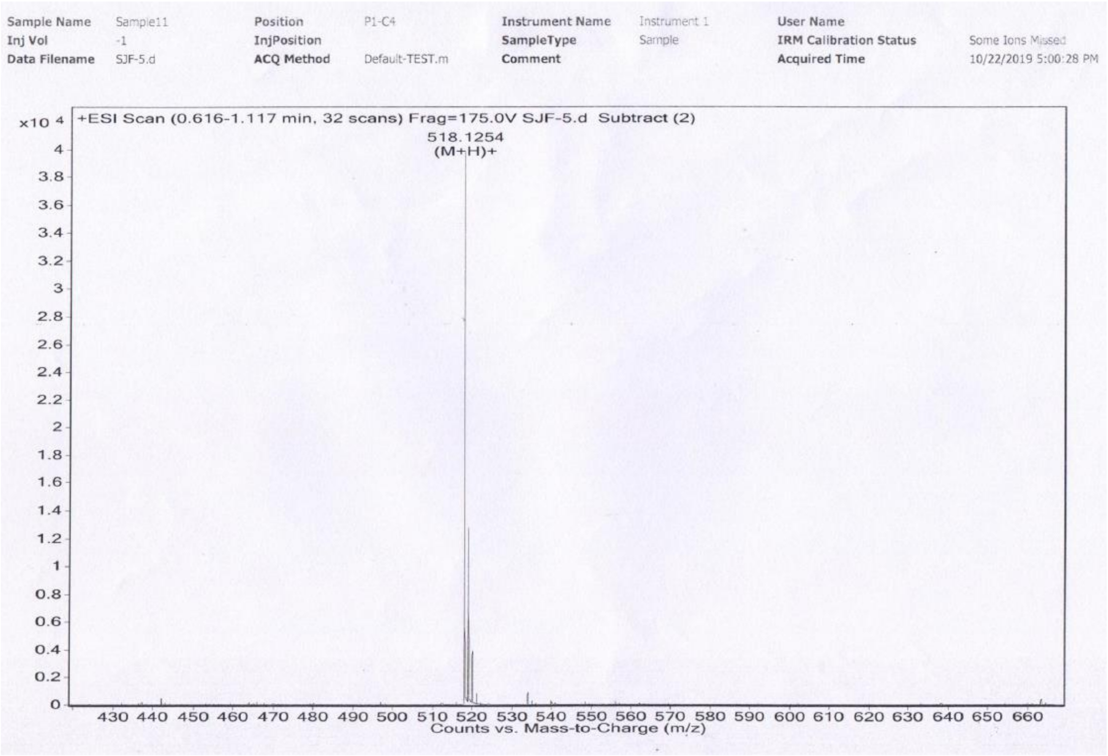

Figure S58. The HRMS spectrum of compound **8q**.

4. Biological activity test

The larvicidal/acaricidal activities of the title compounds against oriental armyworm (*Mythimna separata* Walker), corn borer (*Ostrinia nubilalis*), diamondback moth (*Plutella xylostella* L.), mosquito (*Culex pipiens pallen*), bean aphid (*Aphis craccivora*), and spider mite (*Tetranychus cinnabarinus*) were investigated ~~under~~ in relationship with the contrasts of **viii**, **ix**, and chlorantraniliprole in a greenhouse ~~according to the published literatures~~in accordance with the published literature precedents. [1-7] The assessments were made on a dead-alive basis, and lethality rates were corrected using Abbott's formula. [8] Each bioassay was repeated three times. Evaluations were based on a percentage scale of 0-100%, where 0% equals no activity, and 100% equals total kill. The standard deviations of the tested biological values were within ±5%.

4.1. Larvicidal activities against Mythimna separata Walker *(M. separata) and Ostrinia nubilalis (O. nubilalis)*

The larvicidal activities of the title compounds against oriental armyworm (*M. separata*) and

corn borer (*O. nubilalis*) were investigated ~~under the contrast of~~in relationship with the contrasts **viii**, **ix**, and chlorantraniliprole in a greenhouse, using a general procedure of the leaf-dip method.[1-3] The test was replicated based on statistical requirements at a temperature of 25 ^{o}C. At first, a solution of each test compound in acetone at a concentration of 200 mg·L^{-1} was prepared and then diluted to the required concentration with acetone. Leaf disks (5 × 1 cm) cut from fresh corn leaves were dipped into the test solution for 3-5 s. After air drying, the treated leaf disks were individually put into petri dishes (7 cm diameter). Each of these leaf disks was infested with 10 third-instar oriental armyworm or corn borer larvae. Percentage mortalities were evaluated 4 days after treatment. Each treatment was conducted three times. Leaves treated with acetone were used as blank controls.

4.2. Larvicidal activity against Plutella xylostella L. *(P. xylostella)*

The larvicidal activities of the title compounds against diamondback moth (*P. xylostella*) larvae were investigated in relationship with the contrasts~~under the contrast of~~ **viii**, **ix**, and chlorantraniliprole in a greenhouse, using a general procedure of the leaf-dip method.[2,4] The test was replicated based on statistical requirements at a temperature of 25 ^{o}C. At first, a solution of each test compound in DMF at a concentration of 200 mg·L^{-1} was prepared and then diluted to the required concentration with water. Leaf disks (6 × 2 cm) were cut from fresh cabbage leaves and then sprayed with the test solution for 3 s and allowed to dry. The resulting leaf disks were placed individually into glass tubes. Each disk was infested with 30 second-instar diamondback moth larvae. Percentage mortalities were evaluated 2 days after treatment. Each treatment was performed three times. Leaves treated with solvent were used as blank controls.

4.3. Insecticidal activity against Culex pipiens pallen (C. pipiens pallen) and Aphis craccivora (A. craccivora), and Acaricidal activity against Tetranychus cinnabarinus (T. cinnabarinus)

The larvicidal activities of the title compounds against mosquito (*C. pipiens pallen*) were evaluated by using the reported procedure.[4,5] The insecticidal activities against bean aphid (*A. craccivora*) were determined according to the known procedure in literature.[6] The acaricidal activities evaluations against ~~the adults of spider mites~~adult spider mite *(T. cinnabarinus)* were made adopting the reported procedure in literature.[5,7] Compounds **viii**, **ix** and chlorantraniliprole

were used as positive controls and tested under same conditions in a greenhouse.

5. Calcium imaging experiments

Effects of **8h**, **8i**, and **viii** on calcium ion channels in the central neurons isolated from the third-instar of *M. separata* were studied for the mode of action by calcium imaging techniques according to the references.[9,10]

5.1. Isolation of neural cells and calcium imaging experiments[9,10]

M. *separata* were initially obtained from shallot fields in Tianjin, China and reared indoors in climatic chambers on an agar-based semisynthetic diet at 27 ± 1 ^{o}C, 75 ± 5% relative humidity, and a LD 16 : 8 h photocycle. The insects were reared for two generations prior to the experiment. Third-instar larvae of *M. separata* were first anaesthetized with 70% ethanol and their thoracic and abdomen ganglia were removed and placed in saline. The thoracic and abdomen ganglia were transferred to a solution containing 0.3% trypsin for 6 min at 28 ^{o}C, plated into a 35 mm culture dish containing 1 mL of improved L-15 Leibovitz culture medium supplemented with fetal calf serum (15%, v : v) and then mechanically dissociated using a fire-polished Pasteur pipette. The cultures were maintained at 28 ^{o}C for 2 h to allow the cell to adhere to the dish. All procedures were carried out under sterile conditions.

Calibration of the fluorescence signal was achieved by using the method of Takahashi et al with modifications. Briefly, the attached neurons were rinsed twice in standard physiological saline [(mM): NaCl 150, KCl 4, $MgCl_2$ 2, $CaCl_2$ 2, HEPES 10, buffered to pH 7.0 and then incubated in the dark for 30 min at 28 ^{o}C in standard external saline containing the dye fluo-3 AM (10 µM) or incubated for 2 h with fluo-5 N AM (10 µM). After dye loading, cells were again rinsed in physiological saline twice. Calcium free extracellular fluid has the following composition (mM): NaCl 150, KCl 4, $MgCl_2$ 2, EGTA 2, Hepes 10, buffered to pH 7.0. The new compound was applied after 3 minutes of fluorescence recording. The original compound is eluted with calcium free extracellular fluid before the next compound is applied. Calcium ratio imaging studies were conducted using the imaging system coupled to an inverted fluorescence microscope with a Fluor 40× oil immersion objective (Olympus IX71). Cells were excited at 488 nm and the 530 nm

fluorescence emission acquired using a CCD (Image Pro-6.0).

5.2. Data analysis

Each experiment was repeated at least six times. The data were analyzed using GraphPad Prism on 7.0. Results were expressed as mean ± SD (n = number of cells).

Fluorescence values were expressed as F/F0, F0 being the resting (or baseline) fluorescence, and F the change in fluorescence from baseline after the compound application.

6. References

[1] Wang B-L, Zhu H-W, Li Z-M, Zhang X, Yu S-J, Ma Y, Song H-B. One-pot synthesis, structure and structure-activity relationship of novel bioactive diphenyl/diethyl(3-bromo-1-(3-chloropyridin-2-yl)-1H-pyrazol-5-yl)(arylamino)methylphosphonates. *Pest Manag Sci*. 2019;75:3273-3281.

[2] Wang B, Wang H, Liu H, Xiong L, Yang N, Zhang Y, Li Z. Synthesis and structure-insecticidal activity relationship of novel phenylpyrazole carboxylic acid derivatives containing fluorine moiety. *Chin Chem Lett*. 2020;31:739-745.

[3] Sun R, Li Y, Xiong L, Liu Y, Wang Q. Design, synthesis, and insecticidal evaluation of new benzoylureas containing isoxazoline and isoxazole group. *J Agric Food Chem*. 2011;59:4851-4859.

[4] Wang B-L, Zhu H-W, Ma Y, Xiong L-X, Li Y-Q, Zhao Y, Zhang J-F, Chen Y-W, Zhou S, Li Z-M. Synthesis, insecticidal activities, and SAR studies of novel pyridylpyrazole acid derivatives based on amide bridge modification of anthranilic diamide insecticides. *J Agric Food Chem*. 2013;61:5483-5493.

[5] Yu X, Liu Y, Li Y, Wang Q. Design, synthesis, acaricidal/insecticidal activity, and structure-activity relationship studies of novel oxazolines containing sulfone/sulfoxide groups based on the sulfonylurea receptor protein-binding site. *J Agric Food Chem*. 2016;64:3034-3040.

[6] Chen L, Huang Z, Wang Q, Shang J, Huang R, Bi F. Insecticidal benzoylphenylurea-S-carbamate: a new propesticide with two effects of both benzoylphenylureas and carbamates. *J Agric Food Chem*. 2007;55:2659-2663.

[7] Yu X, Zhang Y, Liu Y, Li Y, Wang Q. Synthesis and acaricidal- and insecticidal-activity evaluation of novel oxazolines containing sulfiliminyl moieties and their derivatives. *J Agric Food Chem*. 2019;67:4224-4231.

[8] Abbott WS. A method for computing the effectiveness of an insecticide. *J Econ Entomol*. 1925;18:265-267.

[9] Liu J-B, Li F-Y, Li Y-X, Zhang X-L, Hua X-W, Xiong L-X, Li Z-M. Synthesis, insecticidal evaluation and 3D-QSAR study of novel anthranilic diamide derivatives as potential ryanodine receptor modulators. *Pest Manag Sci*. 2019;75:1034-1044.

[10] Zhou Y, Feng Q, Di F, Liu Q, Wang D, Chen Y, Xiong L, Song H, Li Y, Li Z. Synthesis and insecticidal activities of 2,3-dihydroquinazolin-4(1H)-one derivatives targeting calcium channel. *Bioorg Med Chem*. 2013;21:4968-4975.
